# Phenolic Fingerprints of Spanish Olive Mill Wastewaters (Alpechin): A Step Toward Regional Valorization Through Antioxidant Recovery

**DOI:** 10.3390/antiox14111371

**Published:** 2025-11-18

**Authors:** Sergio Martínez-Terol, Emilia Ferrer, Pedro V. Martínez-Culebras, Houda Berrada, Noelia Pallarés, Jose Saez-Tovar, Luciano Orden, María R. Martínez-Gallardo, Ana J. Toribio, Francisco J. Barba

**Affiliations:** 1 Research Group in Innovative Technologies for Sustainable Food (ALISOST), Department of Preventive Medicine and Public Health, Food Science, Toxicology and Forensic Medicine, Faculty of Pharmacy, Universitat de València, Avenida Vicent Andrés Estellés s/n, 46100 Burjassot, València, Spain; sergio.martinez-terol@uv.es (S.M.-T.); emilia.ferrer@uv.es (E.F.); houda.berrada@uv.es (H.B.); noelia.pallares@uv.es (N.P.); francisco.barba@uv.es (F.J.B.); 2 Instituto de Investigación e Innovación Agroalimentaria y Agroambiental (CIAGRO-UMH), Universidad Miguel Hernández, Carretera de Beniel Km 3.2, 03312 Orihuela, Alicante, Spain; jose.saezt@umh.es (J.S.-T.); l.orden@umh.es (L.O.); 3 Department of Biology and Geology, CITE II-B, University of Almería, Agrifood Campus of International Excellence, ceiA3, CIAIMBITAL, 04120 Almería, Almería, Spain; mmg113@ual.es (M.R.M.-G.)

**Keywords:** olive mill wastewater, sludge, ponds, extraction, phenolic compounds, valorization

## Abstract

Olive mill wastewater (OMW), a by-product of olive oil extraction, poses significant environmental challenges due to its toxicity and heterogeneity. This study evaluates the phenolic and mineral composition of OMW and alpechin sludges from abandoned ponds in Spain, and establishes a standardized conventional method to recover phenolic fractions and promote their safe valorization as bioactive ingredients. Phenolic compounds were identified by triple-TOF-LC-MS/MS, and minerals and heavy metals were quantified by ICP-MS. Across thirteen ponds analyzed, samples from Cordoba, Tarragona, Alicante and Toledo showed higher phenolic levels, ranging from 7.2 g GAE/kg to 18.9 g GAE/kg, with methanolic extracts reaching 10.98–15.67 mg GAE/mL. Thirty-one phenolic compounds were identified, predominantly luteolin, apigenin, quercetin, and secoiridoid derivatives, notably hydroxytyrosol and tyrosol, supporting their functional potential as natural antioxidants. The mineral profile was dominated by K and Ca and showed negligible carryover to the phenolic organic fraction (<1%). Heavy metal concentrations in fresh OMW were 0.32–1.06 µg/kg for Cd and Hg and 9–43.9 µg/kg for As and Pb. In OMW sludge, they ranged between 0.033 and 0.19 mg/kg for Cd, 0.01 and 0.12 mg/kg for Hg, 5.45 and 8.06 mg/kg for As, and 4.45 and 23.70 mg/kg for Pb, whereas phenolic extracts contained only 0.15–21.50 µg/kg, remaining below EU food safety limits. This work presents one of the first integrated approaches to risk-benefit mapping of abandoned ponds in Spanish soils and advances extraction standardization by jointly considering functional potential, contaminant profiles, and matrix location.

## 1. Introduction

Olive and olive oil production is one of the most successful industries in the Mediterranean basin, with a rich historical legacy extending from ancient times to the contemporary era [1,2]. Annually, the region contributes with around 2.5 million tonnes of olive oil, representing 90% of the world’s production [1]. Unfortunately, olive oil production has always had a negative side effect on the environment. For example, due to the release of olive mill wastewater, OMW (“alpechin” in Spanish), estimated at an average of between 10 and 30 million m^3^ per year [3,4].

The OMW is a liquid emulsion with a strong odour and dark brown colour. Its composition is influenced by factors such as the variety of olive tree, the type of soil, the degree of maturity, the volume of water added and, especially, the method of extraction [4]. The widespread adoption of continuous two-phase centrifugation instead of traditional methods has profoundly influenced the reduction in water consumption and wastewater generation in the manufacturing of Extra Virgin Olive Oil (EVOO) [5,6,7]. Nevertheless, the resulting wastewater remains difficult to manage. Common practices such as storage in evaporation ponds or uncontrolled discharge into soils and rivers [8,9]. These wastes contribute to substantial environmental pollution, inhibiting plant growth, exacerbating soil hydrophobicity, and disrupting the delicate balance of ecosystems [3,10,11].

OMW typically contains 50–94% water, 4–16% organic matter (sugars, polysaccharides, tannins, organic acids, phenolic compounds and lipids), and 0.4–2.5% mineral salts [12,13]. Leveraging OMW for agricultural purposes holds promise as a valuable water and nutrient source, especially in water-scarce Mediterranean regions [3,10]. However, the presence of phenolic compounds significantly contributes to the phytotoxic and antimicrobial properties of OMW, thereby limiting its potential for agricultural utilization. Despite this, the recovery of valuable compounds such as specific polyphenols from these by-products and their conversion into higher-added-value products is one of the most efficient alternatives to manage this source of pollution [14,15,16].

Previous research on phenolic recovery from OMW has mostly employed conventional solvent extraction systems based on aqueous mixtures of methanol, ethanol, or ethyl acetate [6,17,18,19]. These methods typically target fresh OMW streams and rely on liquid–liquid or solid–liquid extraction, occasionally coupled with ultrasonication or direct acidification to improve recovery. Hernández-Fernández et al. (2023) [19] compared direct analysis of OMW with ultrasound-assisted extraction using MeOH:H_2_O (80:20 *v*/*v*), reporting a moderate improvement of 6.9% in TPC (from 98.3 ± 3.9 to 105.1 ± 2.8 mg GAE/g dw). Reported total phenolic contents usually range between 4–6 g GAE/L, with hydroxytyrosol and tyrosol as predominant species [19,20]. Likewise, De Marco et al. (2007) [17] showed that acidification to pH 2 prior to ethyl-acetate extraction increased phenolic recovery from 1.1 to 2.5 g tyrosol equivalents/L.

However, previous studies have mostly focused exclusively on fresh olive mill effluents, addressing yield or compound identification in isolation, with limited attention to abandoned evaporation ponds and their industrial valorization potential. This study optimizes a sequential conventional workflow (hexane defatting—controlled acidification—phenolic extraction) that is matrix-agnostic, i.e., applicable to pond liquids (recent eluates or vegetation waters), evaporation sludges, and commercial OMW. This approach allows comprehensive mapping of phenolic diversity and contaminant profiles across multiple locations pounds, providing a realistic analytical basis for valorization.

Polyphenols are widely recognized as natural bioactive with potential industrial applications, due to their antioxidant, antimicrobial, cytotoxic and anti-inflammatory properties [21]. Importantly, natural compounds with bioactive properties are increasingly being used in the agri-food sector to replace synthetic ones [2,13]. Consequently, the recovery of phenolic compounds from OMWs not only provides an economic opportunity but also decreases the environmental impact [10]. The main phenolic compounds found in OMW are hydroxytyrosol, tyrosol, oleuropein, apigenin, cyanidins, verbascoside and phenolic acids such as caffeic, gallic, vanillic and cinnamic acids [21,22,23,24]. These molecules have shown anti-inflammatory action [25], antiangiogenic and chemo-preventive effects [26,27]. Accordingly, the use of phenolic OMW extracts has been demonstrated in several applications in the cosmetics [21,28] and food sectors [29], where they could be used as active ingredients in dietary supplements and functional foods. Therefore, olive-derived by-products represent a sustainable source of high-value bioactives, supporting the development of functional formulations and extending food shelf life [3,13,29,30]. Recent reports indicate the presence of up to 3000 mg/kg of polyphenols in foods produced without organic solvents [31,32].

The aim of this study is to optimize conventional extraction methods to obtain phenolic fractions from OMW samples collected in abandoned ponds across the Mediterranean and southern Spain. The total polyphenol content (TPC), phenolic and mineral composition, and heavy-metal levels were determined to evaluate both the functional potential and the safety profile of the resulting extracts. Collectively, these analyses establish an analytical and process baseline for concentration, characterization, and selection using conventional extraction to position OMW and evaporation-pond sludges as a reliable source matrix for phenolic recovery, facilitating their subsequent valorization and within bioeconomy pathways.

## 2. Materials and Methods

### 2.1. Samples

Eight olive mill wastewater (OMW) samples and five OMW sludge samples were analysed. These samples were collected from abandoned ponds located in major olive oil producing regions in the Mediterranean area and southern Spain (Extremadura, Andalucia, Castilla la Mancha, Comunitat Valenciana and Cataluña) [33]. Sampling codes (TED-2 to TED-16) refer to the nearest olive mill and the specific pond location (see Figure S1 for site distribution). Traceability details (sampling date, municipality/province, matrix, storage temperature, and time elapsed before analysis) are compiled in Table S1 to ensure reproducibility and transparency.

### 2.2. Experimental Design for the Valorization of OMW

All samples were manually homogenized and stored at −20 °C before analysis. pH was determined directly in the OMW samples and a 1:3 (*w*/*v*) water soluble extract of the OMW sludge samples, and moisture was determined by weight loss at 40 °C for 48 h. The OMW samples were centrifuged at 3000× rpm for 10 min to remove most of the solid particles. The supernatant fats and solid precipitates were then removed, and the remaining aqueous phase was subjected to the defatting process.

#### 2.2.1. OMW Defatting

Defatting was carried out using n-hexane at 20–25 °C, adapting the solvent ratio and contact time to the lipid content of each matrix. For liquid OMW, the hexane-to-sample ratio ranged from 1:4 to 1:10 (*w*/*w*), with agitation for 10 min in a single step. For OMW sludge, the ratio ranged from 1:3 to 1:4 (*w*/*w*), with duplicate extractions of 30 min each, to ensure complete lipid removal. Phase separation was facilitated by settling and centrifugation (3000 rpm, 10 min). The organic phase was then evaporated under vacuum in darkness using a rotary evaporator until a constant dry weight was achieved. All experiments were carried out at a room temperature, approximately 25 °C [34].

#### 2.2.2. Evaluating the Influence of pH

The OMW samples were acidified to pH 2 by the addition of 3M HCl, which has been shown in previous research to promote the solubilization of phenolics in the organic phase [34,35,36]. To further evaluate and confirm the effect of pH on the phenolic extraction of the OMW samples, the influence of pH on the extraction of total phenolic compounds was investigated at pH 2, natural pH (4.5–5.5) and pH 12.

#### 2.2.3. Solid/Liquid Extraction

**Figure 1:**
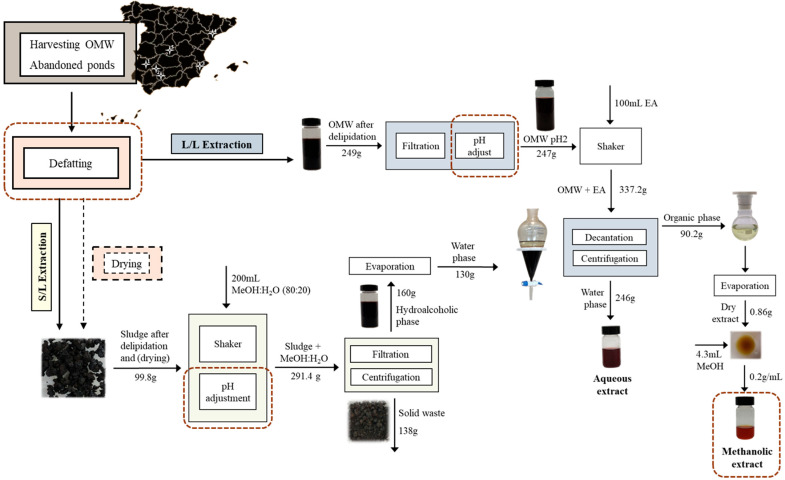
Schematic of the conventional olive mill wastewater (OMW) extraction process.

Phenolic fractions of OMW sludge were obtained by stirring the sample with a mixture of methanol and water (80:20 *v*/*v*) for 24 h at a constant temperature of 40 °C in the dark (Figure 1) [37]. The mixture was then centrifuged at 3000× rpm for 10 min to separate the hydroalcoholic phase from the solid. This was followed by vacuum filtration of the liquid phase and evaporation of the methanol from the hydroalcoholic mixture as part of the continuous process to perform the liquid/liquid extraction. This methodology is in line with the proposal of ref. [38], to optimize the recovery of phenolic compounds.

#### 2.2.4. Liquid/Liquid Extraction

In the initial phase of the extraction process, the defatted liquid samples were subjected to vacuum filtration. Ethyl acetate has been highlighted as the most efficient and sustainable solvent for the recovery of phenolic compounds in conventional procedures, as evidenced by several studies [6,37,39,40,41]. However, being aware of the need to limit its use due to its toxic properties, the ratio was adjusted to between 20 mL and 30 mL per 150 mL of aqueous phase, and this process was repeated three times to optimize the extraction. A contact time of 2 h was selected between the phases, determined as the optimal duration for achieving maximum extraction, as suggested by [42].

Phase separation was facilitated by centrifugation (3000 rpm, 5 min). The organic fraction (FO) was concentrated on a rotary evaporator under vacuum (160 mbar, 35 °C) in the dark. When required to ensure complete solvent removal, evaporation was continued at 100 mbar and 50 °C to constant dry weight (mass variation < 0.5% between consecutive weighings). The dried residue was weighed and reconstituted in methanol (2 to 8 mL). This solution, together with the corresponding aqueous fraction (AF), was stored at −20 °C. The workflow is summarized in Figure 1.

### 2.3. Chemical Analysis of the Extracts

#### 2.3.1. Total and LC/MS/MS Determination of Phenolic Profile

The total phenolic content (TPC) of the different OMW fractions was determined by the Folin–Ciocalteu assay following [43,44]. A 2% (*w*/*v*) Na_2_CO_3_ solution and the 50% (*v*/*v*) Folin–Ciocalteu reagent were prepared. Gallic acid (Sigma-Aldrich, Steinheim, Germany) was used for calibration. Aliquots of 0.10 mL extract were mixed with 3.00 mL 2% Na_2_CO_3_ and 0.10 mL FC reagent, incubated 60 min at room temperature in the dark, and read at 750 nm (Perkin-Elmer UV/Vis Lambda 2, Jügesheim, Germany). Results are expressed as gallic acid equivalents per gram of fresh matter (g GAE/kg FM). As the Folin–Ciocalteu response reflects total reducing capacity, the TPC value obtained using this method may include contributions from non-phenolic reducing substances. Therefore, values may be overestimated, particularly in fractions rich in reducing sugars.

The yield of phenolic compounds in fresh OMW was calculated by summing the phenolics recovered in the extracts and those remaining in the AF. These determinations were made relative to the phenolic mass measured in each fraction, the volume of each fraction, the original sample mass and moisture content. Within this experimental framework, the cumulative sum represents the total phenolic content of the original fresh OMW sample. However, for OMW sludge, the sum of fractions does not represent the total phenolic content, as TPC in the solid matrix cannot be fully determined under these extraction conditions.

The phenolic profile was evaluated according to the methodology described by several authors [45,46] using a TripleTOF™ 5600 LC/MS/MS system (AB SCIEX) coupled to an Agilent 1260 Infinity (Agilent Technologies, Waldbronn, Germany). The chromatographic separation was performed on a Waters UPLC C18 column (1.7 μm, 2.1 × 50 mm, Acquity UPLC BEH C18, Waters, Cerdanyola del Vallès, Spain).

An injection volume of 5 μL and a flow rate of 0.4 mL/min were used. The mobile phase consisted of a 0.1% (*v*/*v*) aqueous formic acid solution (A) and a 0.1% methanolic formic acid solution (B). The elution gradient of the mobile phase was programmed as follows: 90% (A) and 10% (B) from 0 to 13 min, 100% (B) from 13 to 15 min and 90% (A) and 10% (B) from 15.1 to 22 min.

MS acquisition was carried out in negative electrospray ionization (ESI−) mode over an m/z range of 80–1200. External calibration was applied prior to analysis. Information-Dependent Acquisition (IDA) was used with a TOF-MS survey scan and product-ion MS/MS. Source and acquisition parameters were: ion spray voltage (ISV) −4500 V, declustering potential (DP) −90 V, source temperature 400 °C, curtain gas 25 psi, ion source gas 1 (GS1) 50 psi, and ion source gas 2 (GS2) 50 psi. Collision energy (CE) was −25 V for the TOF-MS survey and −50 V for product-ion MS/MS. IDA criteria included an intensity threshold >100 cps, a mass tolerance of 50 mDa, and dynamic background subtraction.

Compound identifications were assigned at Level 1 when confirmed with co-injected standards, and at Level 2 (tentative) when supported by MS/MS library matching (diagnostic fragments), accurate mass (±5 ppm) and retention time consistency (±0.2 min) [47]. Full per-compound criteria are provided in Supplementary Table S2.

#### 2.3.2. ICP-MS Evaluation of Mineral and Heavy Metal Contents

The mineral profile (Mg, P, K, Ca, Fe, Cu, and Zn) and the presence of heavy metals, (As, Cd, Hg, and Pb) were analysed in the OMW samples, the extracts, and the solid residue after extraction. Sample digestion was carried out using a microwave accelerated reaction system (Ethos Easy High-Pressure Microwave, Milestone, Sorisole, Italy). For 10 mg of solid sample and 1 mL of liquid sample, digestions were performed with 1 mL of 69% HNO_3_, applying a power of 800 W and reaching a maximum temperature of 180 °C for 15 min. The final volume was then adjusted to 5 mL with ultrapure water.

An inductively coupled plasma-mass spectrometer (ICP-MS, Agilent 7900, Agilent Technologies, Santa Clara, CA, USA) was used for elemental identification and quantification. The operating conditions were as follows: argon plasma gas flow (15.0 L/min), carrier gas (1.0 L/min), reaction gas (helium), nebulizer pump speed (0.30 rps), Radio Frequency (RF) power (1550 W) and RF coincidence (1.80 V). Internal standard solutions of Ge, Rh and Ir (ISC Science) at 20 µg/g were used to correct for signal variations due to matrix and instrumental drift.

Quantification of As, Cd and Pb was carried out using a standard calibration curve with concentrations ranging from 0 to 1000 µg/L, while for Hg a standard calibration curve ranging from 0 to 100 µg/L was used. The standards of 0∼10,000 μg/L were employed for quantitative analysis of minerals. Distilled water was used as a blank and the concentrations of the metals in the digested blank were subtracted from samples values. Calibration acceptance criteria: all calibration curves showed correlation coefficients (R) ≥ 0.9999, with RSD ≤ 5% for each calibration level. A post-sequence calibration verification confirmed deviations within ±5% of the initial standard response.

LOQ values ranged from 0.2 to 10 µg/L and the corresponding LOD values from 0.07 to 3.3 µg/L, depending on the element analysed. These limits are representative for both mineral and heavy metal determinations, as identical digestion and dilution protocols were applied to all matrices. Results are reported as µg/kg. Concentrations in liquid samples and extracts refer to a fresh-matter basis (FM), whereas those in the solid residue are expressed on a dry-matter basis (DM), as its moisture content depends on extraction conditions rather than the native matrix.

#### 2.3.3. Statistical Analysis

For data analysis, all measurements were performed in triplicate and results were expressed as mean ± standard deviation. Statistical evaluation was performed using GraphPad Prism 9 (GraphPad Software, San Diego, CA, USA), with analysis of variance (ANOVA) applied to the TPC, matrix status, minerals, and heavy metals data parameters to assess differences between the different TED codes. Multiple comparisons were performed using Tukey’s test (for one-way ANOVA) and Sida’k (for two-way ANOVA). Student’s *t*-test was used to compare physicochemical parameters for each TED code. A significance level of *p* < 0.05 was used for all statistical analyses.

## 3. Results and Discussion

### 3.1. Characterization and Pretreatment of OMW

Our samples showed a strong odour, dark brown colour, acid pH (mean value of 5.35), high moisture content (mean value 69%) and high total phenol (TPh) content (mean value 7.3 g GAE/kg fresh OMW). We classified the material by matrix state, enabling a separate analysis of OMW and OMW sludge. In general, the OMW samples had a slightly more acidic pH, more pronounced reddish tones and a lower TPh concentration than OMW sludge samples (Table 1, Figure S1). Table 1 shows the initial characterization of OMW samples analysed in comparison with other studies. The parameter values between liquid and solid alpechin were different, but like those found in other studies. The pH falls within the OMW range (4.2–6.5); moisture is consistent with 70% reported for Greek and Spanish OMW; and our TPC lies between typical Italian (6.2 g GAE/kg) and higher Moroccan (12 g GAE/kg) values. In a Spanish production context, this study advances current knowledge by analysing liquid OMW and sludge from the same geographical area under a common workflow, providing matrix-specific data rarely reported in Mediterranean studies.

**Figure 2:**
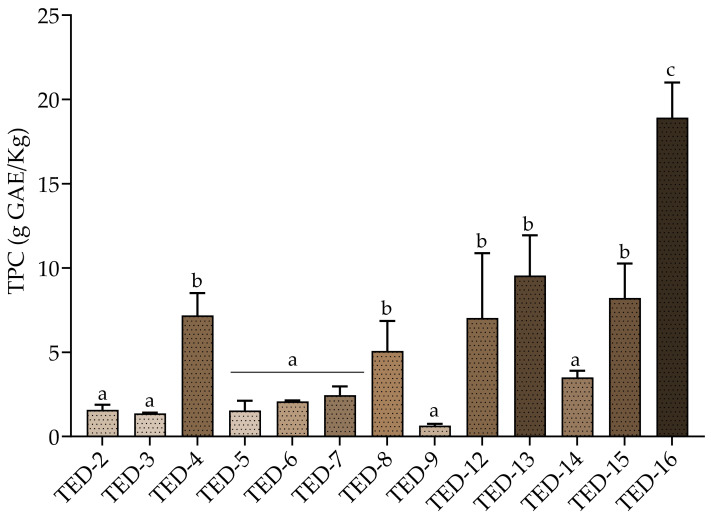
Total phenolic content (TPC) in fresh olive mill wastewater (OMW) samples, expressed as g gallic acid equivalents (GAE) per kg of fresh matter (FM), obtained under conventional extraction at natural pH. (n = 3). Small letters: indicate significant difference between the different TED for TPC (one-way ANOVA followed by Tukey’s test, *p* value < 0.05).

A wide variety of samples were found according to their content of TPC, with values from 0.6 g GAE/kg (TED-9) to 18.9 g GAE/kg (TED-16) (Figure 2). On one hand, the OMW samples from Córdoba, Alicante, Tarragona, and Toledo, corresponding to codes TED-4, TED-13, TED-15 and TED-16, respectively, showed high contents of TPC ranging from 7.2 g GAE/Kg to 18.9 g GAE/Kg. Notable among these, the sample TED-16 from Toledo displayed the highest TPC value (18.9 g/kg). On the other, samples TED-2, TED-3, TED-5, TED-6, TED-7, TED-9 and TED-14 showed a lower TPC content in comparison with the later ones (Figure 2). The observed variability in phenolic richness among ponds is primarily attributable to matrix state (liquid vs. sludge) and the degree of evaporation, but may also reflect the pond’s operational status. Ponds that have not received new effluents for several production seasons tend to form more earthy and heterogeneous sludges with lower phenolic concentration (e.g., TED-12, TED-14), whereas active ponds periodically recharged with fresh OMW produce darker, more homogeneous sludges enriched in soluble and phenolic compounds (e.g., TED-16).

A qualitative and quantitative comparison of the methanolic extracts and AFs obtained from each conventional extraction [38] was carried out with the aim of clarifying their respective contributions to the total phenolic content in fresh OMW. These percentages (see Figure S2) reflect the apparent distribution of Folin–Ciocalteu-responsive compounds between the methanolic and aqueous phases under the specified extraction protocol. They should be interpreted as operational yields in relation to the extraction matrix rather than as exhaustive mass balances. Triplicate extractions of eight liquid OMW samples (TED-2, 3, 4, 5, 6, 7, 8 and 9; 24 methanolic extracts and 24 AFs) revealed that the AF retained 76.9–90.5% of the measured TPC in the fresh matrix, whereas the methanolic extract represented the transferred phenolic portion in the organic phase. For OMW sludge (TED-12, 13, 14, 15, 16; n = 15 + 15), the AF retained 68.0% ± 5.9. Thus, when expressed on a matrix-normalized basis, the procedure recovers approximately 16.3% of polyphenols from liquid OMW and >30% from sludge into the methanolic extract, whereas the remainder remains in the aqueous phase of the fresh matrix after partitioning. Estimates in sludge may be upward-biased due to methodological limitations of TPC in solid matrices (see Section 2.3.1), and apparent increases in the aqueous phase can reflect residual interference from reducing sugars and highly polar glycosylated phenolics [53].

Focusing on the methanolic extracts obtained using this protocol, the phenolic concentrations were 8.72 ± 1.22, 11.58 ± 0.27 and 15.67 ± 1.44 mg GAE/mL for TED-4, TED-8 and TED-16, respectively. On a matrix basis, these correspond to 1.49 ± 0.12, 0.54 ± 0.27 and 3.63 ± 0.40 g GAE/kg of fresh OMW. In this framework, the methanolic extract constitutes the recovered phenolic stream, whereas the AF reflects the residual phenolic distribution post-extraction; both are complementary process streams with distinct analytical and valorization objectives.

### 3.2. Hexane Defatting Treatment

Hexane extraction showed remarkable efficiency in reducing fats and oils compared to the untreated sample. This procedure not only streamlined sample handling but also facilitated sample drying, thereby improving sample preservation and readiness for subsequent analysis.

However, this step can remove lipophilic compounds of interest, such as squalene, tocopherols and sterols, which are known constituents of OMW and are often lost in processing waste streams [54]. In addition, hexane may co-extract phenolics with higher hydrophobicity (e.g., p-hydroxybenzoic acid, cinnamic acid and coumaric acid), as suggested by activity coefficients in non-polar solvents such as diethyl ether [38,55].

TPC was quantified in hexane defatting solutions from three representative samples: sludge (TED-16) and liquid OMW (TED-4 and TED-8). Defatting removed 16.4 ± 1.7% of TPC in sludge and 6–11% in liquid OMW (Figure S3). Lower apparent losses in liquid OMW may reflect water-mediated hydrogen bonding and electrostatic interactions that retain phenolic compounds in the aqueous phase [38], whereas the higher non-polar fraction and reduced water activity in sludge may favour phenolic partitioning into hexane. Targeted profiling of the hexane phase is required to determine which phenolic compounds are being co-extracted.

### 3.3. Effect of pH on TPC Extraction

**Figure 3:**
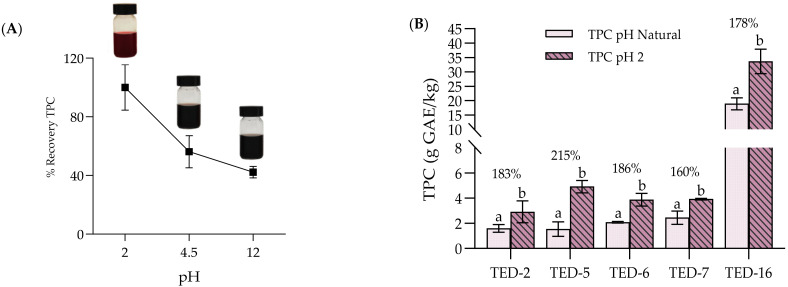
(**A**) Percentage recovery of total phenolic content (TPC) in fresh olive mill wastewater (OMW, sample TED-16) under different pH conditions (2, 4.5 and 12) (n = 3). (**B**) TPC (g GAE/kg FM) at pH 2 versus each sample’s natural pH (4.6–6.1) for TED-2, TED-5, TED-6, TED-7, and TED-16 (n ≥ 3 per condition). Small letters: indicate significant difference between pH conditions within the same samples (Student’s *t*-test, *p* value < 0.05).

The effect of pH on the determination of the TPC in the OMW samples after the extraction process is shown in Figure 3. Figure 3A shows the variation of total phenol recovery for TED-16 at pH 2, pH 4.5 and pH 12, while Figure 3B compares pH 2 versus the natural pH (4.6–6.1) for TED-2, TED-5, TED-6, TED-7, and TED-16 (n ≥ 3 per condition). Acidification to pH 2 resulted in a significant increase in measured TPC, often exceeding 60% relative to the natural pH of the samples tested (see Figure 3B). In contrast, alkaline pH (approximately 12) led to lower TPC compared with natural pH (see Figure 3A). Acidification can act as a pre-treatment that promotes the recovery of non-extractable polyphenols (NEP) retained in the matrix, enhances phenolic stability during extraction, and reduces underestimation, as discussed by authors [41,56]. The higher TPC observed under acidic conditions is also consistent protonation of the phenolic -OH groups and the acid-catalysed hydrolysis of glycosidic and ester linkages in complex phenolic (e.g., verbascoside and oleuropein), which releases lower-molecular-weight aglycones (e.g., hydroxytyrosol and tyrosol/caffeic acid derivatives) and increases the proportion of Folin-reactive compounds [42].

It is also interesting to note that a variation in the colour of the samples in relation to pH was observed (Figure 3A). In more acidic environments, a more reddish colour was observed compared to more basic environments. This colour change has been associated with an oxidation of the phenolic compounds present, particularly indicated by the presence of tannins and anthocyanins, which are characterized as pH sensitive pigments [36,41].

### 3.4. LC/MS/MS Analysis and Characterization of Phenolic Profile

The Triple-TOF-LC-MS/MS method (see Section 2.3.1) allowed the characterization of the phenolic fingerprint in three OMW samples (TED-4, 6 and 8) and three OMW sludge samples (TED-13, TED-15 and 16) selected according to their higher polyphenol content. A total of 31 phenolic compounds were identified and classified into six structural families: anthocyanins, lignins, phenolic acids, flavonoids, flavones and oleuropein derivatives (Table 2).

The MS/MS identification criteria, including precursor ion, diagnostic fragments, retention time, mass error (ppm) and confirmation with co-injected standards, are detailed in Supplementary Table S2. This additional dataset provides full analytical traceability and supports the structural assignments reported here. Representative chromatograms for key compounds within each family are shown in Figure S5.

**Figure 4:**
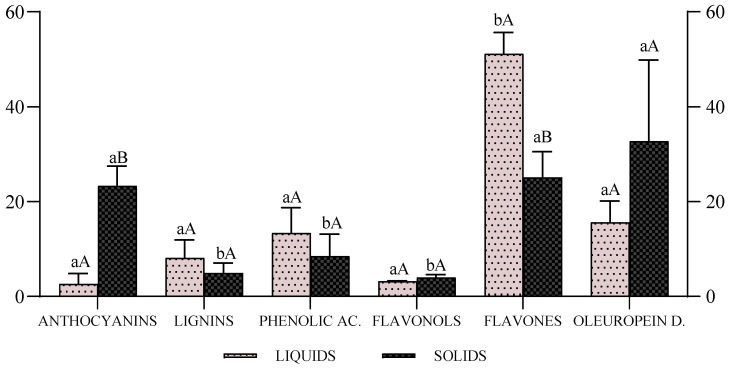
Distribution of phenolic compound classes by structural group and matrix state in olive mill wastewater (OMW) from TED-4 and TED-8 (liquid OMW) and TED-13 and TED-16 (OMW sludge). ^ab^ Different letters (for the same type of OMW) indicate significant differences among phenolic classes. ^AB^ Different letters (for the same group of phenolic compounds) indicate significant differences between matrix states (liquid vs. sludge). Statistical differences were determined by two-way ANOVA with Šidák’s multiple-comparison test (*p* < 0.05).

Figure 4 illustrates the relative distribution of phenolic subclasses in liquid OMW (TED-4 and TED-8) and OMW sludge (TED-13 and TED-16). Flavones dominated the liquid matrices (48.1–54.3%), whereas the contribution of flavones to the sludge was markedly lower (21.3–28.9%). Conversely, anthocyanins were highly concentrated in sludge (20.4–26.3%) and remained low in liquids (1.11–4.18%). Oleuropein derivatives were also more abundant in sludge (20.6–44.8%) than in liquids (12.4–18.8%). Phenolic acids and lignin-derived phenolic compounds contributed more to the liquid samples (13.3% and 8.11%, respectively), whereas flavonols remained at low levels in both matrices (3–4%).

The anthocyanin fraction comprised mainly glycosides of cyanidin, delphinidin, malvidin, and petunidin, typically O-glycosylated at the C3 position of the C-ring. Among flavonoids, luteolin, apigenin, and quercetin species (aglycones and glycosides) were consistently detected across OMW samples, with a higher relative proportion of luteolin and apigenin in the liquid extracts (Figure 4). In contrast, OMW sludge showed a higher presence of intermediate phenolic compounds, including 11-methyl oleoside ester, 3,4-DHPEA-EDA and acetylated derivatives of hydroxytyrosol (3,4-DHPEA-AC) and tyrosol (p-HPEA-AC), than liquid OMW.

Within the oleuropein derivatives group, the presence of oleuropein and ligustroside, and their secoiridoid derivatives was confirmed [57]. These compounds are widely recognized for their anti-inflammatory, antioxidant and antimicrobial properties [21,58,59]. Among them, the aglycone forms 3,4-DHPEA-EA and p-HPEA-EA were identified in several samples (TED-4, TED-6, TED-13 and TED-16), while the dialdehyde derivative 3,4-DHPEA-EDA appeared predominantly in TED-6, TED-8 and TED-13/15, consistent with the β-glucosidase mediated degradation of oleuropein [57]. Mild acidification during extraction may accelerate such oleuropein degradation, explaining the increased levels of hydroxytyrosol and tyrosol, in line with by [17].

The recovery of secoiridoid-type compounds in the phenolic extract (FO), expressed relative to the total phenolic signal (FO + AF), was 72.5% (TED-4), 88.7% (TED-6), 81.9% (TED-8), and exceeded 90% in the sludge matrix (e.g., TED-16, 97.4%). These values reflect the selective enrichment of this phenolic class in the FO, supporting the suitability of the conventional workflow for concentrating secoiridoids. The comparatively lower recoveries in liquid OMW are consistent with the greater relative contribution of the AF in these matrices (see Figure S2).

The greater representation of oleuropein-derived compounds in the sludge extracts including 3,4-DHPEA-EA, p-HPEA-EA, oleoside-11-methyl ester, 3,4-DHPEA-EDA, 3,4-DHPEA-AC, p-HPEA-AC, hydroxytyrosol and tyrosol, can be attributed to the progressive hydrolysis and enzymatic cleavage of complex secoiridoids during storage [60]. These compounds also higher solubility in the hydroalcoholic solid–liquid extraction step, which favours their recovery in the phenolic extract. By contrast, flavonoids exhibit lower extractability under these conditions and are partly retained in the solid fraction.

Overall, the compositional trends observed in this study are consistent with previous LC–MS/MS investigations of Mediterranean OMW, where flavones, phenolic acids, and secoiridoids constitute the dominant phenolic families [30,35,61]. However, those studies focused exclusively on fresh liquid effluents, without considering the compositional differences that emerge after long-term storage or evaporation. In contrast, the present study includes both liquid OMW and evaporation sludges, allowing a matrix-dependent comparison of phenolic profiles. Notably, oleuropein, hydroxytyrosol, and tyrosol were detected across all TED samples; these phenolics have well-documented antioxidant and anti-inflammatory and antiradical activities and demonstrable shelf-life-extending effects in food systems [62], underscoring the functional relevance of the recovered fractions. These compositional characteristics support the use of the extracts as functional ingredients for industrial applications and highlight the interest of these matrices for subsequent valorization via conventional or innovative extraction technologies that favour food-grade hydroalcoholic systems over less sustainable solvents (e.g., ethyl acetate).

### 3.5. Mineral Profiles and Contents

**Figure 5:**
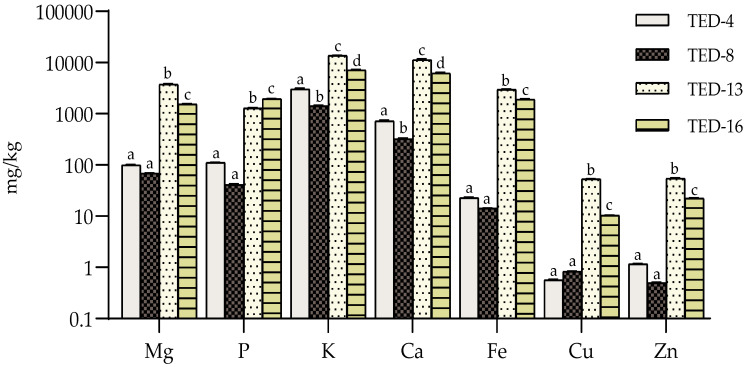
Mineral content in untreated fresh olive mill wastewater (OMW) for TED-4, TED-8, TED-13 and TED-16, expressed in mg/kg FM. Presented on a base-10 logarithmic scale. (n = 3) Small letters: indicates a significant difference in mineral concentrations among TED samples (one-way ANOVA followed by Tukey’s test, *p* value < 0.05).

The results of the analysis of the mineral content of the OMW samples are shown in Figure 5. The predominant presence of potassium and calcium in the OMW is highlighted, although significant levels of phosphorus (P), magnesium (Mg) and iron (Fe) were observed compared to those found in other studies [4,12,13]. The OMW sludges analysed (TED-13 and TED-16) showed statistically significant higher mineral concentrations compared to the OMWs (TED-4 and TED-8). In particular, the solid alpechin TED-13 showed a higher content of all the minerals analysed.

The high concentration of soluble salts, especially potassium (7.1–13.7 g/kg) and calcium (6.2–18.4 g/kg) in the OMW sludge is due to their significant content in the olive oil production effluent and the calcareous soils typical of southern Spain [12,13,48]. The percolation of minerals through the permeable substrate of the ponds, together with the evaporation of the pond water, results in higher concentrations in the sludge.

Regarding the mineral content of the phenolic extracts obtained, the conventional extraction recovered less than 1% of the mineral content present in the FMs. In contrast, the AFs showed mineral levels comparable to those of the original OMW; however, the apparent partitioning depended on the matrix state, since this determines the extraction pathway and thus what each AF represents within the procedure. In liquid OMW (AF-OMW), mineral levels typically exceeded 70% relative to the initial matrix (see Figure S4), particularly for Zn, Ca, K and Mg. In sludge-derived samples, where an earlier hydroalcoholic solid–liquid step precedes the aqueous fraction, the corresponding AFs generally remained below 50% relative to the initial matrix. Potassium (K) exhibited the greatest apparent transfer (up to 56.5%), consistent with leaching [12].

It highlights the importance of considering OMW as an important source of high-added-value compounds, particularly in terms of mineral nutrients such as potassium and phosphorus. These could be efficiently used as agricultural fertilizers and food additives, provided that their polluting power is effectively managed. This perspective promotes more sustainable practices and proper management of these industrial by-products, contributing to environmental sustainability and responsible resource use [4,12,13].

### 3.6. Heavy Metal Profiles and Contents

**Figure 6:**
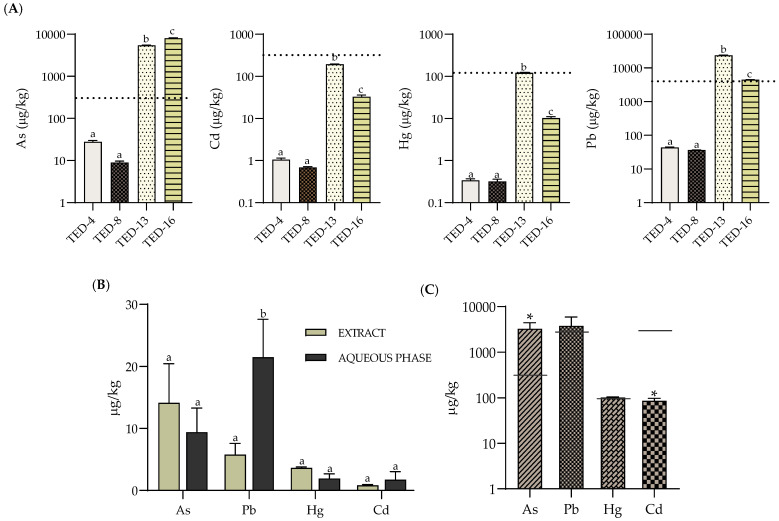
Heavy-metal content for TED-4, TED-8, TED-13, TED-16. (**A**) Untreated fresh olive mill wastewater (OMW) expressed in µg/Kg FM. Presented on a base-10 logarithmic scale. (n = 3). Small letters: indicate a significant difference in heavy metals concentrations among the analysed OMW samples (one-way ANOVA followed by Tukey’s test, *p* value < 0.05). Dashed lines: maximum limits set for foodstuffs by legislation for each heavy metal. (**B**) Average heavy metal content in the organic extract and aqueous fraction (AF) in the same samples (µg/Kg FM). (n = 4). Small letters: indicate significant difference indicate significant differences between extract and AF within each heavy metal (two-way ANOVA with Šidák test, *p*-value < 0.05). (**C**) Average heavy-metal content in the post-extraction solid residue (sludge) (µg/kg DM). (n = 6) * Indicates significant difference (two-way ANOVA with Šidák test, *p*-value < 0.05) for heavy metal between the solid waste and the maximum limits set for foodstuffs by legislation for each heavy metal.

The contents of As, Cd, Hg and Pb in the fresh OMW, as well as in the extracts and the resulting solid residue after extraction, are shown in Figure 6. The concentration ranges, expressed in µg/kg, for the fresh liquid matrices (OMW) vary from 0.32 to 1.06 for Cd and Hg and from 9 to 43.9 for As and Pb. It should be noted that no significant differences were observed between samples TED-4 and TED-8. On the other hand, in the fresh OMW sludge, the concentration ranges were significantly different with respect to the liquid matrices, which ranges (mg/kg) were from 0.033 to 0.19, 0.01 to 0.12, 5.45 to 8.06, 4.45 to 23.70 for Cd, Hg, As and Pb, respectively. Moreover, significant differences were also observed between sludge samples themselves (TED-13 vs. TED-16), indicating matrix-specific accumulation patterns. Overall, Pb was the most abundant heavy metal, followed by As.

In the phenolic extracts obtained by conventional extraction, heavy-metal contents ranged from 0.15 to 21.50 µg/kg (Figure 6B). These concentrations lie well below the maximum levels established for foodstuffs under EU Regulation (EU) 2023/915, confirming the favourable toxicological profile of the extracts for their prospective use as food/nutraceutical uses [63]. In liquid OMW, most of the mineral and heavy metal content remained in the aqueous fraction (AF), exhibiting concentrations similar to those of the initial matrix and reflecting the low transfer of these elements to the phenolic extract. In contrast, the AFs derived from OMW sludge showed even lower metal content due to the minimal carryover during the preceding hydroalcoholic solid–liquid extraction step, where heavy metals were almost entirely retained to the solid residue. This distribution pattern is consistent with the partitioning previously observed for mineral elements and reinforces the suitability of both fractions for differentiated valorization.

Consistent with their predominance in the fresh matrix, Pb and As were the most enriched metals in the post-extraction solid residue, reaching up to 3.78 mg/kg DM (Pb) and 3.27 mg/kg DM (As) (Figure 6C). Although these concentrations remain below the legal thresholds established for materials intended for land application (Royal Decree 1051/2022 and Directive 86/278/EEC), which set limit values of <10, 750, 10 and 40 mg/kg DM for Cd, Hg, As and Pb, respectively [64,65], any prospective agronomic reuse must be conditioned upon leaching and mobility assessments to ensure that metal release remains within permissible limits and does not compromise soil or crop integrity [66,67]. In this context, both sludge and solid residue may be considered a revalorisable by-product of environmental relevance, provided that stabilization, traceability and monitoring strategies are implemented prior to agronomic use.

## 4. Conclusions

This study provides one of the first integrated mappings of phenolic and metallic composition in olive mill wastewater (OMW) and sludge from abandoned evaporation ponds in Spain, establishing a standardized and reproducible extraction workflow applicable across matrix types. The method demonstrated that OMW retains a substantial reservoir of bioactive phenolics, regardless of its physical state, with recoveries reaching 16 mg GAE/mL in methanolic extracts. LC–MS/MS fingerprinting confirmed >30 phenolic species, including hydroxytyrosol, tyrosol, oleuropein/ligustroside derivatives, quercetin, and anthocyanins, evidencing a chemically diverse and functionally valuable pool for targeted fractionation and formulation.

Matrix state and site-specific factors governed compositional variability: liquid OMWs (TED-4, TED-8) showed higher extractive recoveries, while sludges (TED-13, TED-16) displayed greater phenolic density and mineral accumulation. Importantly, phenolic extracts (FO) exhibited high purity and a favourable toxicological profile, with heavy-metal contents between 0.15 and 21.50 µg/kg, remaining well below EU food safety limits (Reg. 2023/915). Inorganic elements primarily partitioned to the aqueous fraction (AF) or the post-extraction solids, minimizing metal transfer to the phenolic stream. Conversely, fresh sludges concentrated Pb and As (up to 3.78 mg/kg DM and 3.27 mg/kg DM, respectively), though still below thresholds for land application (Royal Decree 1051/2022; Dir. 86/278/EEC). Any agronomic reuse of these solids must nevertheless depend on leaching and mobility assessments to ensure environmental integrity.

Methodologically, the study acknowledges inherent constraints: the Folin–Ciocalteu assay provides an operational estimate of total reducing capacity, LC–MS/MS profiling lacked absolute quantification, and the heterogeneity of pond matrices or the use of conventional rather than fully green extraction may limit direct scalability.

## 5. Patents

Spanish patent application Nº. 202530849: “Preparation of phenolic extracts from alpechin with antifungal activity for fungal control”. Assignee: Universitat de València. S/Ref. 202455R and N/Ref. P-102221.

## Figures and Tables

**Table 1:** Comparative analysis of olive mill wastewater (OMW) characteristics: a cross-regional comparison with existing studies.

TPC ^1^ (g GAE/kg)	Fats and Oils (g/100 g)	Moisture (g/100 g)	pH	State of Matrix	Colour	Area	Reference
3.5 ± 1.9 ^a^	0.2 ± 0.1	96.9 ± 3.9	4.6 ± 0.2	Liquid	Dark Brown to Dark Red	Spain	This study
13.7 ± 9.8 ^b^	5.7 ± 3.3	41.1 ± 5.7	6.1 ± 0.3	Solid	Dark Brown	Spain	
8.65 * (3.8–14.6)	7.4 (4.6–11.9)	64.0 (55.6–74.5)	5.32 (4.86–6.45)	“Alperujo” Dry weight	-	Spain	[48]
5	-	70	4.8	OMW	Black Brown	Greece	[20]
13.2	8.7	-	5.7	“Alperujo”	-	Chile	[49]
6.2	9.8	-	5.1	OMW	-	Italy	[50]
12 **	-	-	4.2	OMW	-	Morocco	[51]
0.002–80 **	0.1–2.3	-	3.0–5.9	OMW	Dark	Mediterranean Area	[52]

^1^ Folin–Ciocalteu method except Kissi et al. 2001 [51]. ^a, b^ Indicates a significant difference in TPC between groups (liquids: n = 8; solids: n = 5), evaluated with Student’s *t*-test (*p* < 0.05). * Calculated from results on dry weight and humidity. ** g/L.

**Table 2:** Determination and classification of main phenolic compounds in analysed olive mill wastewater (OMW) samples (TED-4, TED-6, TED-8, TED-13, TED-15 and TED-16) by chemical structure and source ponds.

Compound	Formula	RT	Liquid	Solid	Group
Malvidin 3-O-rutinoside	C_29_H_35_O_16_	10.36	TED-4, 6	nd	Anthocyanins
Petunidin 3-O-rutinoside	C_28_H_33_O_16_	9.95	TED-4	TED-13	
Cyanidin 3,5-O-diglucoside	C_27_H_31_O_16_	9.14	TED-4, 8	TED-13, 16	
Cyanidin 3-O-(2-xylosyl-galactoside)	C_26_H_29_O_15_	9.11	TED-4	TED-16	
Malvidin 3-O-glucoside	C_23_H_25_O_12_	9.23	TED-4	TED-13, 16	
Petunidin 3-O-galactoside	C_22_H_23_O_12_	8.25	TED-4	TED-13, 16	
Delphinidin 3-O-glucoside	C_21_H_21_O_12_	8.51	TED-4, 6	TED-13, 16	
7-Hydroxysecoisolariciresinol	C_20_H_26_O_7_	6.66	TED-6, 8	TED-13, 15, 16	Lignins
Pinoresinol	C_20_H_22_O_6_	7.02	TED-4	nd	
Sinapic acid	C_11_H_12_O_5_	5.04	TED-4, 6, 8	nd	Phenolic acids
p-Coumaric acid	C_9_H_8_O_3_	4.98	TED-4, 6	TED-13, 15, 16	
Cinnamic acid	C_9_H_8_O_2_	5.18	TED-4,8	TED-16	
Vanilic acid	C_8_H_8_O_4_	1.29	TED-4, 6, 8	TED-13, 15, 16	
Vanillin	C_8_H_8_O_3_	0.64	TED-4, 6, 8	TED-13, 15, 16	
Hydroxybenzoic acid	C_7_H_6_O_3_	2.02	TED-4, 6, 8	TED-13, 15, 16	
Quercetin	C_15_H_10_O_7_	7.68	TED-4, 6, 8	TED-13, 16	Flavonol
Catechin	C_15_H_14_O_6_	4.18	TED-4, 6, 8	TED-13, 15, 16	
Diosmetin	C_16_H_12_O_6_	8.73	TED-4, 8	nd	Flavones and Flavanol
Luteolin	C_15_H_10_O_6_	7.98	TED-4, 6, 8	TED-13, 15, 16	
Apigenin	C_15_H_10_O_5_	8.66	TED-4, 6, 8	TED-15, 16	
Verbascoside	C_29_H_36_O_15_	6.26	TED-4, 8	TED-13, 16	Oleuropein derivatives
Oleuropein	C_25_H_32_O_13_	7.15	TED-4, 6, 8	TED-13, 15, 16	
Ligustroside	C_25_H_32_O_12_	8.63	TED-4, 8	TED-13	
3,4-DHPEA-EA	C_19_H_22_O_8_	5.62	TED-4, 6	nd	
p-HPEA-EA	C_19_H_22_O_7_	6.92	TED-6	TED-13, 16	
Oleoside 11-methylester	C_17_H_24_O_11_	5.69	TED-6, 8	TED-16	
3,4-DHPEA-EDA	C_17_H_20_O_6_	5.37	TED-6, 8	TED-13, 15	
3,4-DHPEA-AC	C_10_H_12_O_4_	6.32	TED-4, 6, 8	TED-13, 15	
p-HPEA-AC	C_10_H_12_O_3_	7.07	TED-4, 6, 8	TED-15, 16	
Tyrosol	C_8_H_10_O_2_	6.30	TED-4, 6, 8	TED-13, 15, 16	
Hydroxytyrosol	C_8_H_10_O_3_	1.28	TED-4, 6, 8	TED-13, 15, 16	

## Data Availability

The data presented in this study are partly included in the article and the Supplementary Materials. Further data can be obtained from the corresponding author upon reasonable request. Certain datasets are temporarily unavailable in order to protect the patentability of the invention (in terms of novelty and inventive step), pending a potential PCT filing.

## Supplementary Materials

The following supporting information can be downloaded at: https://www.mdpi.com/article/10.3390/antiox14111371/s1, Figure S1: Illustration of the location of the abandoned olive mill wastewater (OMW) ponds sampled. Figure S2: (A) Total phenolic content (TPC) in fresh OMW samples, expressed as g gallic acid equivalents (GAE) per kg of fresh matter (FM), according to matrix state: liquid (n = 24), solid (n = 15), and total (n = 39). Different lowercase letters indicate statistically significant differences among groups (one-way ANOVA followed by Tukey’s post hoc test, *p* < 0.05). (B) Percentage contribution of each fraction to the total TPC in OMW samples according to matrix state. Figure S3: Characterization of the defatting process. (A) Percentage loss of TPC (%TPC) during defatting of OMW (n = 3). (B) TPC recovered in the defatting extract (g GAE/kg FM; n = 3). Statistical differences were assessed by one-way ANOVA followed by Tukey’s post hoc test (*p* < 0.05). Figure S4: Average mineral content in the aqueous phase of OMW after conventional treatment, expressed as a percentage of the initial matrix and categorized by matrix state (including pie-chart summary). Figure S5. Representative chromatograms and HPLC-TOF/MS/MS spectra of the main phenolic compounds identified in OMW samples (TED-4, TED-6, TED-8, TED-13, TED-15, and TED-16). Table S1. Traceability of OMW and sludge samples (TED codes), including sampling date, location, matrix type, storage conditions, and time elapsed before analysis. Table S2. LC–MS/MS identification criteria for phenolic compounds in OMW and OMW sludge extracts (TripleTOF 6600+, AB Sciex). Table S3. Complete dataset of mineral content (mg/kg) and heavy metal content (µg/kg) in untreated fresh olive mill wastewater (OMW) samples (TED-4, TED-8, TED-13, and TED-16), expressed as mean ± SD; CV%.
