# Supplementary material for: Phenolic Fingerprints of Spanish Olive Mill Wastewaters (Alpechin): A Step Toward Regional Valorization Through Antioxidant Recovery
Source: Antioxidants (Basel). 2025 Nov 18;14(11):1371. doi: 10.3390/antiox14111371 (PMC12649255; doi:10.3390/antiox14111371)
Supplement: Supplementary file 1 [file antioxidants-14-01371-s001.zip › antioxidants-3934475-supplementary.pdf]

Phenolic Fingerprints of Spanish Olive Mill Wastewaters (Alpechin): A Step Toward Regional Valorization Through Antioxidant Recovery

Sergio Martínez-Terol[1], Emilia Ferrer[1], Pedro V. Martínez-Culebras[1]*, Houda Berrada[1], Noelia Pallarés[1], Jose Saez-Tovar[2], Luciano Orden[2], María R Martínez-Gallardo[3], Ana J. Toribio[3], Francisco J. Barba[1]

[1] Research group in Innovative Technologies for Sustainable Food (ALISOST), Department of Preventive Medi-cine and Public Health, Food Science, Toxicology and Forensic Medicine, Faculty of Pharmacy, Universitat de València, Avenida Vicent Andrés Estellés s/n, 46100, Burjassot, València Spain; sergio.martinez-terol@uv.es (S.M.-T.); emilia.ferrer@uv.es (E.F.); houda.berrada@uv.es (H.B.); noelia.pallares@uv.es (N.P.); francisco.barba@uv.es (F.J.B.)

[2] Instituto de Investigación e Innovación Agroalimentaria y Agroambiental (CIAGRO-UMH), Universidad Miguel Hernández, Carretera de Beniel Km 3.2, Orihuela, Alicante 03312, Spain; jose.saezt@umh.es (J.S.-T.); l.orden@umh.es (L.O.)

[3] Department of Biology and Geology, CITE II-B, University of Almería, Agrifood Campus of International Excellence, ceiA3, CIAIMBITAL, 04120, Almería, Spain; mmg113@ual.es (M.R.M-G); atoribio@ual.es (A.J.T)

* Correspondence: pedro.martinez@uv.es

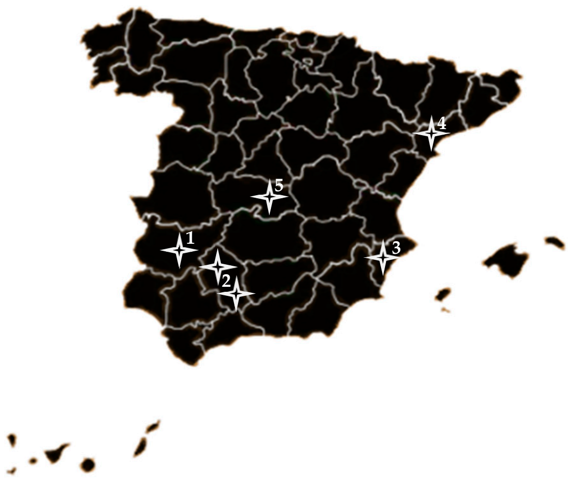

1.	*Badajoz*	TED-2, 3
2.	*Córdoba*	TED-4, 5, 6, 7, 8, 9
3.	*Alicante*	TED-12, 13, 14
4.	*Tarragona*	TED-15
5.	*Toledo*	TED-16

Figure S1. Illustration of the location of the abandoned olive mill wastewater (OMW) ponds subject to sampling.

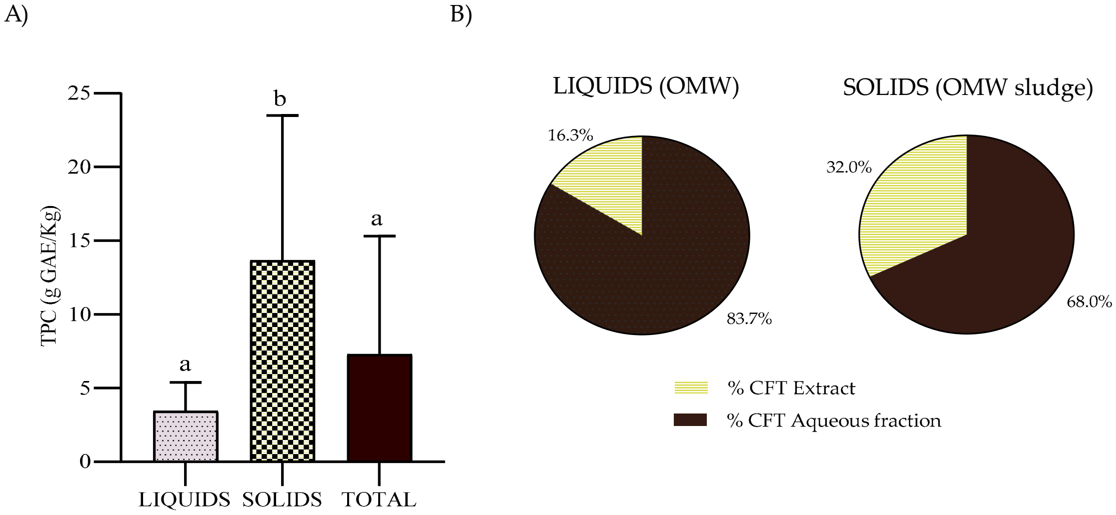

Figure S2. (A) Total phenolic content (TPC) values (g gallic acid equivalents, GAE /kg fresh olive mill wastewater, OMW) according to matrix state. Liquid (n=24), solid (n=15), total (n=39). Small letters: indicate significant difference between groups for TPC (one-way ANOVA followed by Tukey's post-hoc, p value < 0.05). (B) Percentage of TPC of each fraction in the OMW samples according to the state of the matrix.

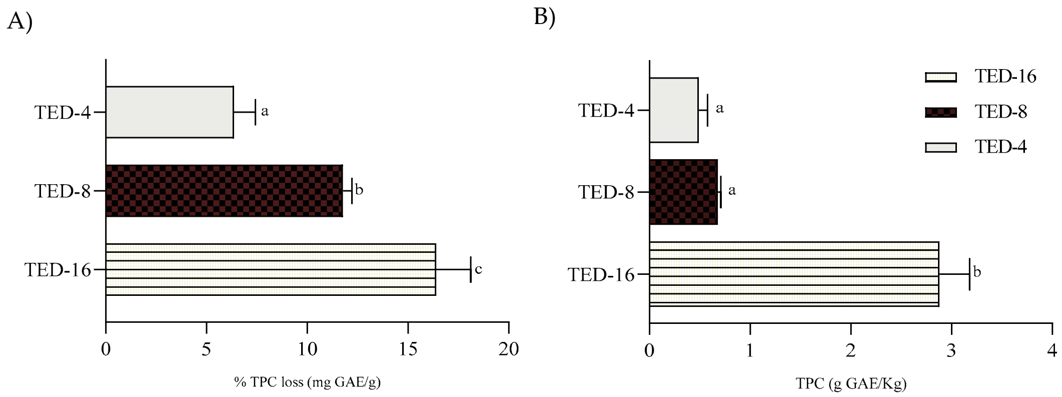

Figure S3. Characterization of the defatting process. A) Percentage loss of total phenolic content (%TPC) during the defatting of olive mill wastewater (OMW) (n=3). B) Total phenolic content recovered in the defatting extract (g gallic acid equivalents, GAE/kg FM; n=3). Statistical differences were determined by one-way ANOVA followed by Tukey's post-hoc test ($p < 0.05$)

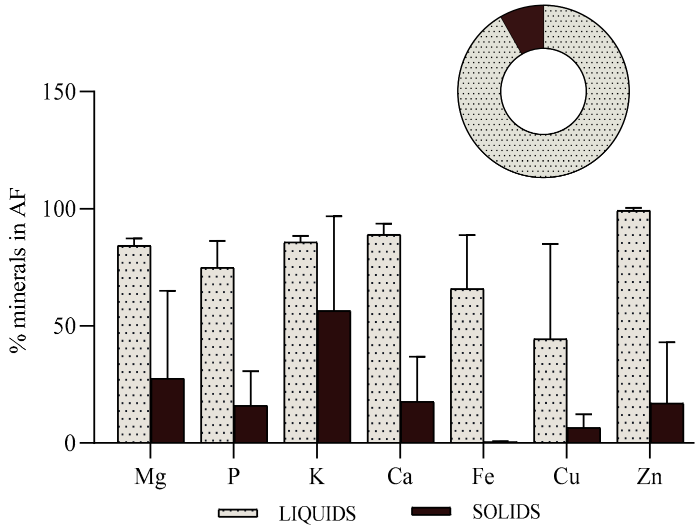

Figure S4. Average mineral content in the aqueous phase of the olive mill wastewater (OMW) after conventional treatment, presented as a percentage of the initial matrix and categorized by state of the matrix. Pie chart: Representation of the average percentage of minerals in the aqueous phase after conventional treatment, classified according to the state of the matrix.

Figure S5. Representative chromatograms and HPLC-TOF/MS/MS spectra of the main phenolic compounds identified in olive mill wastewater (OMW) samples (TED-4, TED-6, TED-8, TED-13, TED-15 and TED-16).

Malvidin 3-O-rutinoside (Mass/RT/Isotope/Library/Formula) ✓ ● ✓ ● ●

Retention Time: 10.36 minutes
Extraction Mass: 638.19
Fit (%) N/A RFit (%) N/A

Exp RT: 0.00 minutes
Analyte Name:
Malvidin 3-O-rutinoside

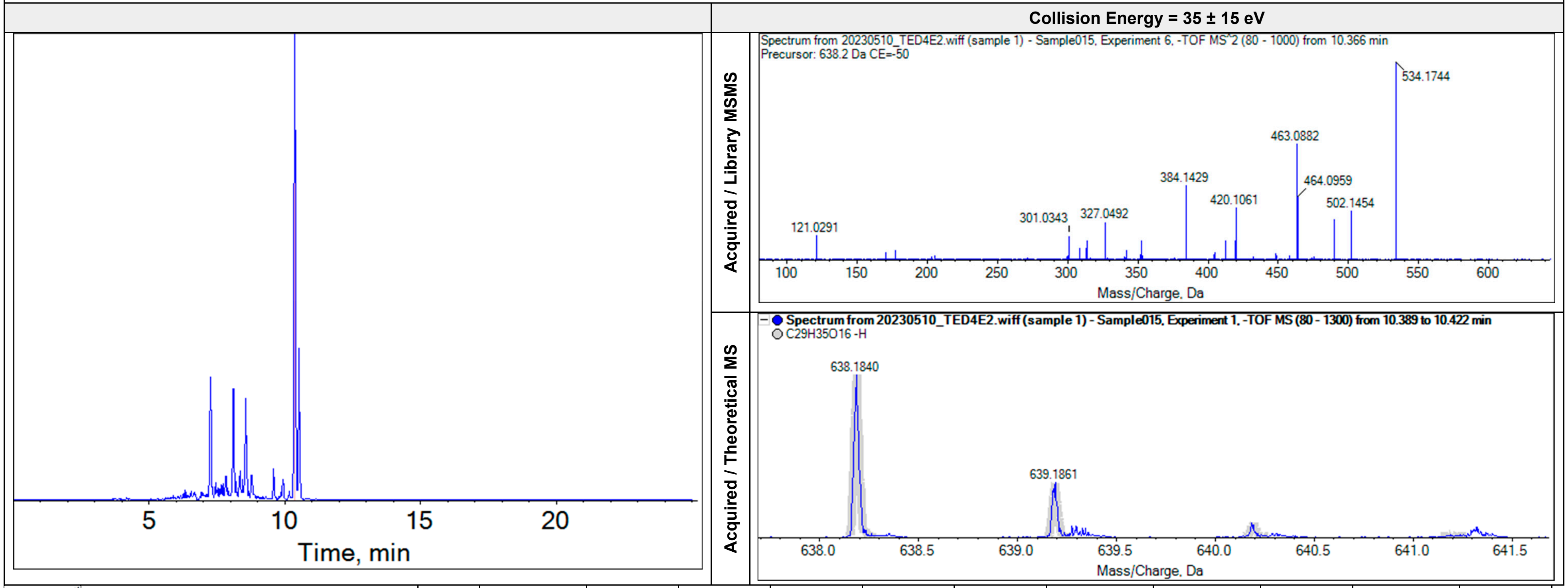

Mass RT Isotope Library Formula	Compound Name (Library Hit)	Score	Formula	Intensity	Threshold	Expected m/z	Found at m/z	Error (ppm)	Expected RT (min)	Found RT (min)	RT Delta (min)	Isotope Diff (%)	Library Score (%)
✓ ● ✓ ● ●	Malvidin 3-O-rutinoside	90%	C29H35O16	467108	50	638.1852	638.1840	-1.9	0.00	10.36	10.36	0.3%	N/A

Petunidin 3-O-rutinoside (Mass/RT/Isotope/Library/Formula) ✓ ● ✓ ● ●

Retention Time: 9.95 minutes<br>Extraction Mass: 624.17<br>Fit (%) N/A RFit (%) N/A	Exp RT: 0.00 minutes<br>Analyte Name:<br>Petunidin 3-O-rutinoside

Collision Energy = 35 ± 15 eV

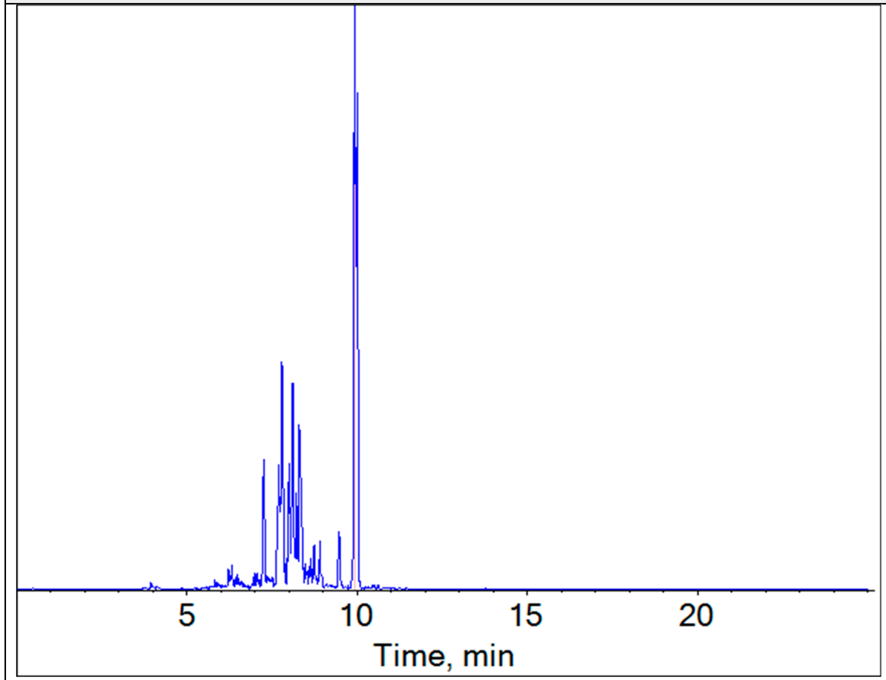

Acquired / Library MSMS

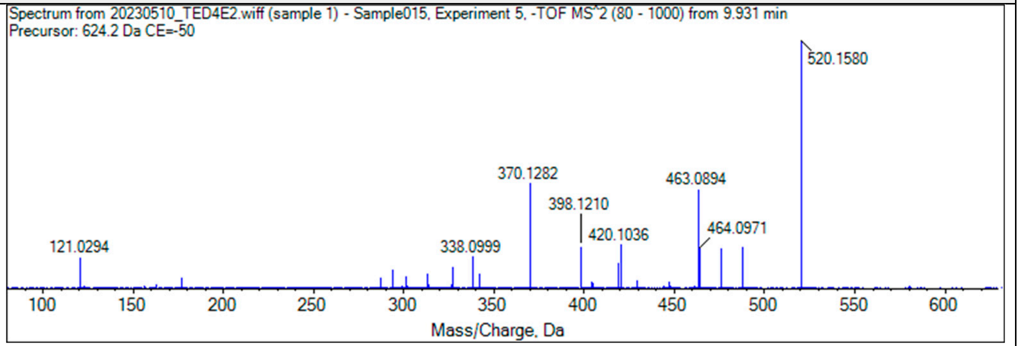

Acquired / Theoretical MS

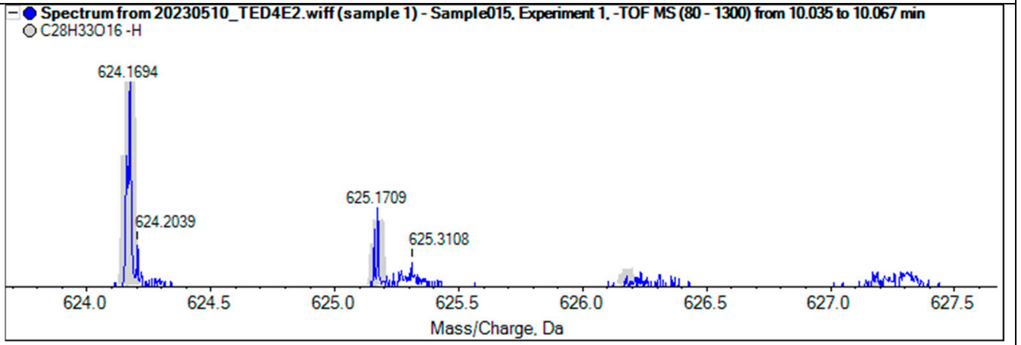

Mass RT Isotope Library Formula ✓✓✓✓✓	Compound Name (Library Hit)	Score	Formula	Intensity	Threshold	Expected m/z	Found at m/z	Error (ppm)	Expected RT (min)	Found RT (min)	RT Delta (min)	Isotope Diff (%)	Library Score (%)
✓ ● ✓ ● ●	Petunidin 3-O-rutinoside	83%	C28H33O16	306308	50	624.1696	624.1694	-0.3	0.00	9.95	9.95	6.3%	N/A

Cyanidin 3,5-O-diglucoside (Mass/RT/Isotope/Library/Formula) ✓ ● ✓ ● ●

Retention Time: 9.14 minutes<br>Extraction Mass: 610.15<br>Fit (%) N/A RFit (%) N/A	Exp RT: 0.00 minutes<br>Analyte Name:<br>Cyanidin 3,5-O-diglucoside

Collision Energy = 35 ± 15 eV

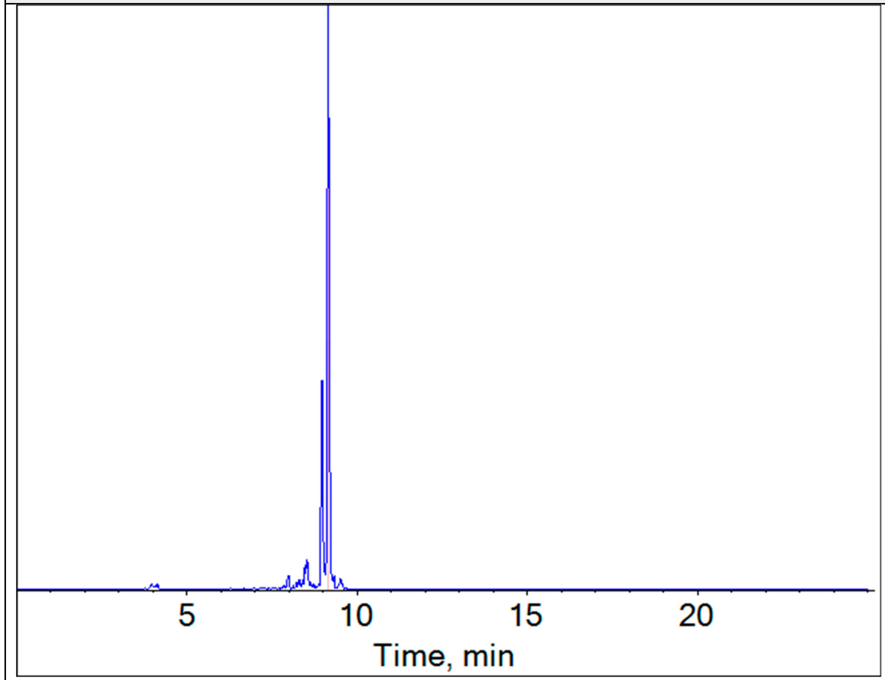

Acquired / Library MSMS

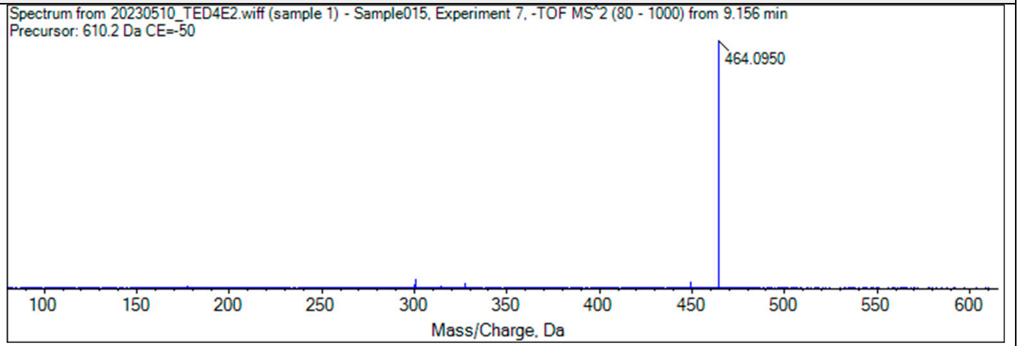

Acquired / Theoretical MS

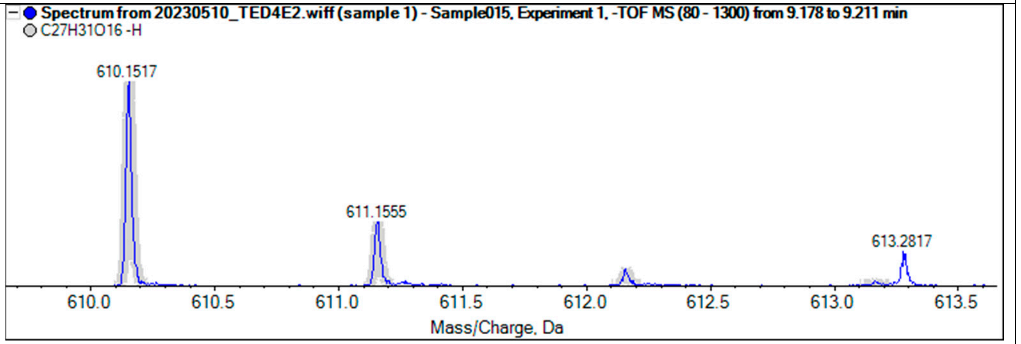

Mass RT Isotope Library Formula ✓✓✓✓✓	Compound Name (Library Hit)	Score	Formula	Intensity	Threshold	Expected m/z	Found at m/z	Error (ppm)	Expected RT (min)	Found RT (min)	RT Delta (min)	Isotope Diff (%)	Library Score (%)
✓ ● ✓ ● ●	Cyanidin 3,5-O-diglucoside	81%	C27H31O16	2343053	50	610.1539	610.1517	-3.7	0.00	9.14	9.14	0.4%	N/A

Cyanidin 3-O-(2-xylosyl-galactoside) (Mass/RT/Isotope/Library/Formula)

Retention Time: 9.11 minutes<br>Extraction Mass: 580.14<br>Fit (%) N/A RFit (%) N/A	Exp RT: 0.00 minutes<br>Analyte Name:<br>Cyanidin 3-O-(2-xylosyl-galactoside)

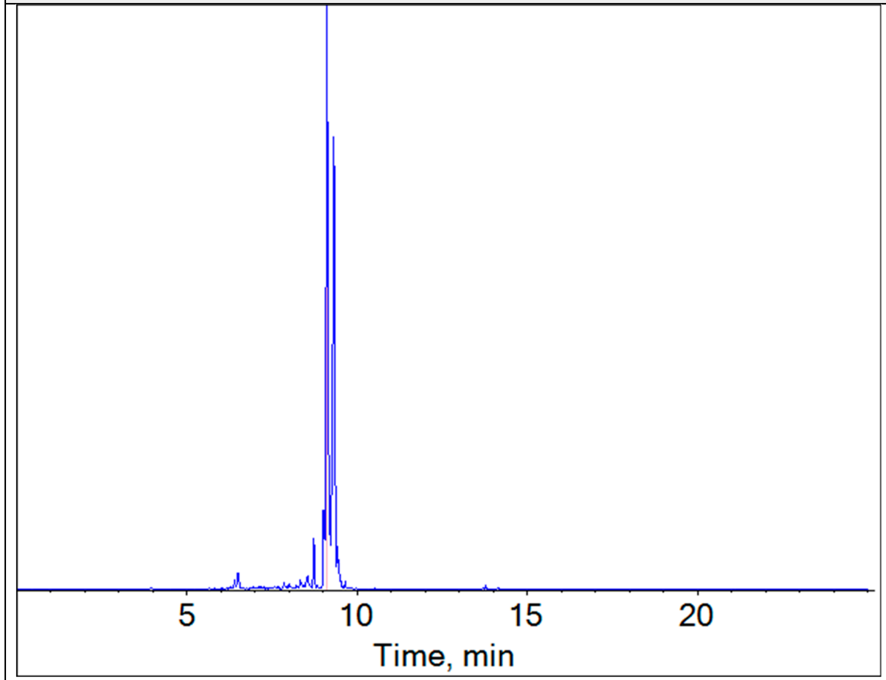

Collision Energy = 35 ± 15 eV

Acquired / Library MSMS

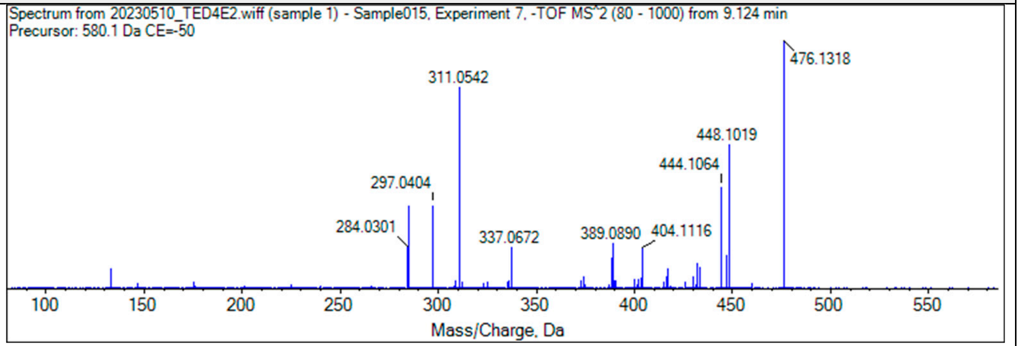

Acquired / Theoretical MS

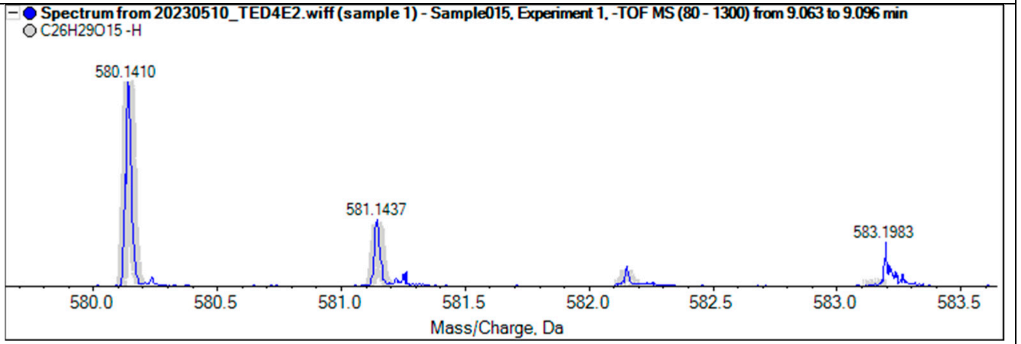

Mass / RT / Isotope / Library / Formula	Compound Name (Library Hit)	Score	Formula	Intensity	Threshold	Expected m/z	Found at m/z	Error (ppm)	Expected RT (min)	Found RT (min)	RT Delta (min)	Isotope Diff (%)	Library Score (%)
✓ ● ✓ ● ●	Cyanidin 3-O-(2-xylosyl-galactoside)	73%	C26H29O15	639469	50	580.1434	580.1410	-4.1	0.00	9.11	9.11	2.5%	N/A

Malvidin 3-O-glucoside (Mass/RT/Isotope/Library/Formula)

Retention Time: 9.23 minutes
Extraction Mass: 492.13
Fit (%) N/A RFit (%) N/A

Exp RT: 0.00 minutes
Analyte Name:
Malvidin 3-O-glucoside

Collision Energy = 35 ± 15 eV

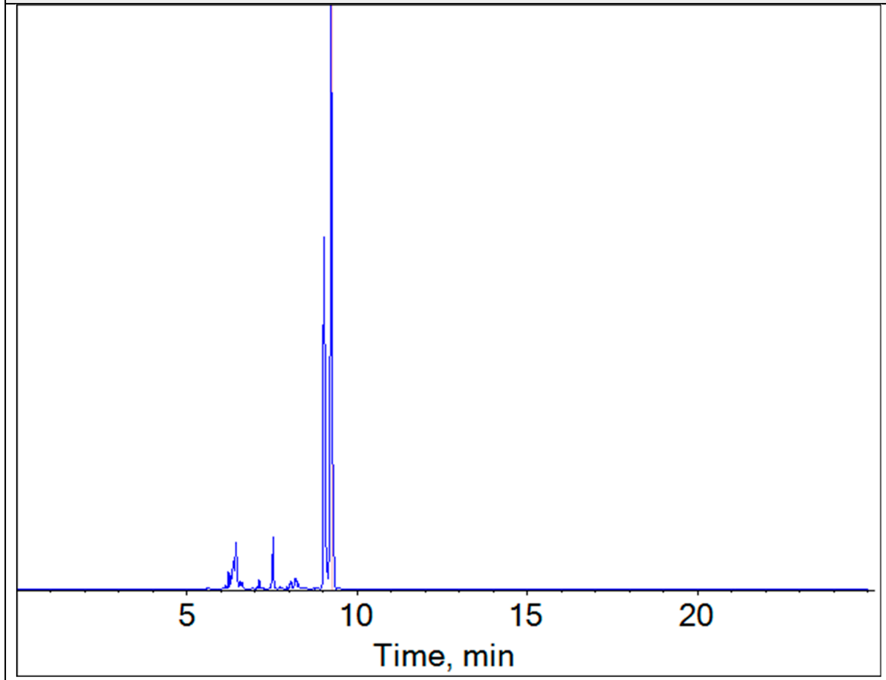

Acquired / Library MSMS

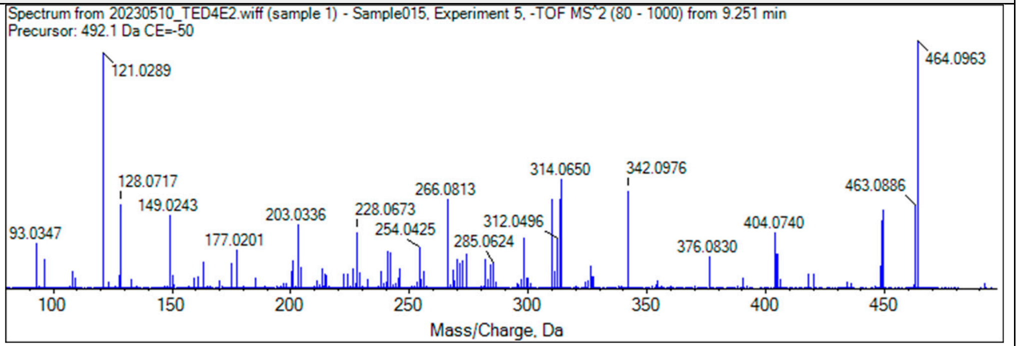

Acquired / Theoretical MS

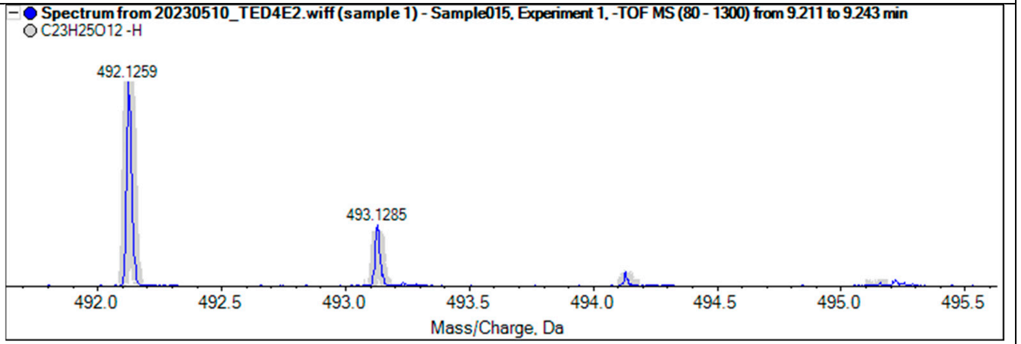

Mass RT Isotope Library Formula	Compound Name (Library Hit)	Score	Formula	Intensity	Threshold	Expected m/z	Found at m/z	Error (ppm)	Expected RT (min)	Found RT (min)	RT Delta (min)	Isotope Diff (%)	Library Score (%)
	Malvidin 3-O-glucoside	78%	C23H25O12	1098923	50	492.1273	492.1259	-2.8	0.00	9.23	9.23	3.2%	N/A

Petunidin 3-O-galactoside (Mass/RT/Isotope/Library/Formula) ✓ ● ✓ ● ●

Retention Time: 8.25 minutes
Extraction Mass: 478.11
Fit (%) N/A RFit (%) N/A

Exp RT: 0.00 minutes
Analyte Name:
Petunidin 3-O-galactoside

Collision Energy = 35 ± 15 eV

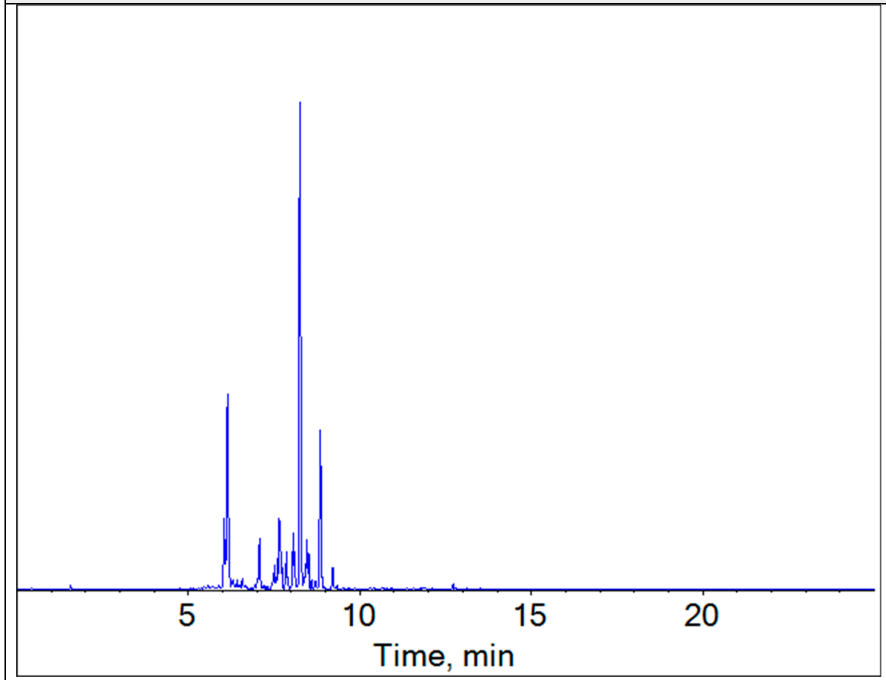

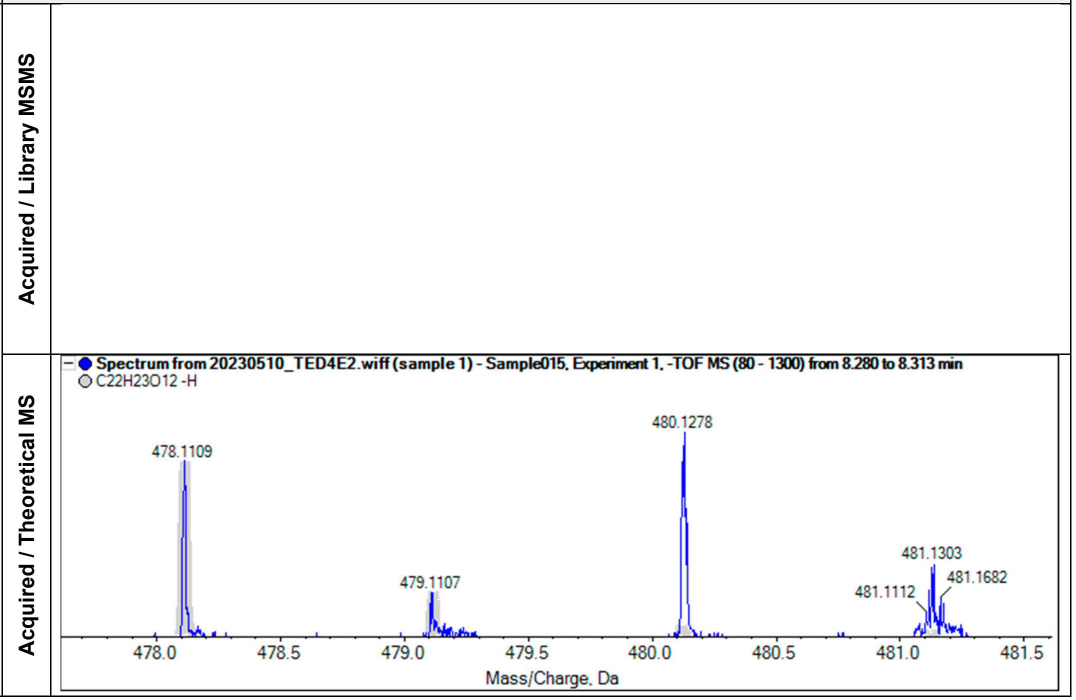

Mass RT Isotope Library Formula	Compound Name (Library Hit)	Score	Formula	Intensity	Threshold	Expected m/z	Found at m/z	Error (ppm)	Expected RT (min)	Found RT (min)	RT Delta (min)	Isotope Diff (%)	Library Score (%)
✓ ● ✓ ● ●	Petunidin 3-O-galactoside	79%	C22H23O12	192234	50	478.1117	478.1109	-1.6	0.00	8.25	8.25	5.4%	N/A

Delphinidin 3-O-glucoside (Mass/RT/Isotope/Library/Formula) ✓ ● ✓ ● ●

Retention Time: 8.51 minutes
Extraction Mass: 464.10
Fit (%) N/A RFit (%) N/A

Exp RT: 0.00 minutes
Analyte Name:
Delphinidin 3-O-glucoside

Collision Energy = 35 ± 15 eV

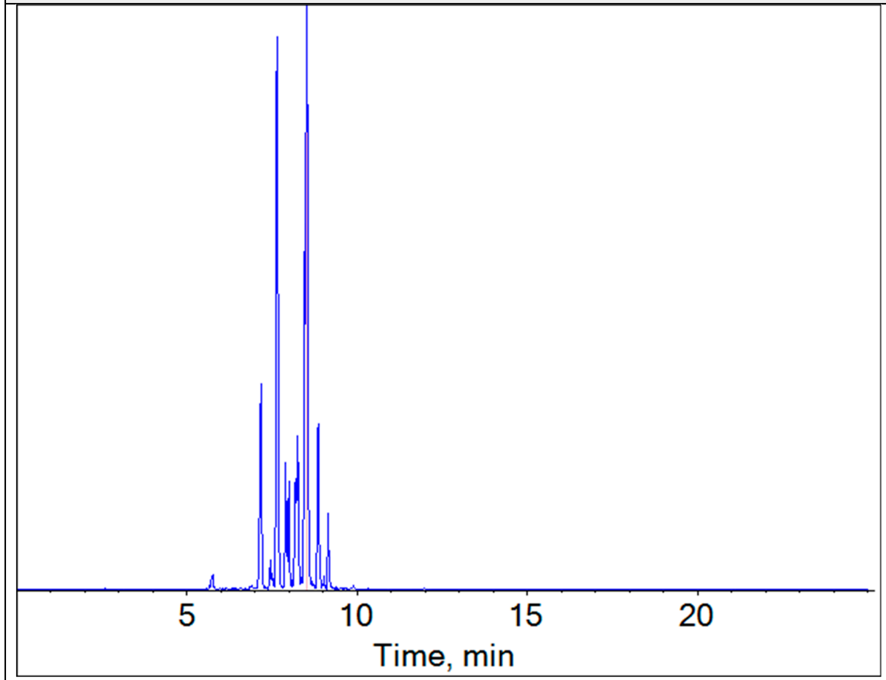

Acquired / Library MSMS

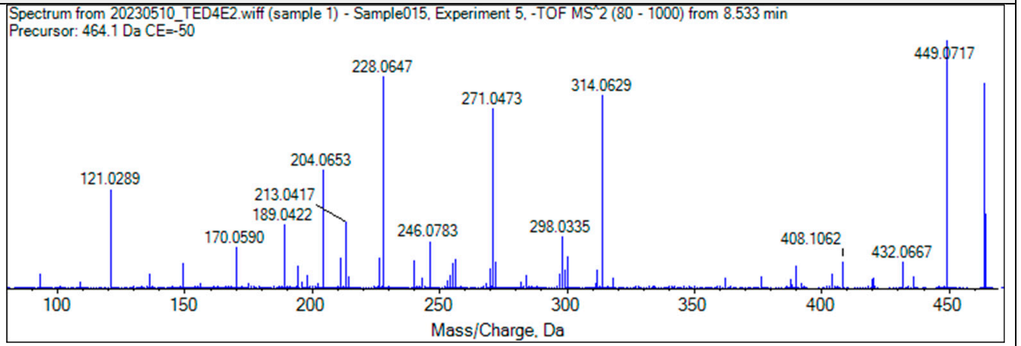

Acquired / Theoretical MS

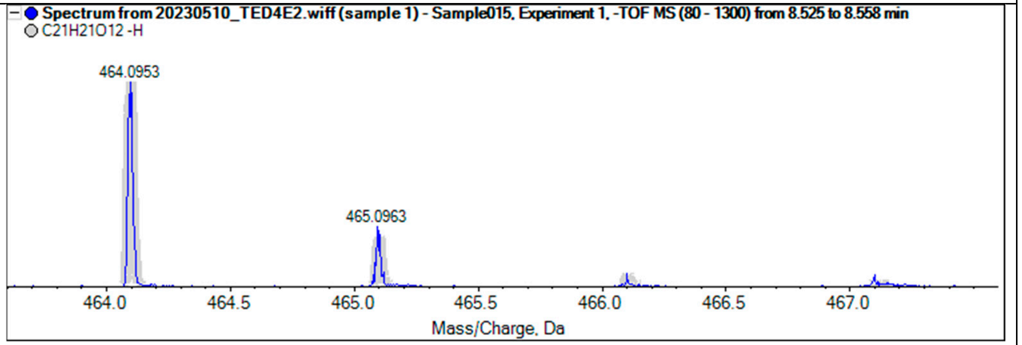

Mass RT Isotope Library Formula ✓✓✓✓✓	Compound Name (Library Hit)	Score	Formula	Intensity	Threshold	Expected m/z	Found at m/z	Error (ppm)	Expected RT (min)	Found RT (min)	RT Delta (min)	Isotope Diff (%)	Library Score (%)
✓ ● ✓ ● ●	Delphinidin 3-O-glucoside	80%	C21H21O12	455104	50	464.0960	464.0953	-1.5	0.00	8.51	8.51	5.0%	N/A

7-Hydroxysecoisolariciresinol (Mass/RT/Isotope/Library/Formula)

Retention Time: 6.66 minutes
Extraction Mass: 377.16
Fit (%) N/A RFit (%) N/A

Exp RT: 0.00 minutes
Analyte Name:
7-Hydroxysecoisolariciresinol

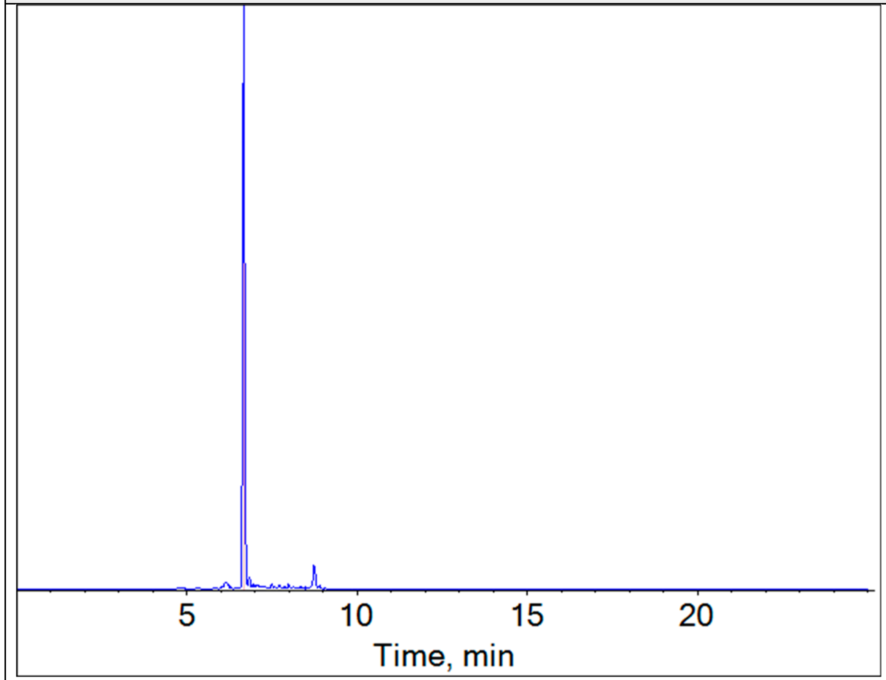

Collision Energy = 35 ± 15 eV

Acquired / Library MSMS

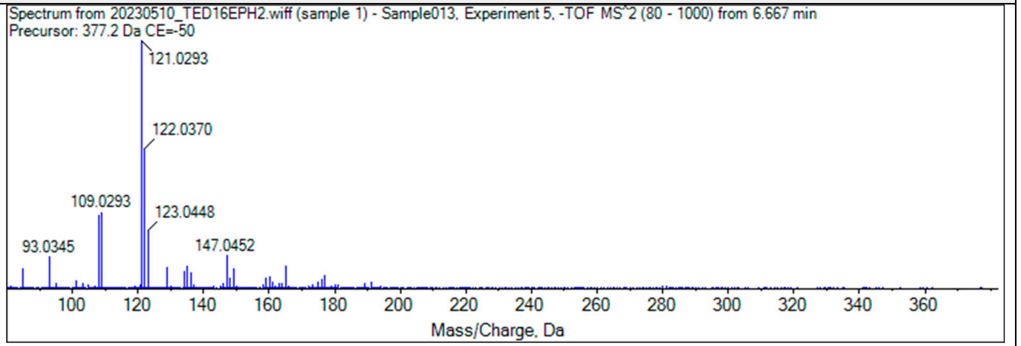

Acquired / Theoretical MS

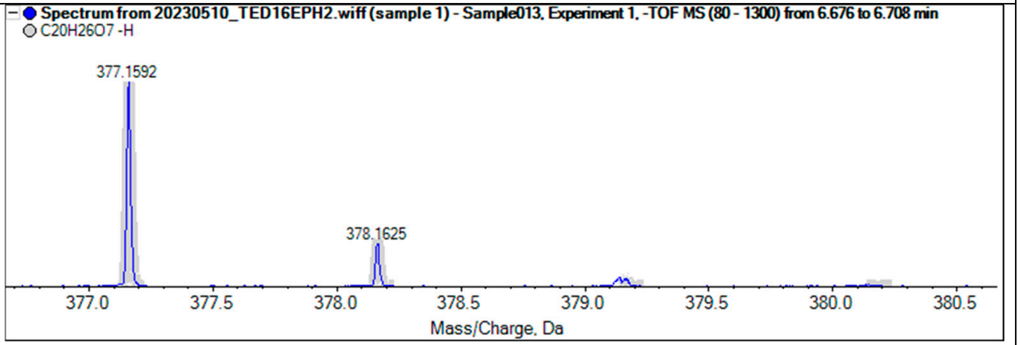

Mass / RT / Isotope / Library / Formula	Compound Name (Library Hit)	Score	Formula	Intensity	Threshold	Expected m/z	Found at m/z	Error (ppm)	Expected RT (min)	Found RT (min)	RT Delta (min)	Isotope Diff (%)	Library Score (%)
✓ ● ✓ ● ●	7-Hydroxysecoisolariciresinol	76%	C20H26O7	4340847	50	377.1606	377.1592	-3.5	0.00	6.66	6.66	2.4%	N/A

Pinoresinol (Mass/RT/Isotope/Library/Formula) ✓ ● ✓ ● ●

Retention Time: 7.02 minutes
Extraction Mass: 357.13
Fit (%) N/A RFit (%) N/A

Exp RT: 0.00 minutes
Analyte Name:
Pinoresinol

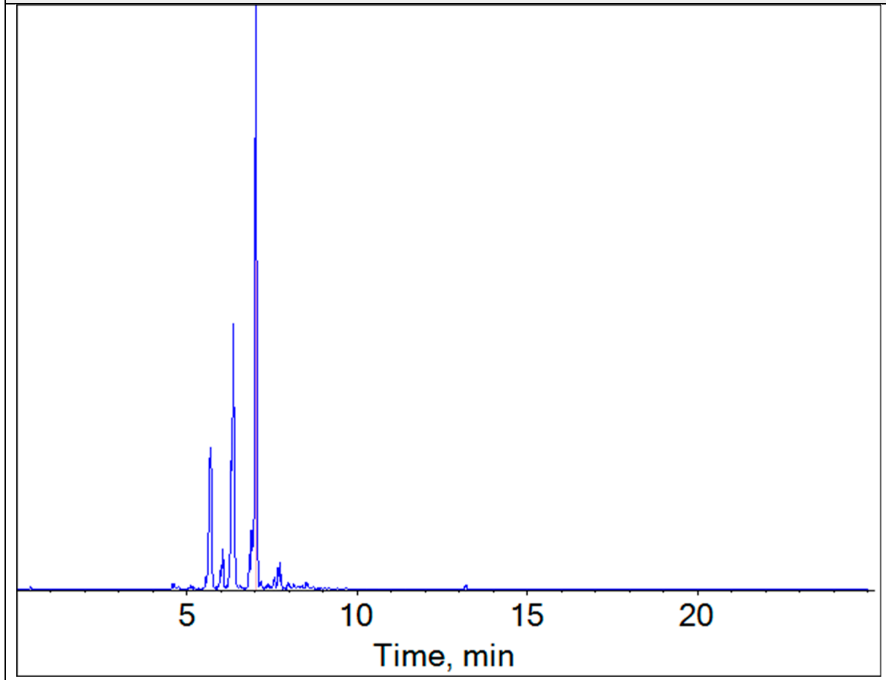

Collision Energy = 35 ± 15 eV

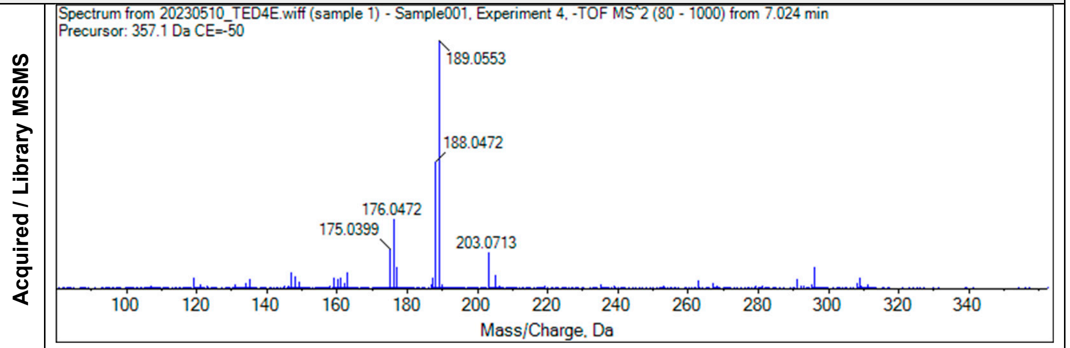

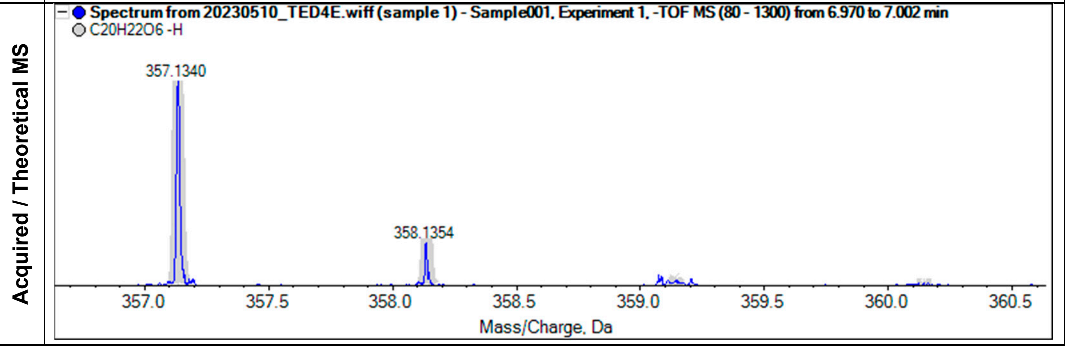

Mass RT Isotope Library Formula ✓✓✓✓✓	Compound Name (Library Hit)	Score	Formula	Intensity	Threshold	Expected m/z	Found at m/z	Error (ppm)	Expected RT (min)	Found RT (min)	RT Delta (min)	Isotope Diff (%)	Library Score (%)
✓ ● ✓ ● ●	Pinoresinol	90%	C20H22O6	573580	50	357.1344	357.1340	-1.0	0.00	7.02	7.02	2.0%	N/A

Sinapic acid (Mass/RT/Isotope/Library/Formula) ✓ ● ✓ ● ●

Retention Time: 5.04 minutes
Extraction Mass: 223.06
Fit (%) N/A RFit (%) N/A

Exp RT: 0.00 minutes
Analyte Name:
Sinapic acid

Collision Energy = 35 ± 15 eV

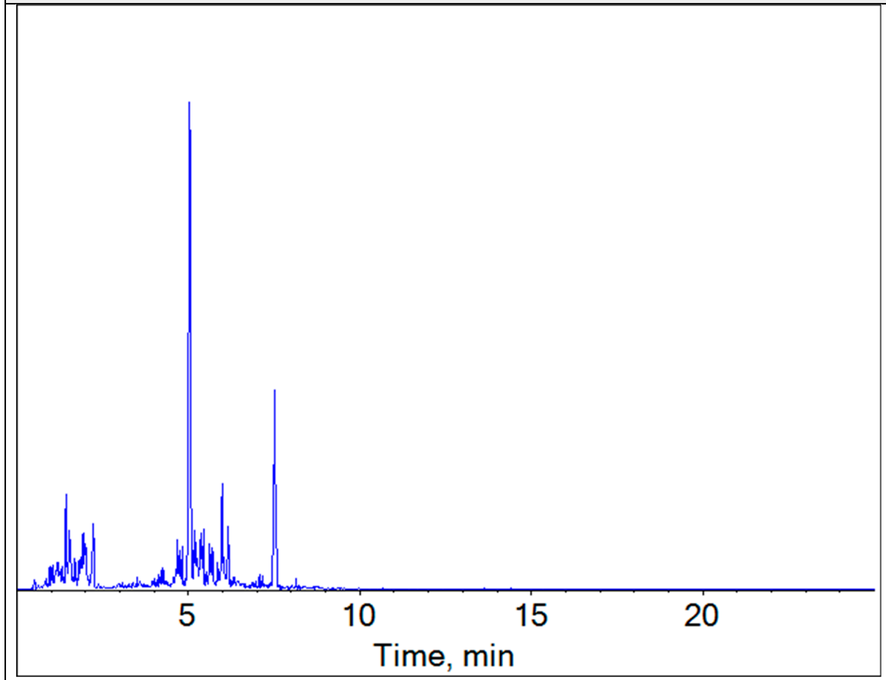

Acquired / Library MSMS

Acquired / Theoretical MS

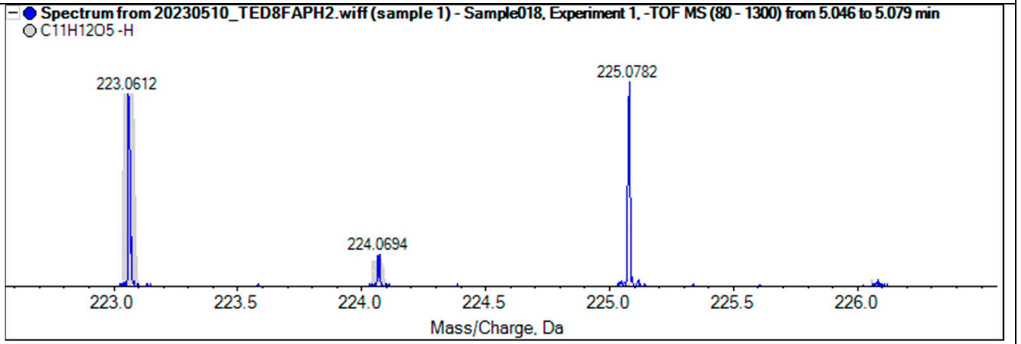

Mass RT Isotope Library Formula ✓✓✓✓✓	Compound Name (Library Hit)	Score	Formula	Intensity	Threshold	Expected m/z	Found at m/z	Error (ppm)	Expected RT (min)	Found RT (min)	RT Delta (min)	Isotope Diff (%)	Library Score (%)
✓●✓●●	Sinapic acid	91%	C11H12O5	72857	50	223.0612	223.0612	0.0	0.00	5.04	5.04	3.4%	N/A

p-Coumaric acid (Mass/RT/Isotope/Library/Formula) ✓ ● ✓ ● ●

Retention Time: 4.98 minutes
Extraction Mass: 163.04
Fit (%) N/A RFit (%) N/A

Exp RT: 0.00 minutes
Analyte Name:
p-Coumaric acid

Collision Energy = 35 ± 15 eV

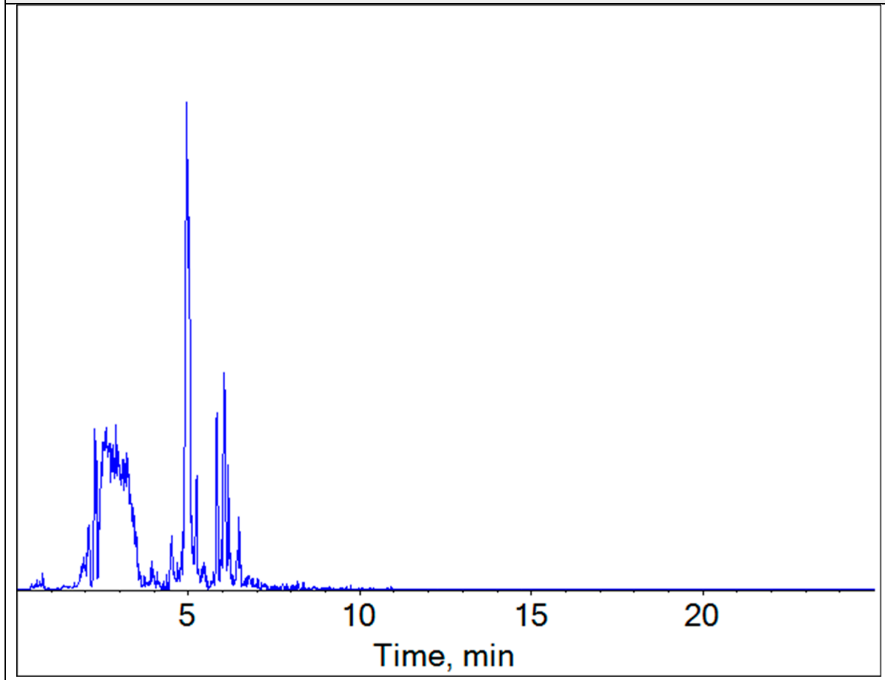

Acquired / Library MSMS

Acquired / Theoretical MS

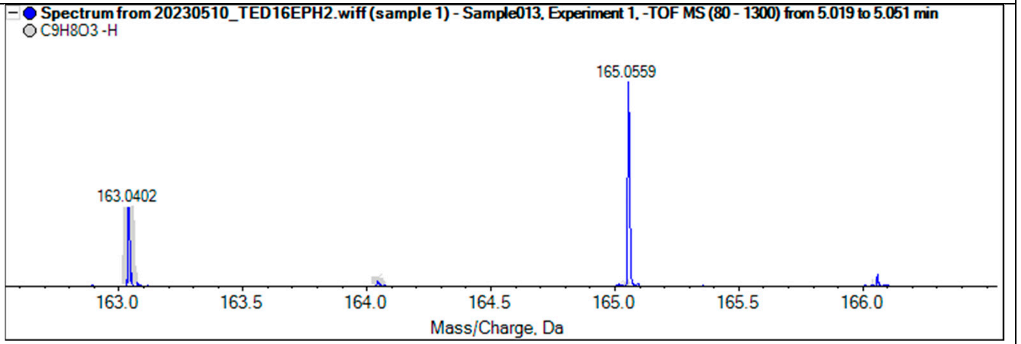

Mass RT Isotope Library Formula ✓✓✓✓✓	Compound Name (Library Hit)	Score	Formula	Intensity	Threshold	Expected m/z	Found at m/z	Error (ppm)	Expected RT (min)	Found RT (min)	RT Delta (min)	Isotope Diff (%)	Library Score (%)
✓ ● ✓ ● ●	p-Coumaric acid	87%	C9H8O3	68024	50	163.0401	163.0402	0.6	0.00	4.98	4.98	4.0%	N/A

Cinnamic acid (Mass/RT/Isotope/Library/Formula)

Retention Time: 5.18 minutes<br>Extraction Mass: 147.05<br>Fit (%) N/A RFit (%) N/A	Exp RT: 0.00 minutes<br>Analyte Name:<br>Cinnamic acid

Collision Energy = 35 ± 15 eV

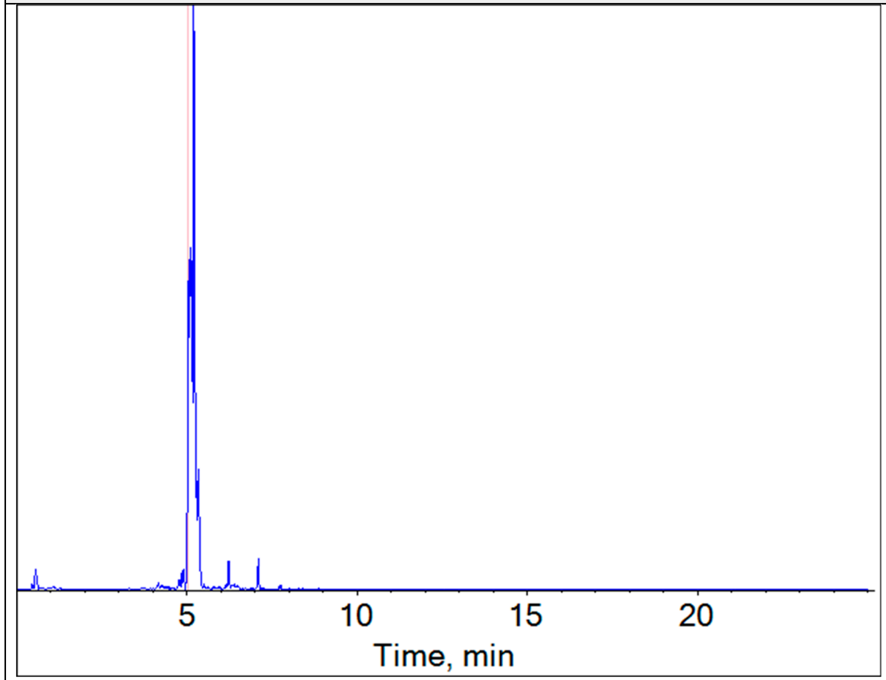

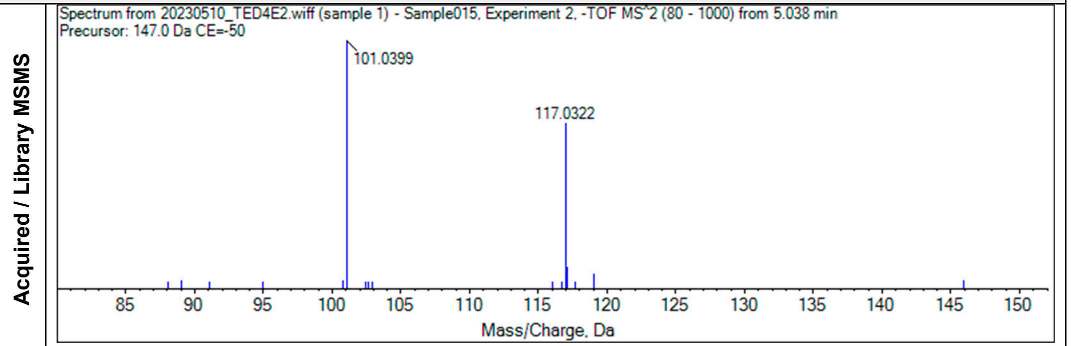

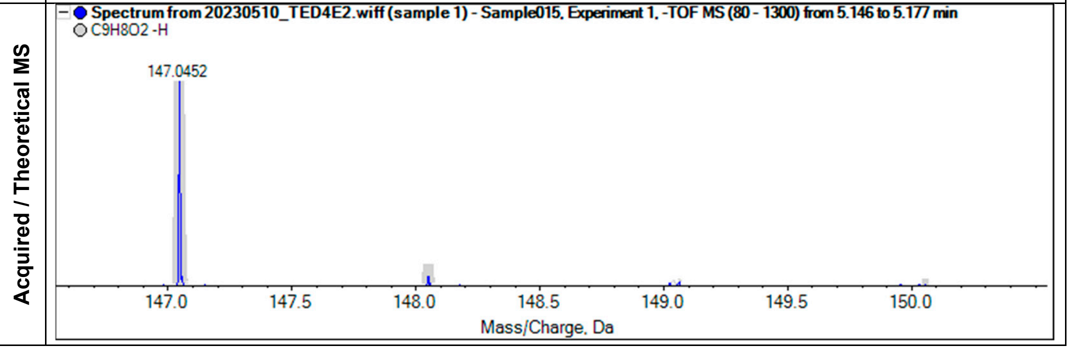

Mass RT Isotope Library Formula	Compound Name (Library Hit)	Score	Formula	Intensity	Threshold	Expected m/z	Found at m/z	Error (ppm)	Expected RT (min)	Found RT (min)	RT Delta (min)	Isotope Diff (%)	Library Score (%)
✓ ● ✓ ● ●	Cinnamic acid	84%	C9H8O2	285661	50	147.0452	147.0452	0.2	0.00	5.18	5.18	5.8%	N/A

Vanillic acid (Mass/RT/Isotope/Library/Formula) ✓ ● ✓ ● ●

Retention Time: 1.29 minutes<br>Extraction Mass: 167.03<br>Fit (%) N/A RFit (%) N/A	Exp RT: 0.00 minutes<br>Analyte Name:<br>Vanillic acid

Collision Energy = 35 ± 15 eV

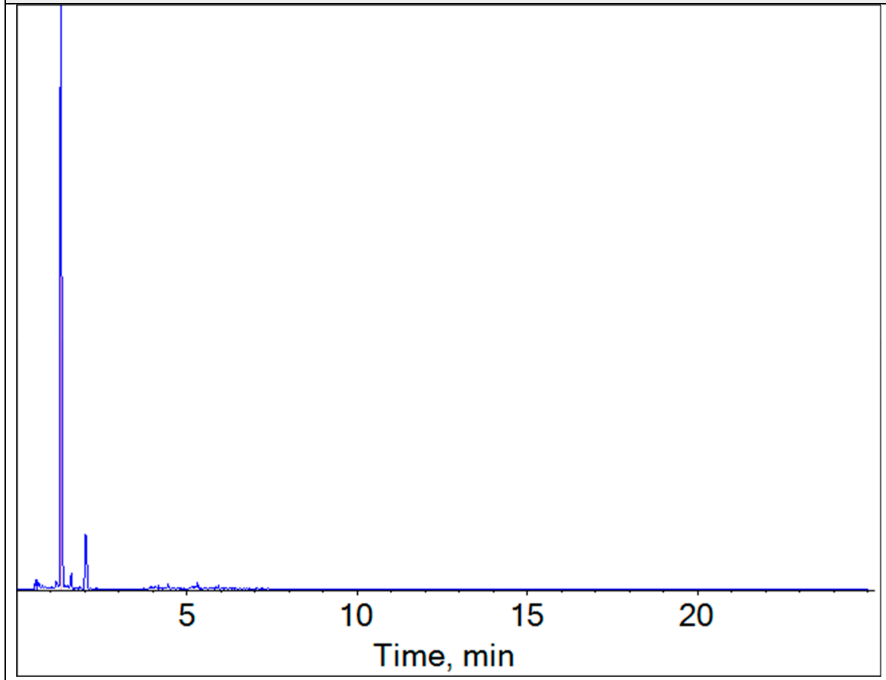

Acquired / Library MSMS

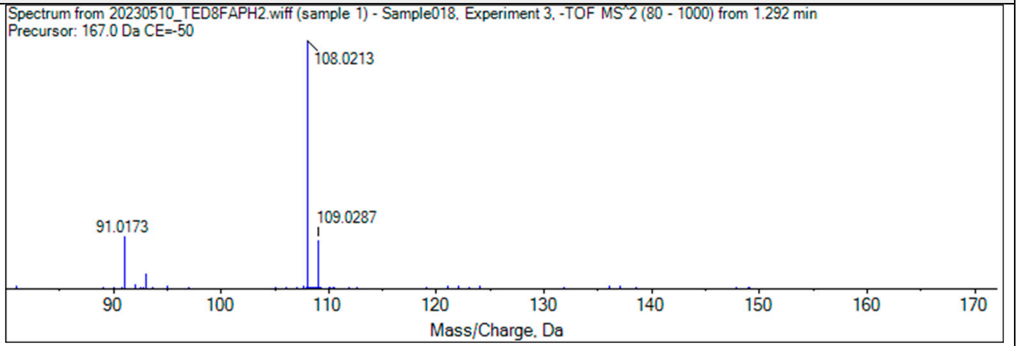

Acquired / Theoretical MS

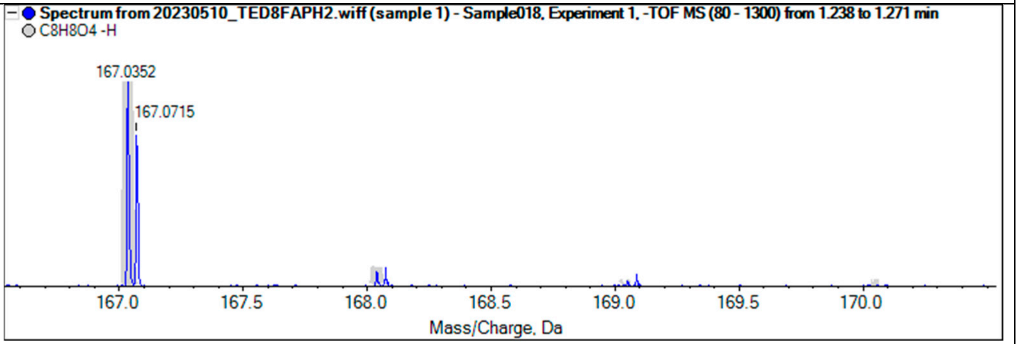

Mass RT Isotope Library Formula ✓✓✓✓✓	Compound Name (Library Hit)	Score	Formula	Intensity	Threshold	Expected m/z	Found at m/z	Error (ppm)	Expected RT (min)	Found RT (min)	RT Delta (min)	Isotope Diff (%)	Library Score (%)
✓ ● ✓ ● ●	Vanillic acid	88%	C8H8O4	285645	50	167.0350	167.0352	1.3	0.00	1.29	1.29	2.1%	N/A

Vanillin (Mass/RT/Isotope/Library/Formula) ✓ ● ✓ ● ●

Retention Time: 0.64 minutes
Extraction Mass: 151.04
Fit (%) N/A RFit (%) N/A

Exp RT: 0.00 minutes
Analyte Name:
Vanillin

Collision Energy = 35 ± 15 eV

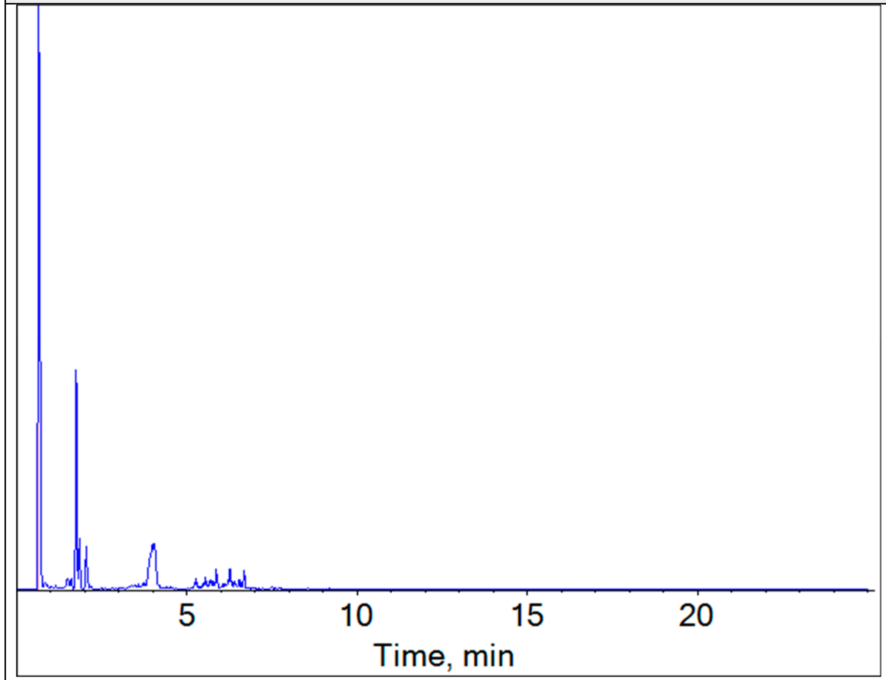

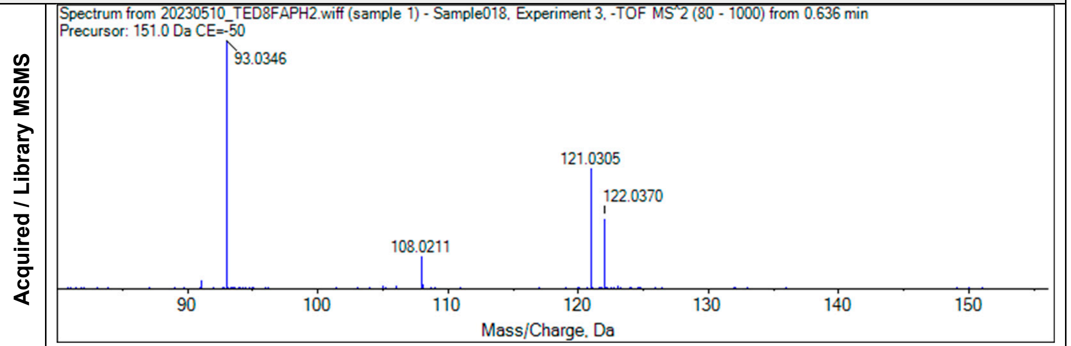

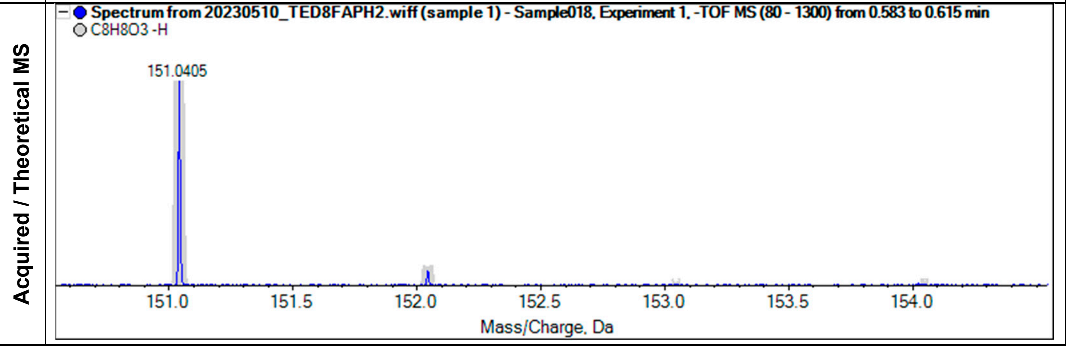

Mass RT Isotope Library Formula ✓✓✓✓✓	Compound Name (Library Hit)	Score	Formula	Intensity	Threshold	Expected m/z	Found at m/z	Error (ppm)	Expected RT (min)	Found RT (min)	RT Delta (min)	Isotope Diff (%)	Library Score (%)
✓ ● ✓ ● ●	Vanillin	80%	C8H8O3	977268	50	151.0401	151.0405	2.8	0.00	0.64	0.64	2.4%	N/A

3-Hydroxybenzoic acid (Mass/RT/Isotope/Library/Formula) ✓ ● ✓ ● ●

Retention Time: 2.02 minutes
Extraction Mass: 137.02
Fit (%) N/A RFit (%) N/A

Exp RT: 0.00 minutes
Analyte Name:
3-Hydroxybenzoic acid

Collision Energy = 35 ± 15 eV

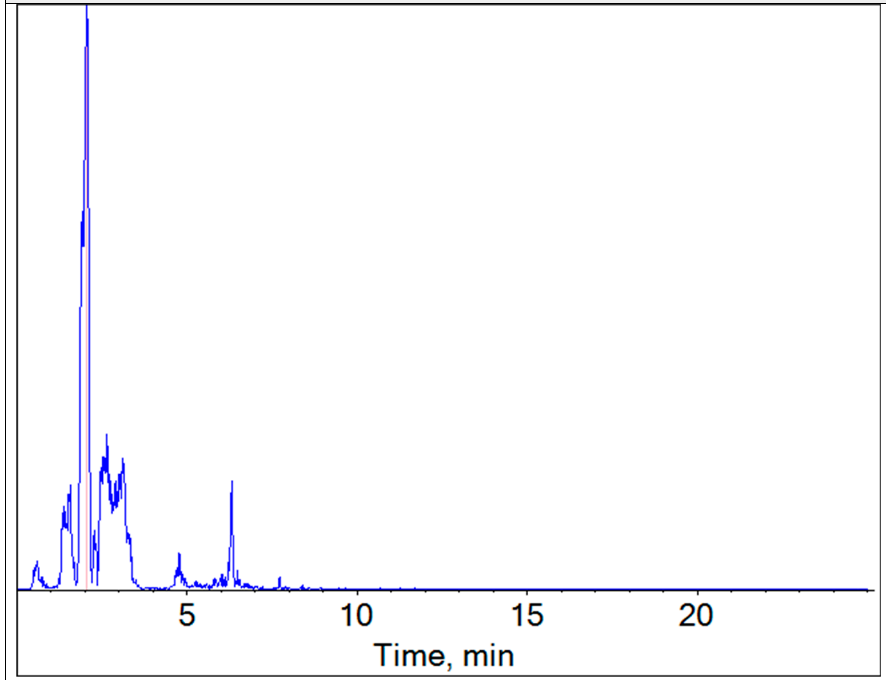

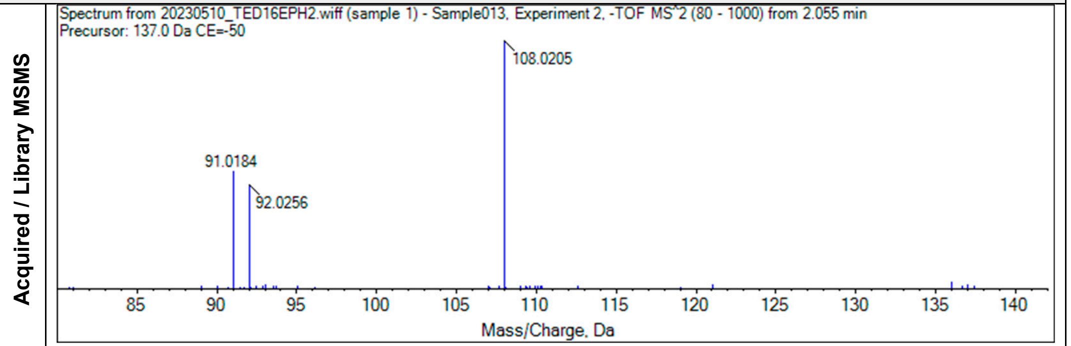

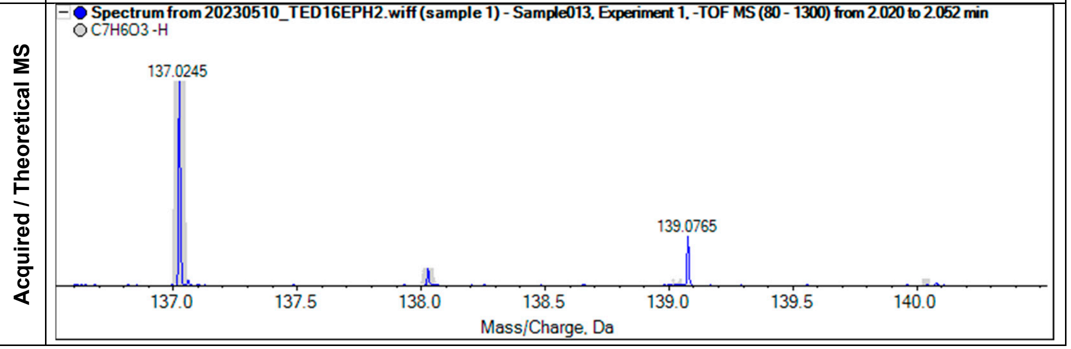

Mass RT Isotope Library Formula	Compound Name (Library Hit)	Score	Formula	Intensity	Threshold	Expected m/z	Found at m/z	Error (ppm)	Expected RT (min)	Found RT (min)	RT Delta (min)	Isotope Diff (%)	Library Score (%)
✓ ● ✓ ● ●	3-Hydroxybenzoic acid	95%	C7H6O3	194336	50	137.0244	137.0245	0.6	0.00	2.02	2.02	0.9%	N/A

Quercetin (Mass/RT/Isotope/Library/Formula) ✓ ● ✓ ● ●

Retention Time: 7.68 minutes
Extraction Mass: 301.04
Fit (%) N/A RFit (%) N/A

Exp RT: 0.00 minutes
Analyte Name:
Quercetin

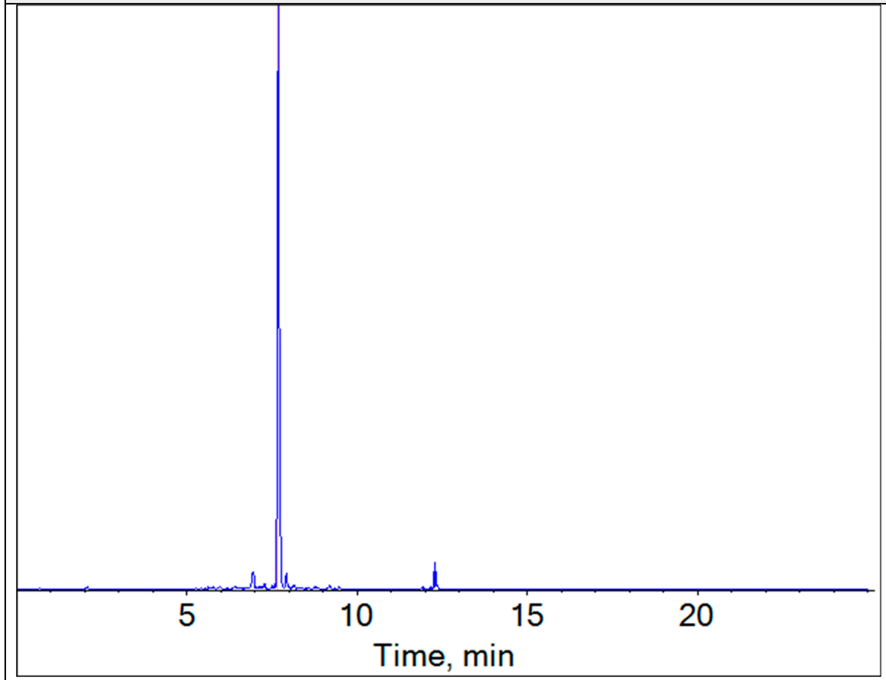

Collision Energy = 35 ± 15 eV

Acquired / Library MSMS

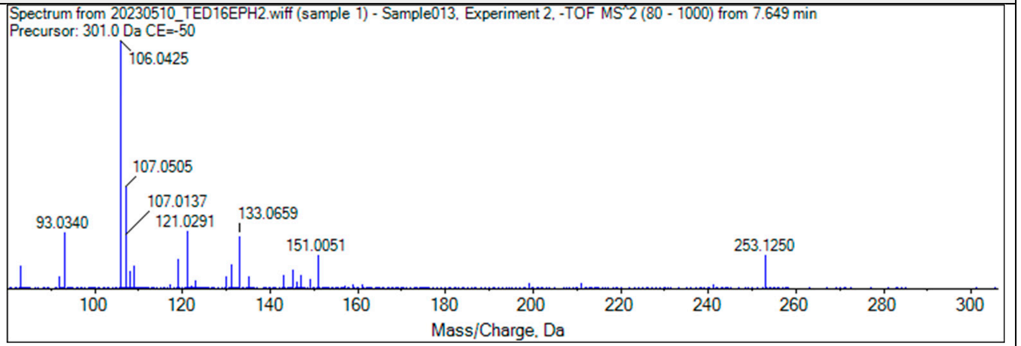

Acquired / Theoretical MS

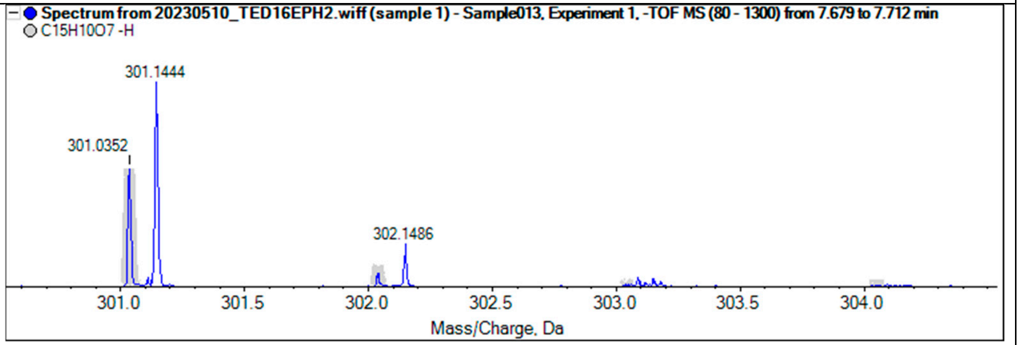

Mass / RT / Isotope / Library / Formula	Compound Name (Library Hit)	Score	Formula	Intensity	Threshold	Expected m/z	Found at m/z	Error (ppm)	Expected RT (min)	Found RT (min)	RT Delta (min)	Isotope Diff (%)	Library Score (%)
✓ ● ✓ ● ●	Quercetin	82%	C15H10O7	414057	50	301.0354	301.0352	-0.7	0.00	7.68	7.68	5.9%	N/A

(-)-Catechin (Mass/RT/Isotope/Library/Formula)

Retention Time: 4.18 minutes<br>Extraction Mass: 289.07<br>Fit (%) N/A RFit (%) N/A	Exp RT: 0.00 minutes<br>Analyte Name:<br>(-)-Catechin

Collision Energy = 35 ± 15 eV

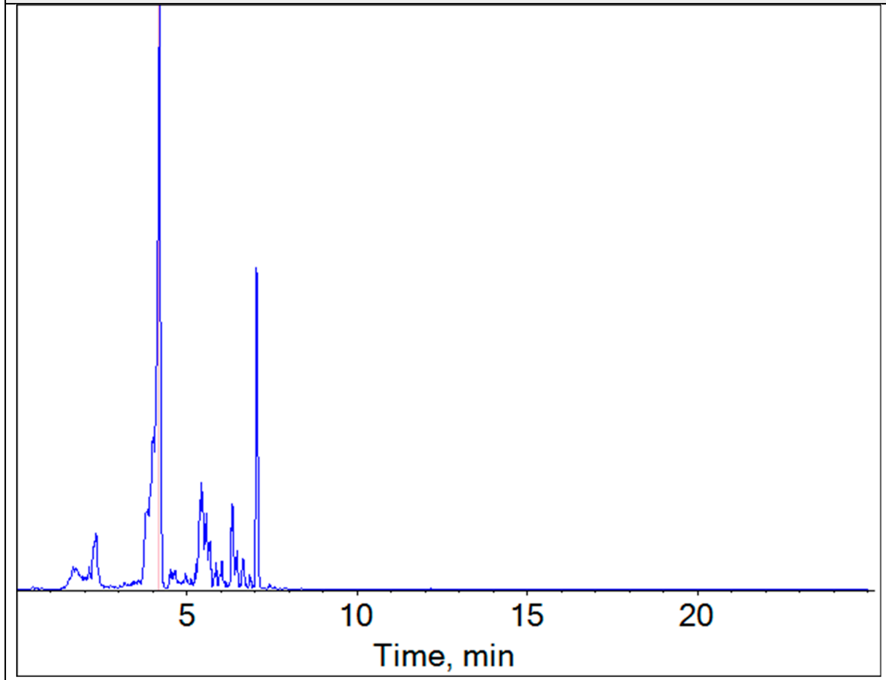

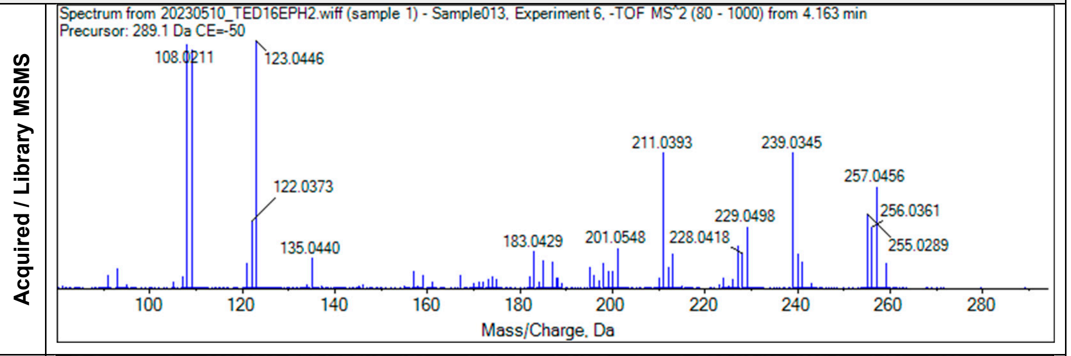

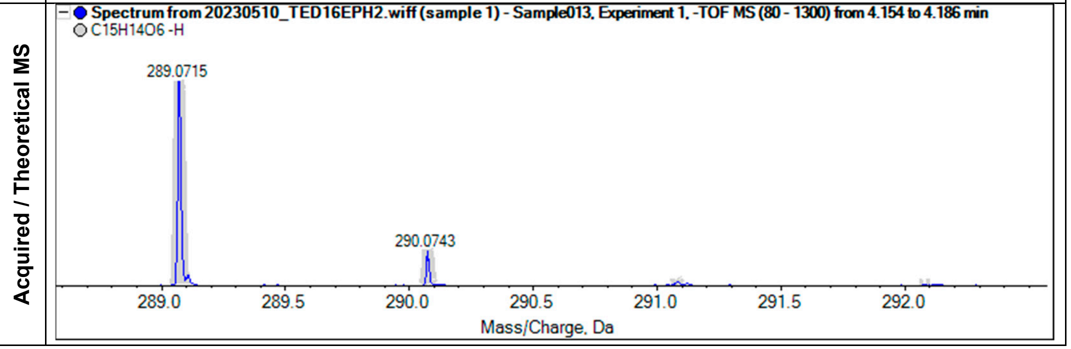

Mass RT Isotope Library Formula	Compound Name (Library Hit)	Score	Formula	Intensity	Threshold	Expected m/z	Found at m/z	Error (ppm)	Expected RT (min)	Found RT (min)	RT Delta (min)	Isotope Diff (%)	Library Score (%)
✓ ● ✓ ● ●	(-)-Catechin	94%	C15H14O6	989173	50	289.0718	289.0715	-0.8	0.00	4.18	4.18	0.8%	N/A

Diosmetin (Mass/RT/Isotope/Library/Formula)

Retention Time: 8.73 minutes
Extraction Mass: 299.06
Fit (%) N/A RFit (%) N/A

Exp RT: 0.00 minutes
Analyte Name:
Diosmetin

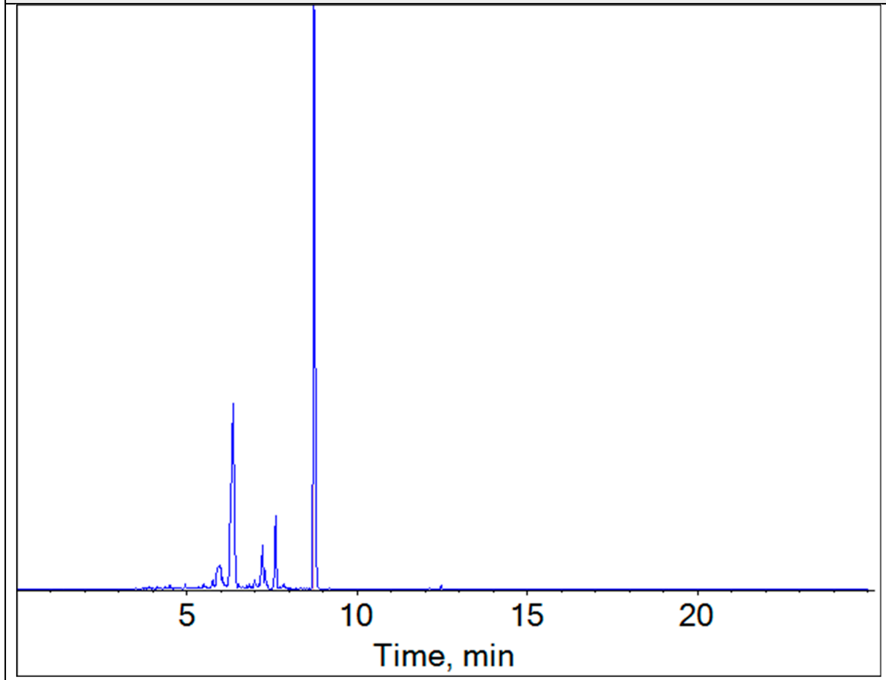

Collision Energy = 35 ± 15 eV

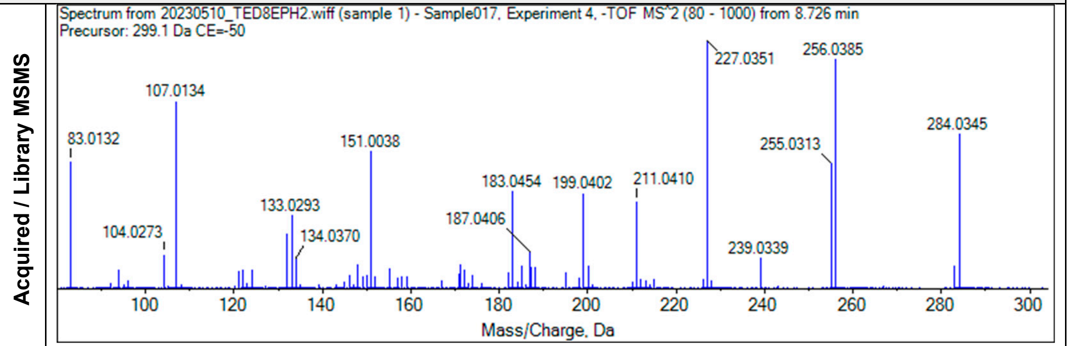

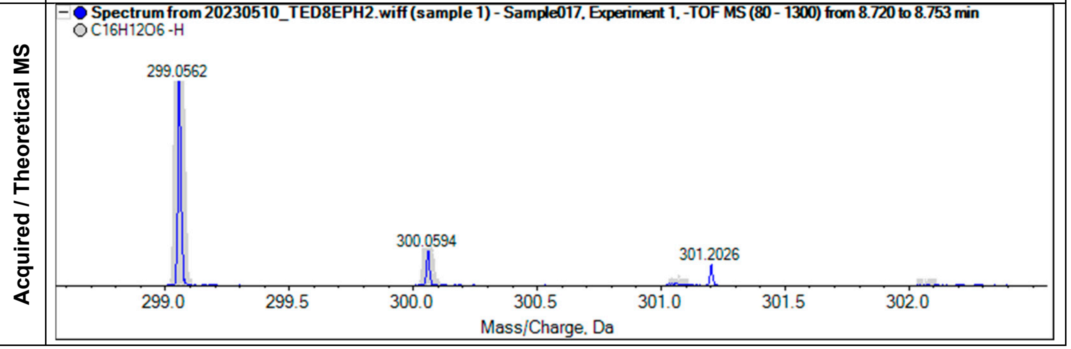

Mass/RT/Isotope/Library/Formula	Compound Name (Library Hit)	Score	Formula	Intensity	Threshold	Expected m/z	Found at m/z	Error (ppm)	Expected RT (min)	Found RT (min)	RT Delta (min)	Isotope Diff (%)	Library Score (%)
✓ ● ✓ ● ●	Diosmetin	95%	C16H12O6	1208223	50	299.0561	299.0562	0.1	0.00	8.73	8.73	1.6%	N/A

Luteolin (Mass/RT/Isotope/Library/Formula)

Retention Time: 7.98 minutes
Extraction Mass: 285.04
Fit (%) N/A RFit (%) N/A

Exp RT: 0.00 minutes
Analyte Name:
Luteolin

Collision Energy = 35 ± 15 eV

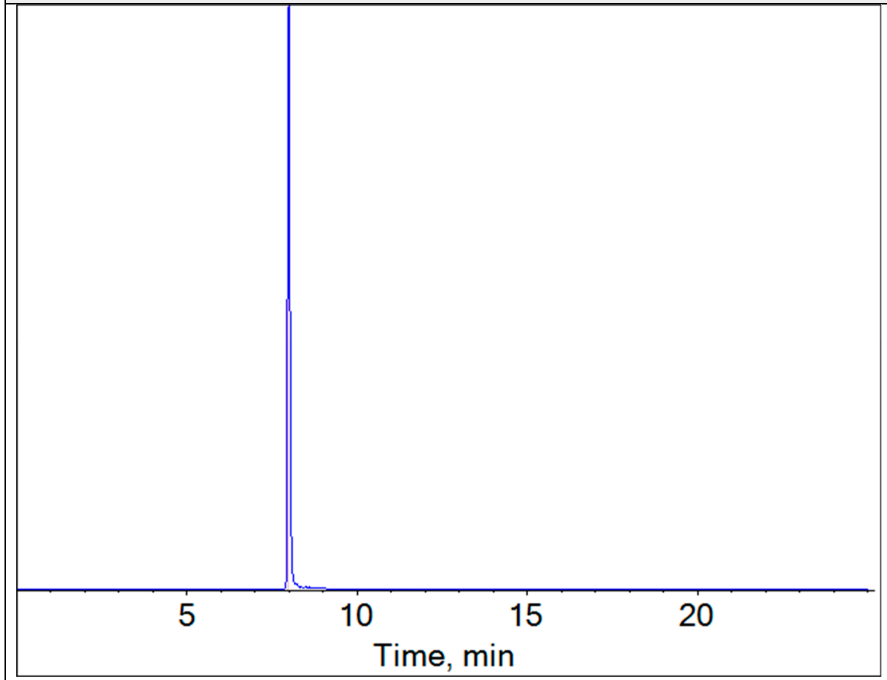

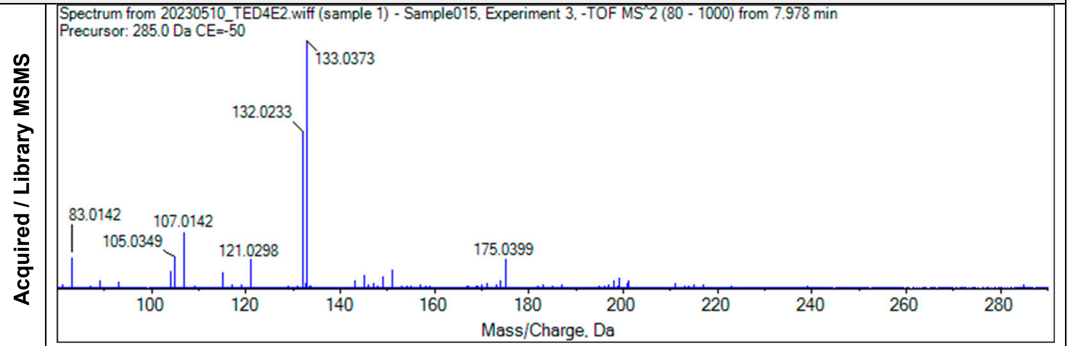

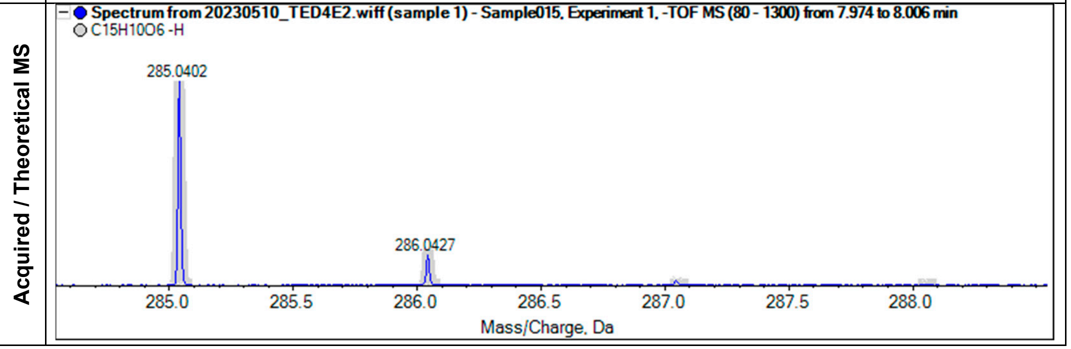

Mass RT Isotope Library Formula	Compound Name (Library Hit)	Score	Formula	Intensity	Threshold	Expected m/z	Found at m/z	Error (ppm)	Expected RT (min)	Found RT (min)	RT Delta (min)	Isotope Diff (%)	Library Score (%)
✓ ● ✓ ● ●	Luteolin	89%	C15H10O6	71705270	50	285.0405	285.0402	-1.0	0.00	7.98	7.98	2.6%	N/A

Apigenin (Mass/RT/Isotope/Library/Formula) ✓ ● ✓ ● ●

Retention Time: 8.66 minutes
Extraction Mass: 269.05
Fit (%) N/A RFit (%) N/A

Exp RT: 0.00 minutes
Analyte Name:
Apigenin

Collision Energy = 35 ± 15 eV

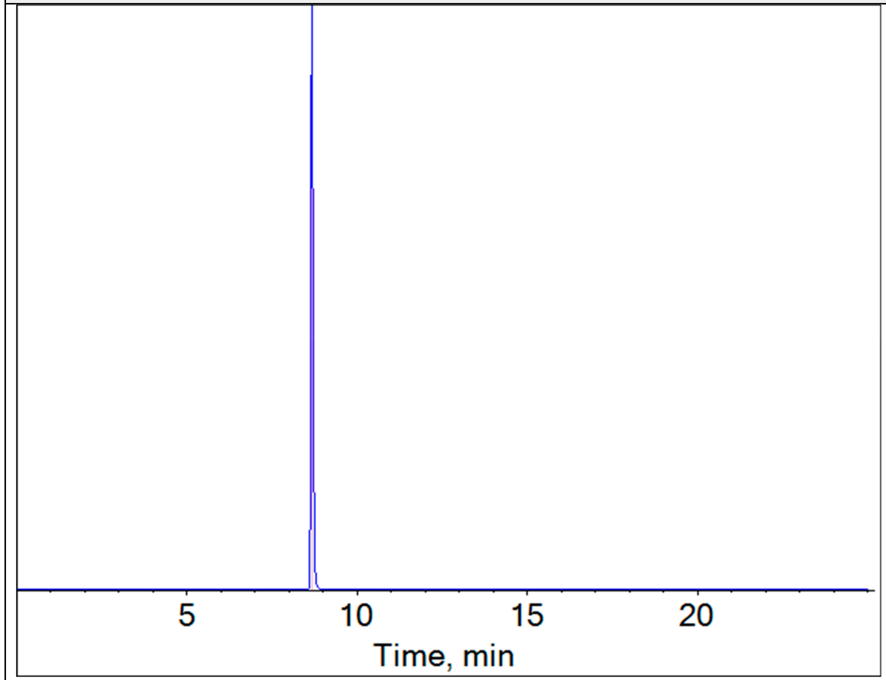

Acquired / Library MSMS

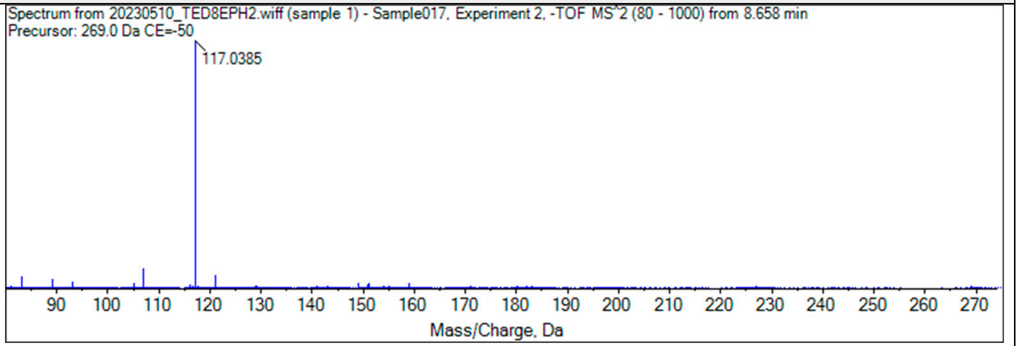

Acquired / Theoretical MS

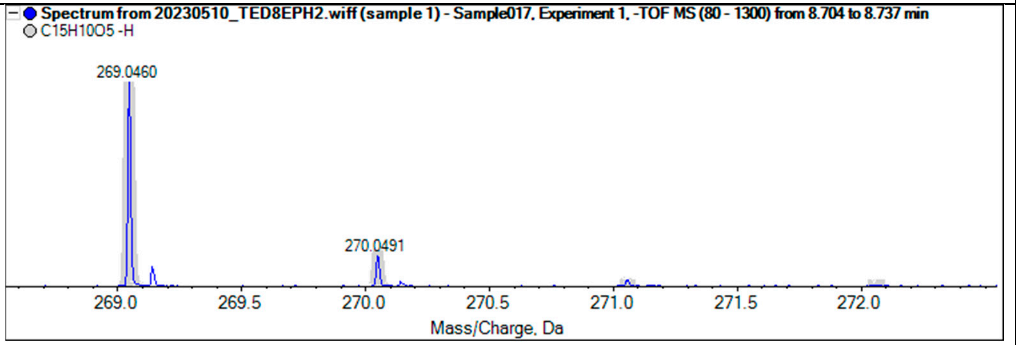

Mass RT Isotope Library Formula	Compound Name (Library Hit)	Score	Formula	Intensity	Threshold	Expected m/z	Found at m/z	Error (ppm)	Expected RT (min)	Found RT (min)	RT Delta (min)	Isotope Diff (%)	Library Score (%)
✓ ● ✓ ● ●	Apigenin	86%	C15H10O5	17867454	50	269.0455	269.0460	1.6	0.00	8.66	8.66	2.4%	N/A

Verbascoside (Mass/RT/Isotope/Library/Formula)

Retention Time: 6.26 minutes<br>Extraction Mass: 623.20<br>Fit (%) N/A RFit (%) N/A	Exp RT: 0.00 minutes<br>Analyte Name:<br>Verbascoside

Collision Energy = 35 ± 15 eV

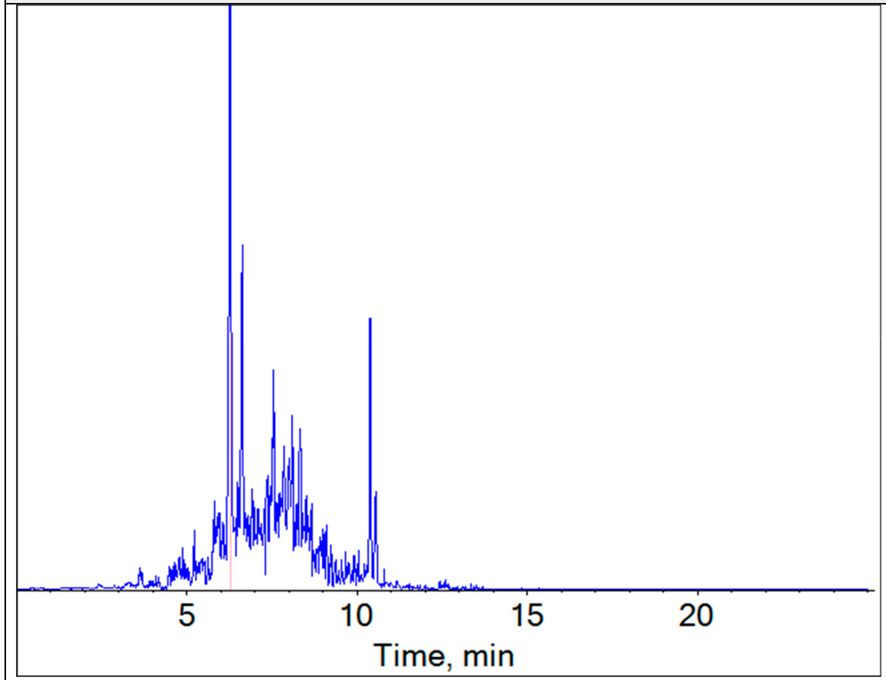

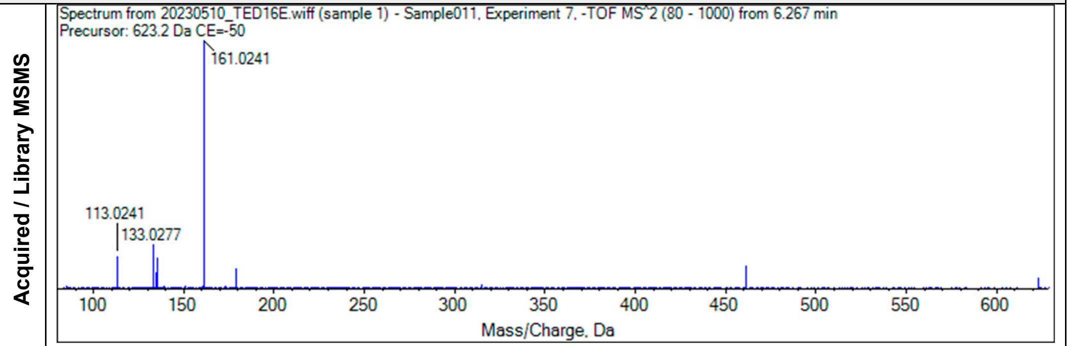

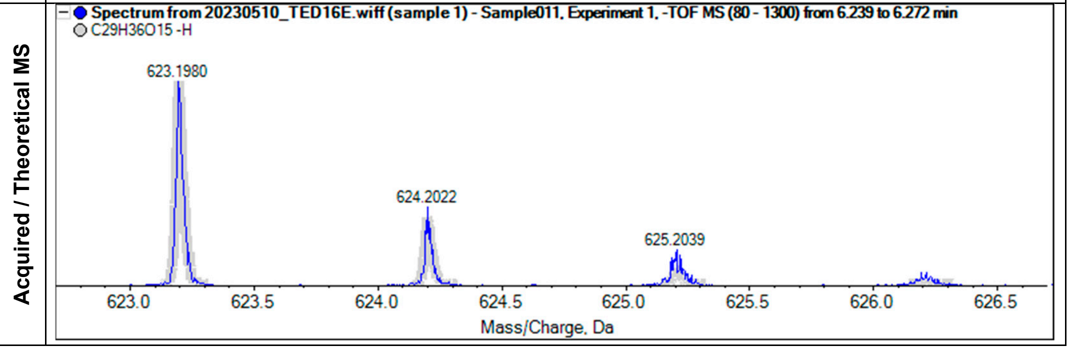

Mass RT Isotope Library Formula	Compound Name (Library Hit)	Score	Formula	Intensity	Threshold	Expected m/z	Found at m/z	Error (ppm)	Expected RT (min)	Found RT (min)	RT Delta (min)	Isotope Diff (%)	Library Score (%)
✓ ● ✓ ● ●	Verbascoside	77%	C29H36O15	155614	50	623.1981	623.1980	-0.3	0.00	6.26	6.26	8.7%	N/A

Oleuropein (Mass/RT/Isotope/Library/Formula)

Retention Time: 7.15 minutes
Extraction Mass: 539.18
Fit (%) N/A RFit (%) N/A

Exp RT: 0.00 minutes
Analyte Name:
Oleuropein

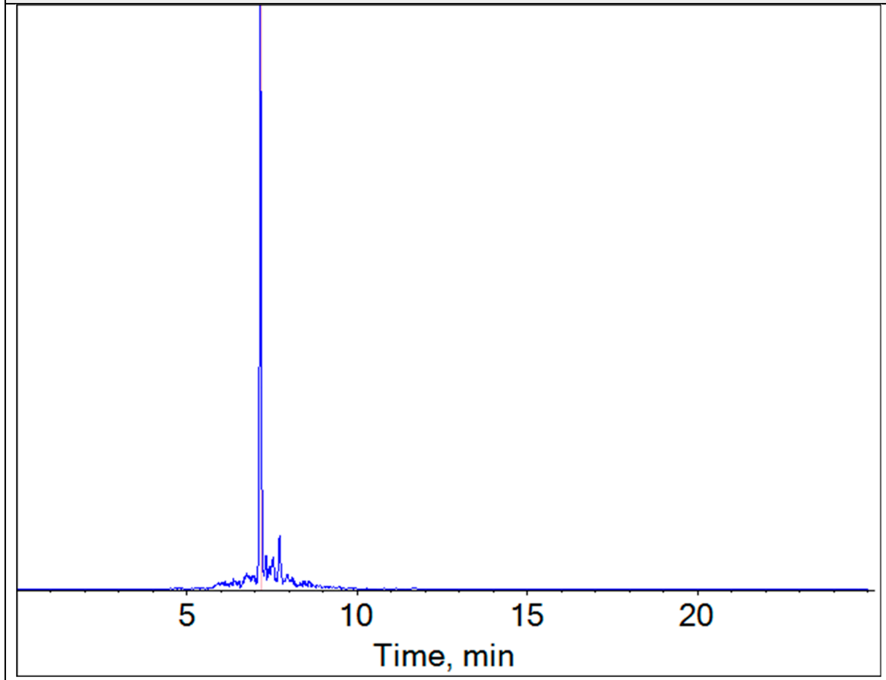

Collision Energy = 35 ± 15 eV

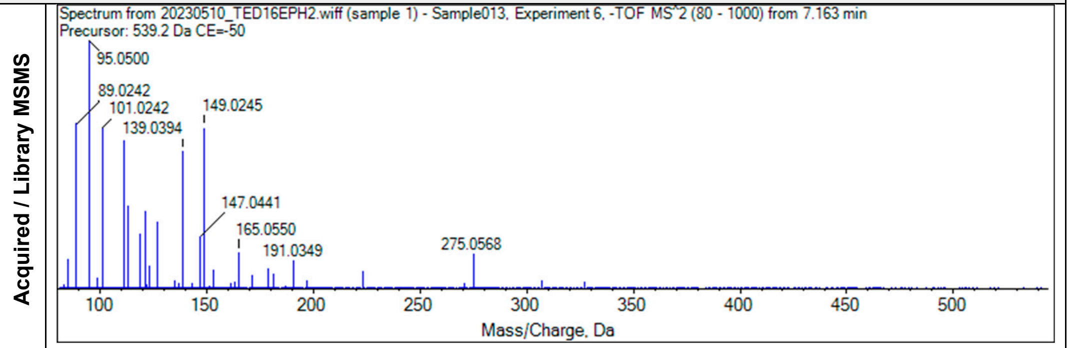

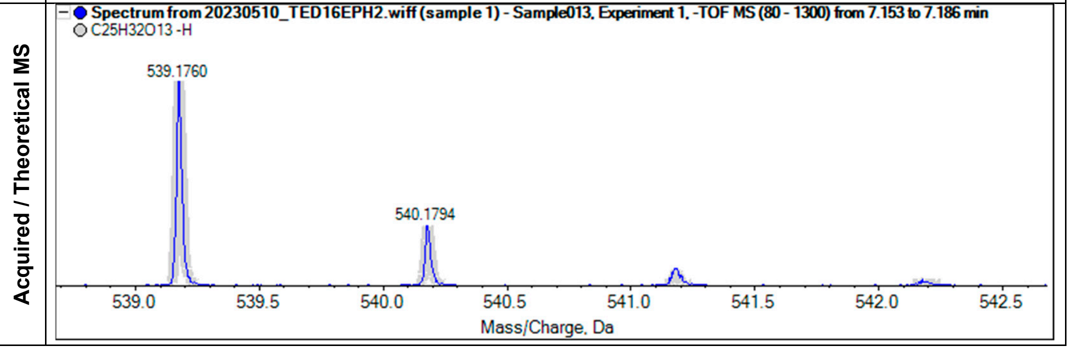

Mass RT Isotope Library Formula	Compound Name (Library Hit)	Score	Formula	Intensity	Threshold	Expected m/z	Found at m/z	Error (ppm)	Expected RT (min)	Found RT (min)	RT Delta (min)	Isotope Diff (%)	Library Score (%)
	Oleuropein	86%	C25H32O13	2170771	50	539.1770	539.1760	-1.9	0.00	7.15	7.15	1.7%	N/A

Ligstroside (Mass/RT/Isotope/Library/Formula) ✓ ● ✓ ● ●

Retention Time: 8.63 minutes
Extraction Mass: 523.18
Fit (%) N/A RFit (%) N/A

Exp RT: 0.00 minutes
Analyte Name:
Ligstroside

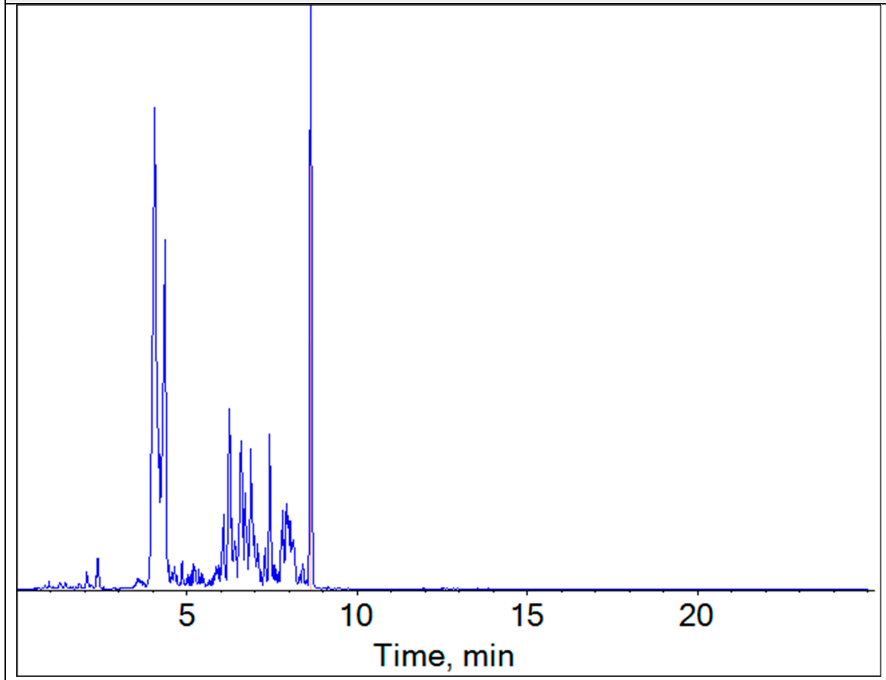

Collision Energy = 35 ± 15 eV

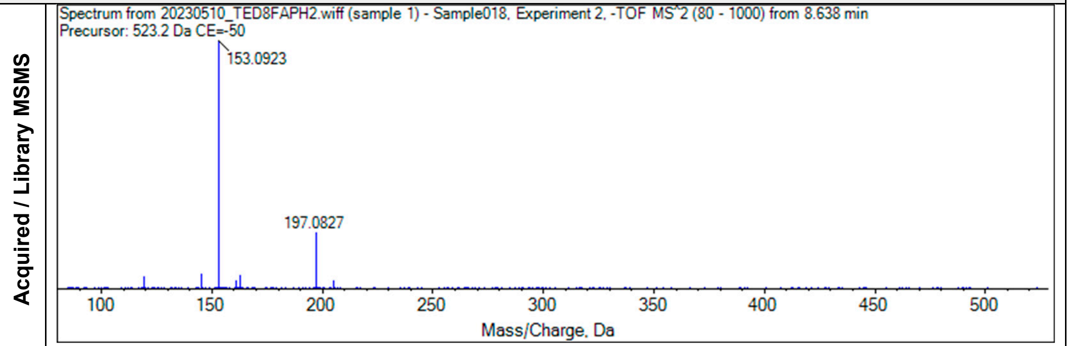

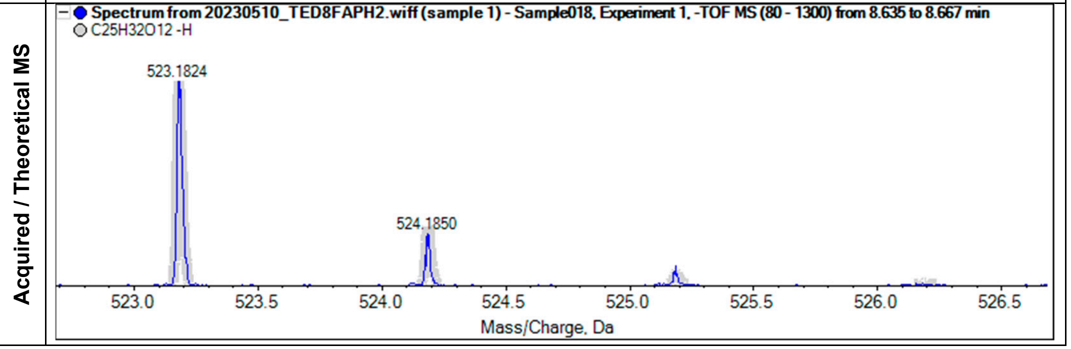

Mass / RT / Isotope / Library / Formula	Compound Name (Library Hit)	Score	Formula	Intensity	Threshold	Expected m/z	Found at m/z	Error (ppm)	Expected RT (min)	Found RT (min)	RT Delta (min)	Isotope Diff (%)	Library Score (%)
✓ ● ✓ ● ●	Ligstroside	88%	$C_{25}H_{32}O_{12}$	186319	50	523.1821	523.1824	0.6	0.00	8.63	8.63	3.6%	N/A

3,4-DHPEA-EA (Mass/RT/Isotope/Library/Formula)

Retention Time: 5.62 minutes
Extraction Mass: 377.12
Fit (%) N/A RFit (%) N/A

Exp RT: 0.00 minutes
Analyte Name:
3,4-DHPEA-EA

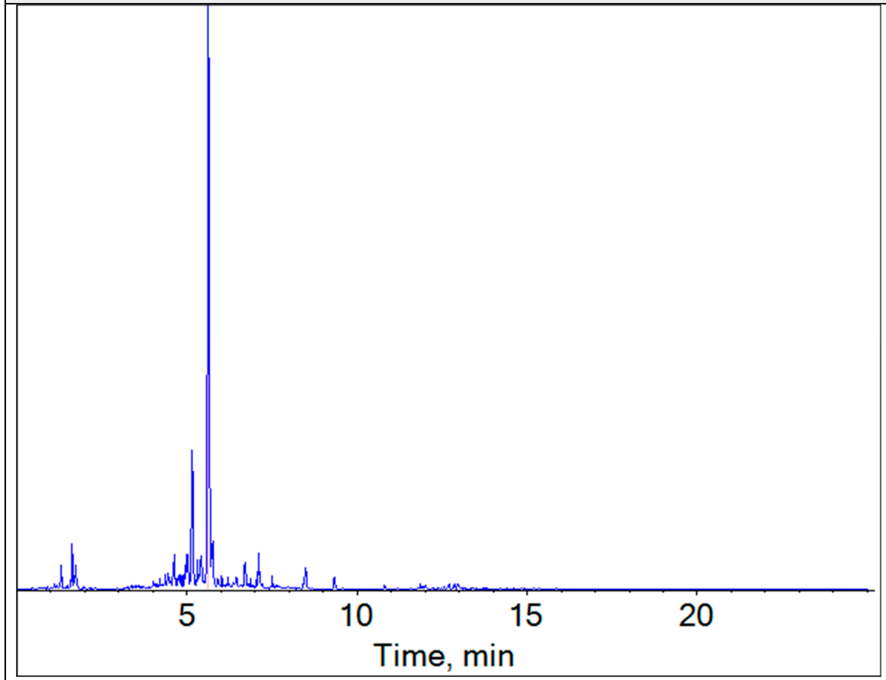

Collision Energy = 35 ± 15 eV

Acquired / Library MSMS

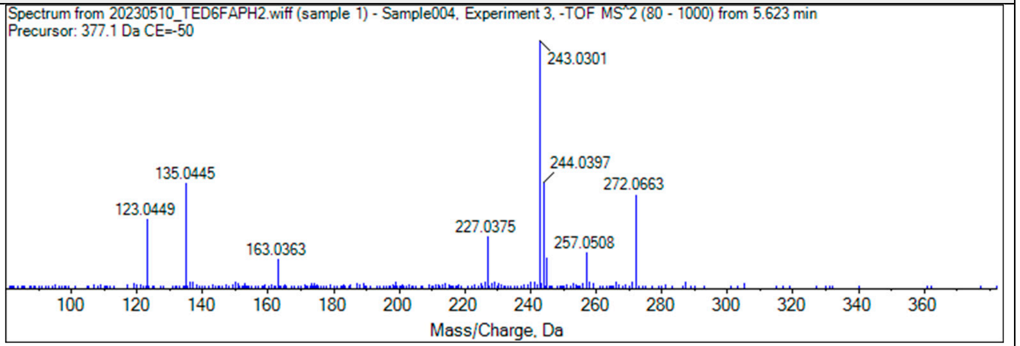

Acquired / Theoretical MS

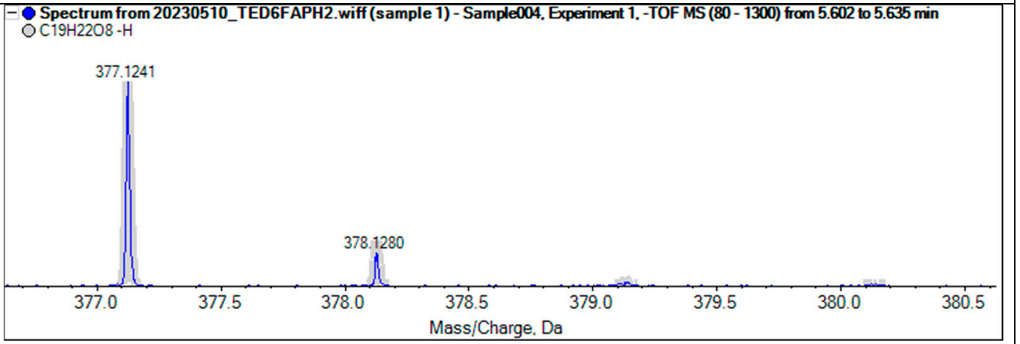

Mass RT Isotope Library Formula	Compound Name (Library Hit)	Score	Formula	Intensity	Threshold	Expected m/z	Found at m/z	Error (ppm)	Expected RT (min)	Found RT (min)	RT Delta (min)	Isotope Diff (%)	Library Score (%)
✓ ● ✓ ● ●	3,4-DHPEA-EA	85%	C19H22O8	94849	50	377.1242	377.1241	-0.1	0.00	5.62	5.62	5.7%	N/A

p-HPEA-EA (Mass/RT/Isotope/Library/Formula) ✓ ● ✓ ● ●

Retention Time: 6.92 minutes
Extraction Mass: 361.13
Fit (%) N/A RFit (%) N/A

Exp RT: 0.00 minutes
Analyte Name:
p-HPEA-EA

Collision Energy = 35 ± 15 eV

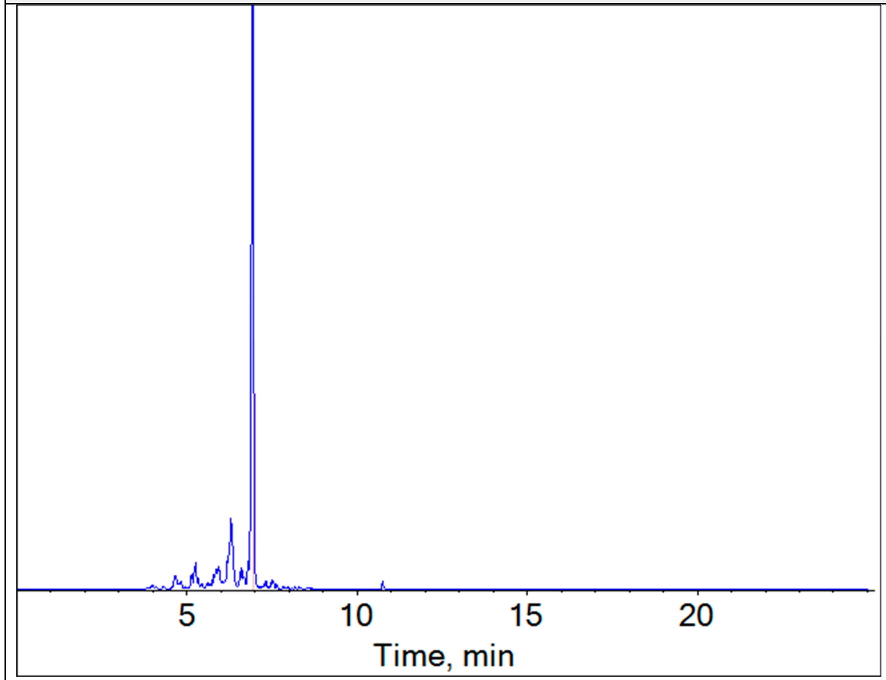

Acquired / Library MSMS

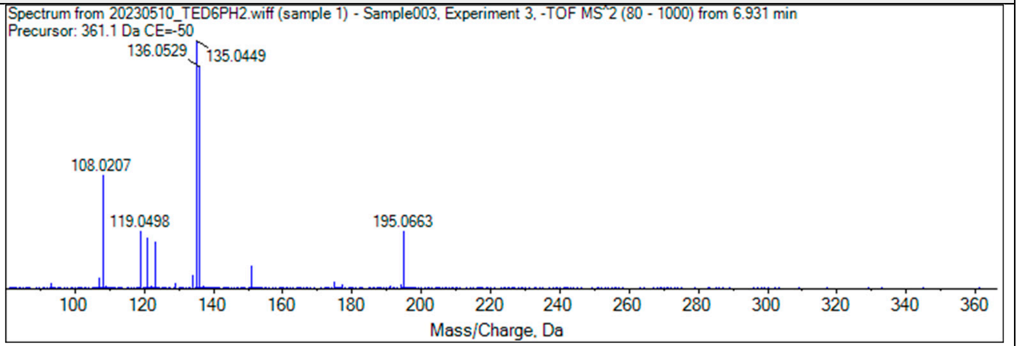

Acquired / Theoretical MS

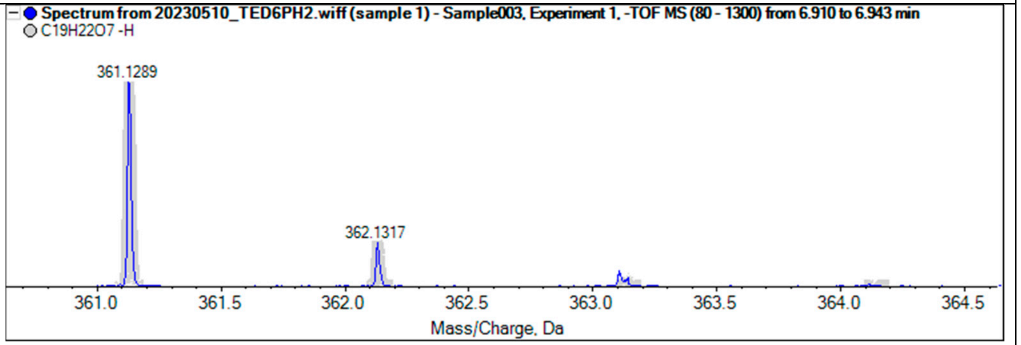

Mass RT Isotope Library Formula ✓✓✓✓✓	Compound Name (Library Hit)	Score	Formula	Intensity	Threshold	Expected m/z	Found at m/z	Error (ppm)	Expected RT (min)	Found RT (min)	RT Delta (min)	Isotope Diff (%)	Library Score (%)
✓●✓●●	p-HPEA-EA	94%	C19H22O7	1270967	50	361.1293	361.1289	-1.0	0.00	6.92	6.92	0.6%	N/A

Oleoside 11-methylester (Mass/RT/Isotope/Library/Formula)

Retention Time: 5.69 minutes
Extraction Mass: 403.12
Fit (%) N/A RFit (%) N/A

Exp RT: 0.00 minutes
Analyte Name:
Oleoside 11-methylester

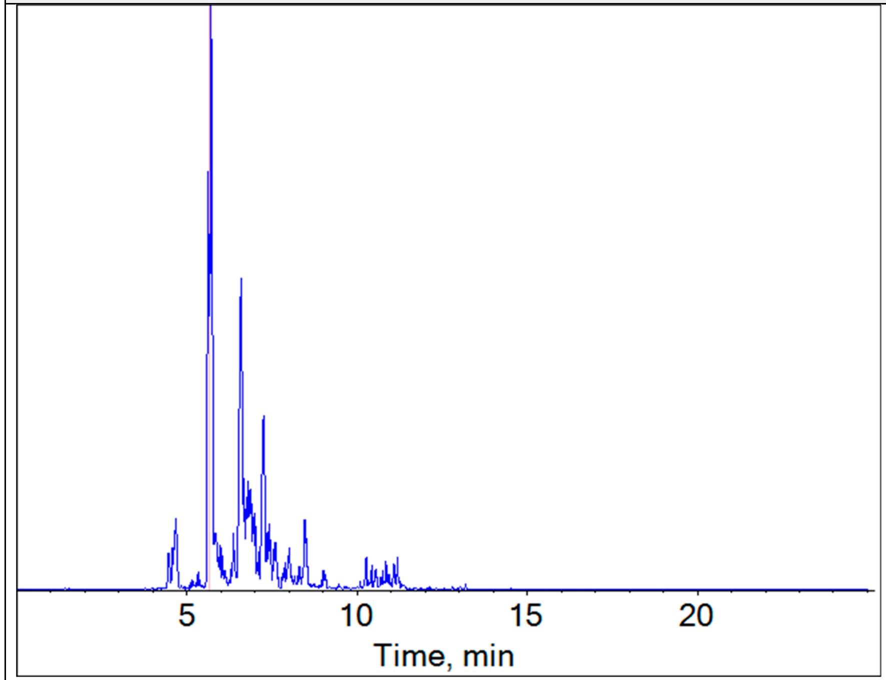

Collision Energy = 35 ± 15 eV

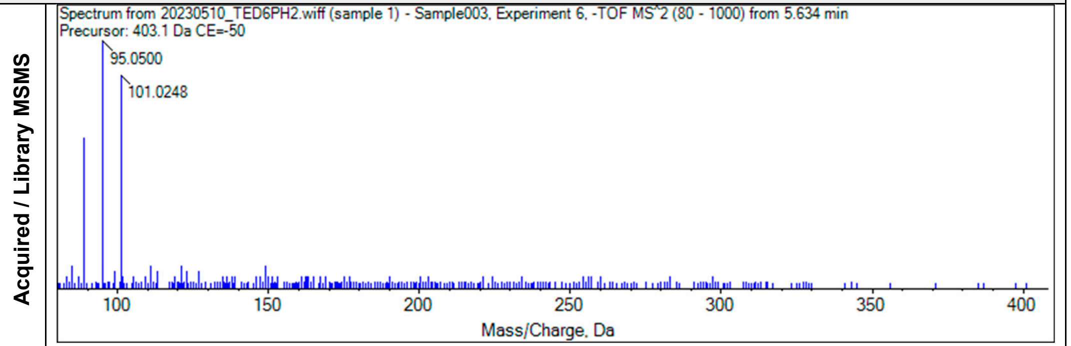

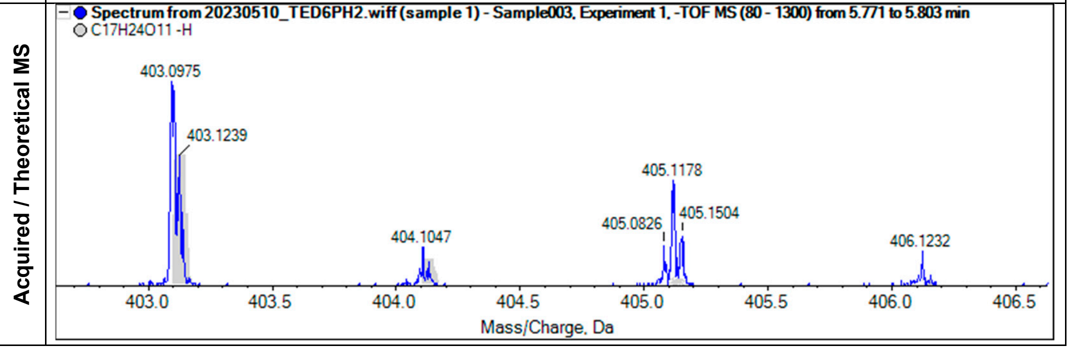

Mass RT Isotope Library Formula	Compound Name (Library Hit)	Score	Formula	Intensity	Threshold	Expected m/z	Found at m/z	Error (ppm)	Expected RT (min)	Found RT (min)	RT Delta (min)	Isotope Diff (%)	Library Score (%)
✓ ● ✓ ● ●	Oleoside 11-methylester	81%	C17H24O11	190789	50	403.1246	403.1239	-1.7	0.00	5.69	5.69	4.0%	N/A

3,4-DHPEA-EDA (Mass/RT/Isotope/Library/Formula)

Retention Time: 5.37 minutes
Extraction Mass: 319.12
Fit (%) N/A RFit (%) N/A

Exp RT: 0.00 minutes
Analyte Name:
3,4-DHPEA-EDA

Collision Energy = 35 ± 15 eV

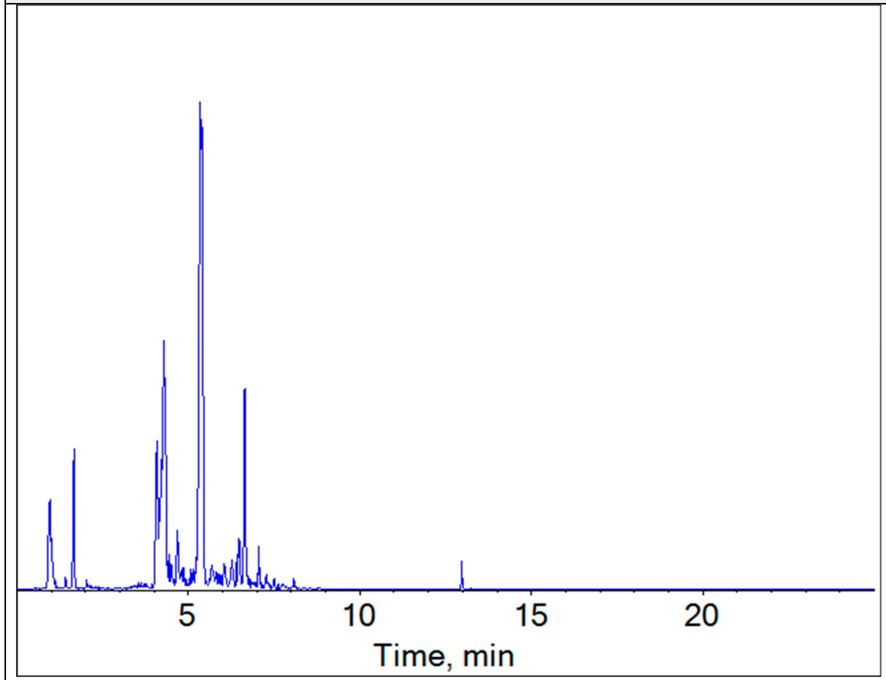

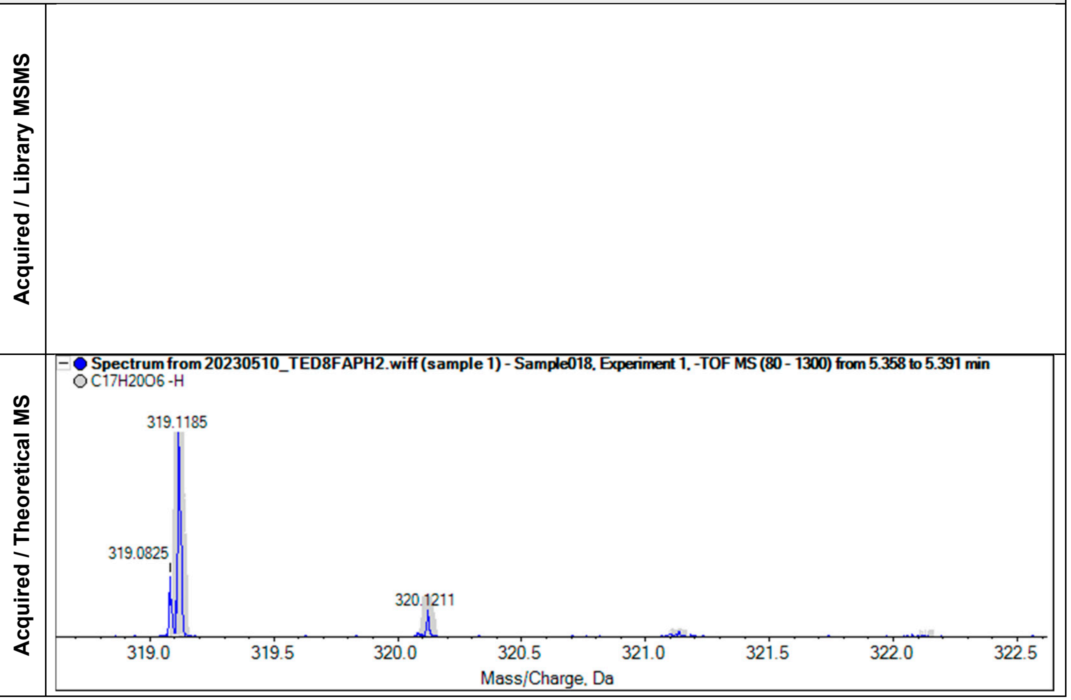

Mass RT Isotope Library Formula	Compound Name (Library Hit)	Score	Formula	Intensity	Threshold	Expected m/z	Found at m/z	Error (ppm)	Expected RT (min)	Found RT (min)	RT Delta (min)	Isotope Diff (%)	Library Score (%)
✓ ● ✓ ● ●	3,4-DHPEA-EDA	80%	C17H20O6	102981	50	319.1187	319.1185	-0.5	0.00	5.37	5.37	7.0%	N/A

3,4-DHPEA-AC (Mass/RT/Isotope/Library/Formula) ✓ ● ✓ ● ●

Retention Time: 6.32 minutes
Extraction Mass: 195.07
Fit (%) N/A RFit (%) N/A

Exp RT: 0.00 minutes
Analyte Name:
3,4-DHPEA-AC

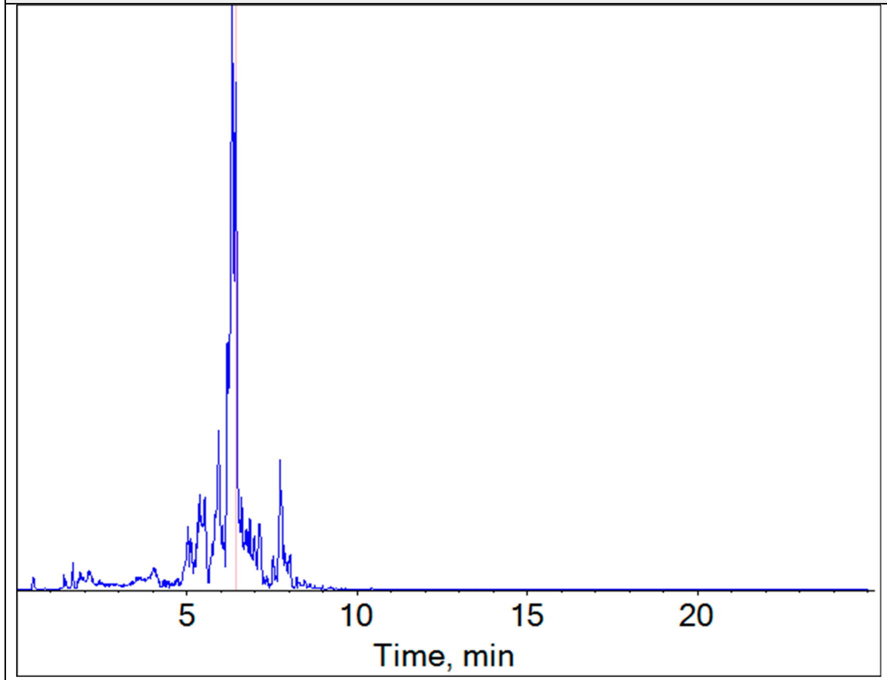

Collision Energy = 35 ± 15 eV

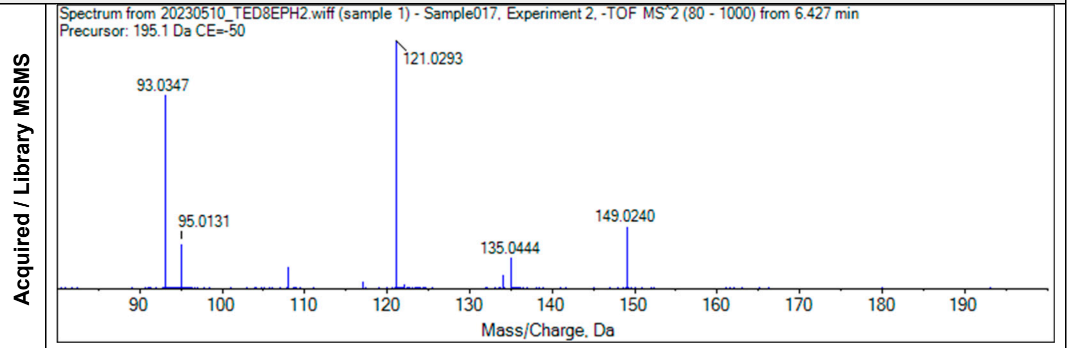

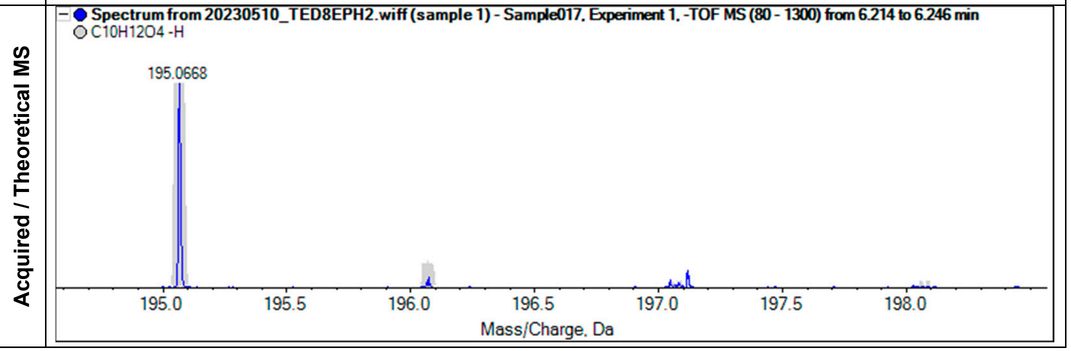

Mass RT Isotope Library Formula	Compound Name (Library Hit)	Score	Formula	Intensity	Threshold	Expected m/z	Found at m/z	Error (ppm)	Expected RT (min)	Found RT (min)	RT Delta (min)	Isotope Diff (%)	Library Score (%)
✓ ● ✓ ● ●	3,4-DHPEA-AC	71%	C10H12O4	890984	50	195.0663	195.0668	2.6	0.00	6.32	6.32	6.3%	N/A

p-HPEA-AC (Mass/RT/Isotope/Library/Formula)

Retention Time: 7.07 minutes<br>Extraction Mass: 179.07<br>Fit (%) N/A RFit (%) N/A	Exp RT: 0.00 minutes<br>Analyte Name:<br>p-HPEA-AC

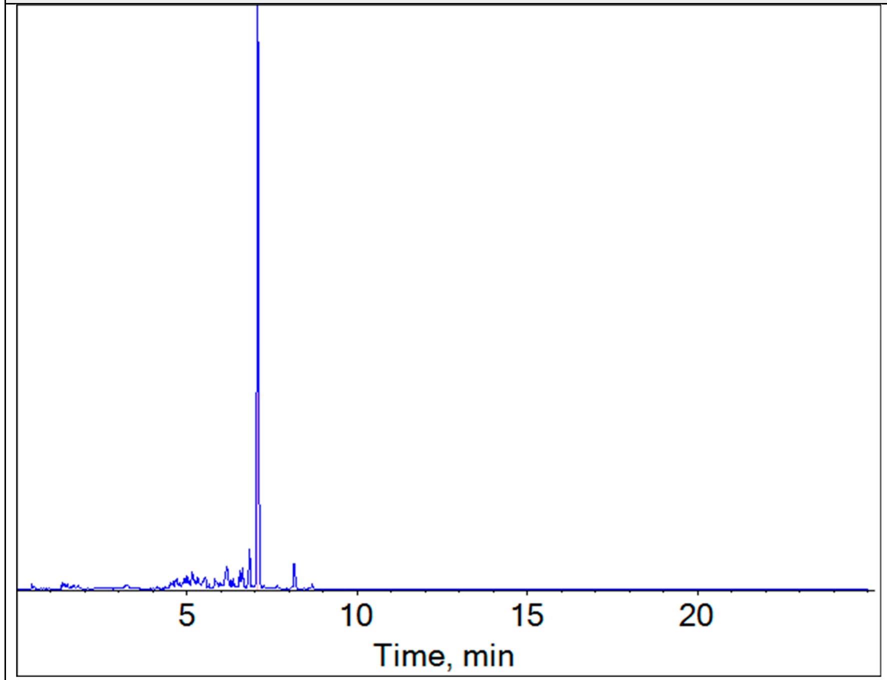

Collision Energy = 35 ± 15 eV

Acquired / Library MSMS

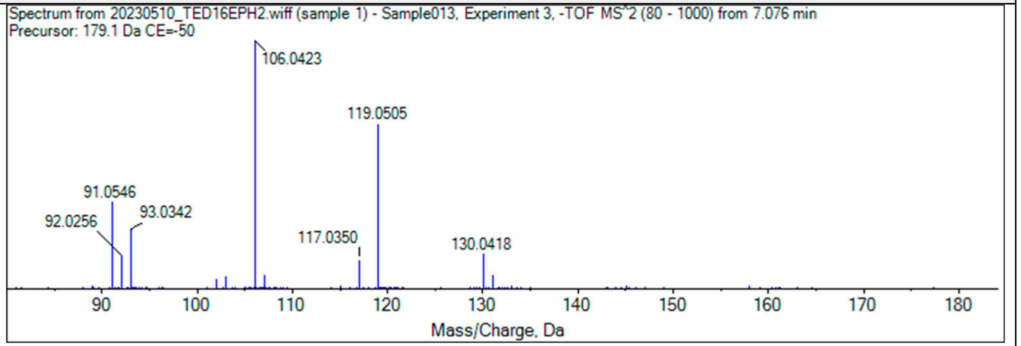

Acquired / Theoretical MS

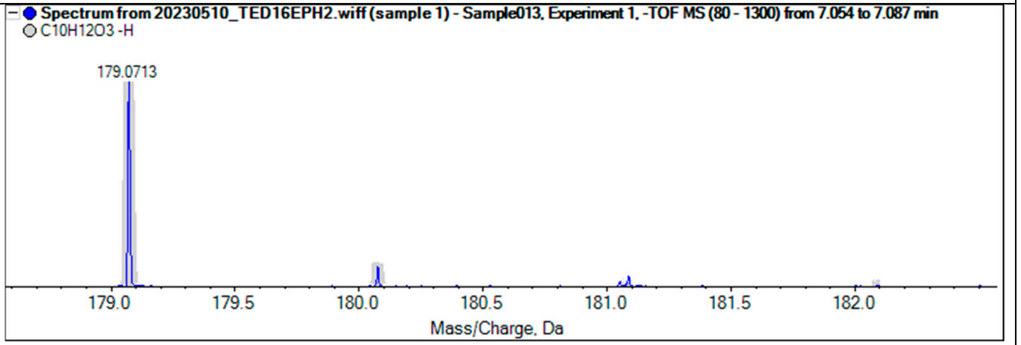

Mass RT Isotope Library Formula	Compound Name (Library Hit)	Score	Formula	Intensity	Threshold	Expected m/z	Found at m/z	Error (ppm)	Expected RT (min)	Found RT (min)	RT Delta (min)	Isotope Diff (%)	Library Score (%)
✓ ● ✓ ● ●	p-HPEA-AC	92%	C10H12O3	957710	50	179.0714	179.0713	-0.5	0.00	7.07	7.07	2.2%	N/A

Tyrosol (Mass/RT/Isotope/Library/Formula)

Retention Time: 6.30 minutes<br>Extraction Mass: 137.06<br>Fit (%) N/A RFit (%) N/A	Exp RT: 0.00 minutes<br>Analyte Name:<br>Tyrosol

Collision Energy = 35 ± 15 eV

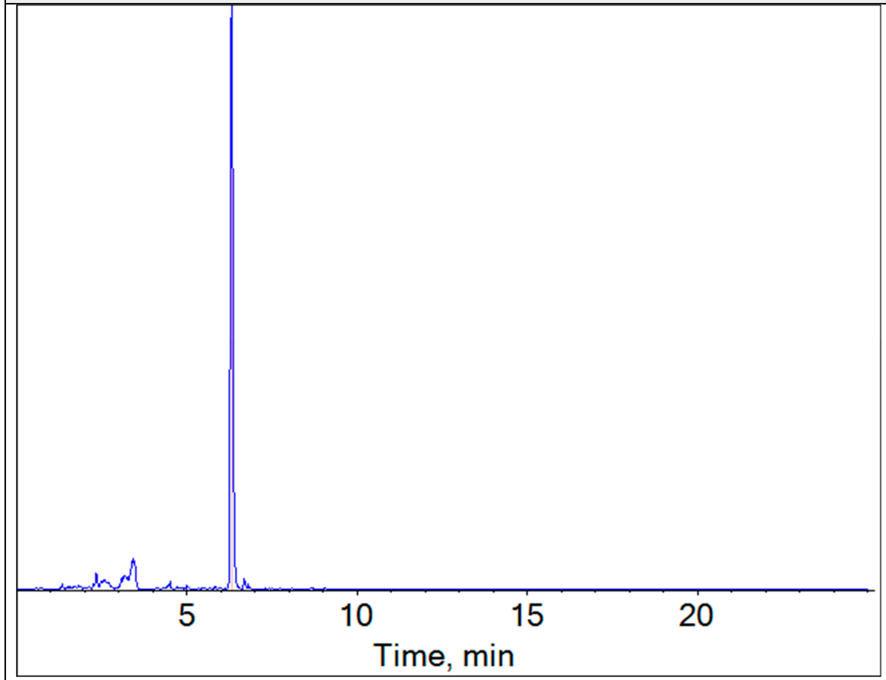

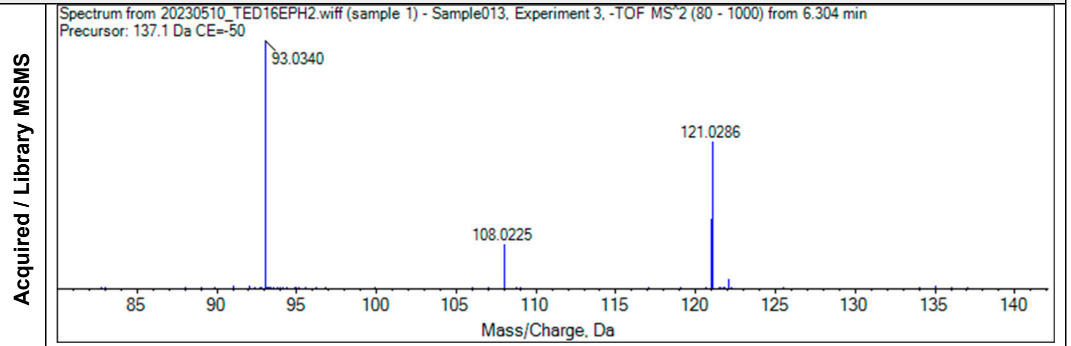

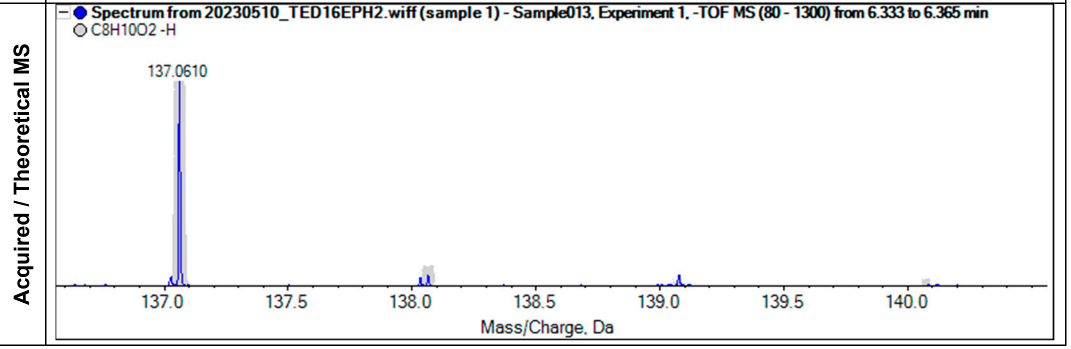

Mass RT Isotope Library Formula	Compound Name (Library Hit)	Score	Formula	Intensity	Threshold	Expected m/z	Found at m/z	Error (ppm)	Expected RT (min)	Found RT (min)	RT Delta (min)	Isotope Diff (%)	Library Score (%)
✓ ● ✓ ● ●	Tyrosol	81%	C8H10O2	1004193	50	137.0608	137.0610	1.4	0.00	6.30	6.30	4.6%	N/A

Hydroxytyrosol (Mass/RT/Isotope/Library/Formula) ✓ ● ✓ ● ●

Retention Time: 1.28 minutes
Extraction Mass: 153.06
Fit (%) N/A RFit (%) N/A

Exp RT: 0.00 minutes
Analyte Name:
Hydroxytyrosol

Collision Energy = 35 ± 15 eV

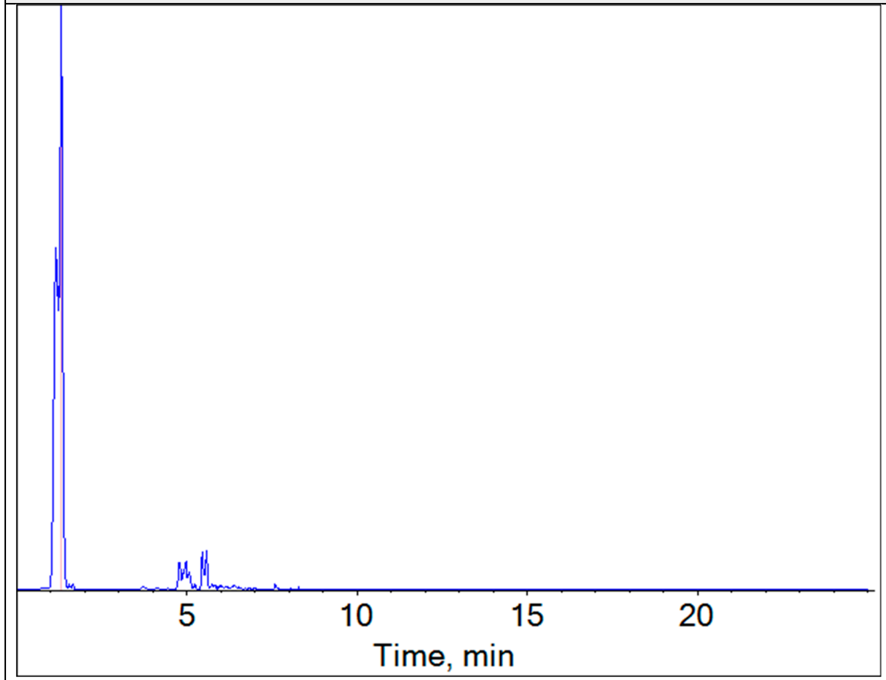

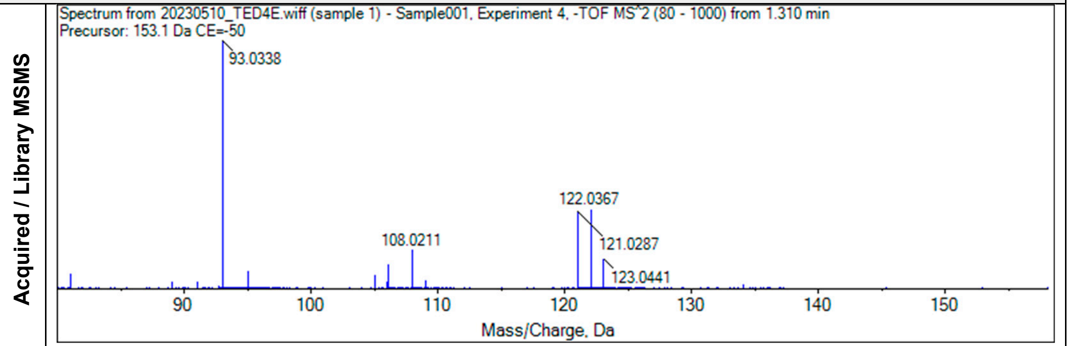

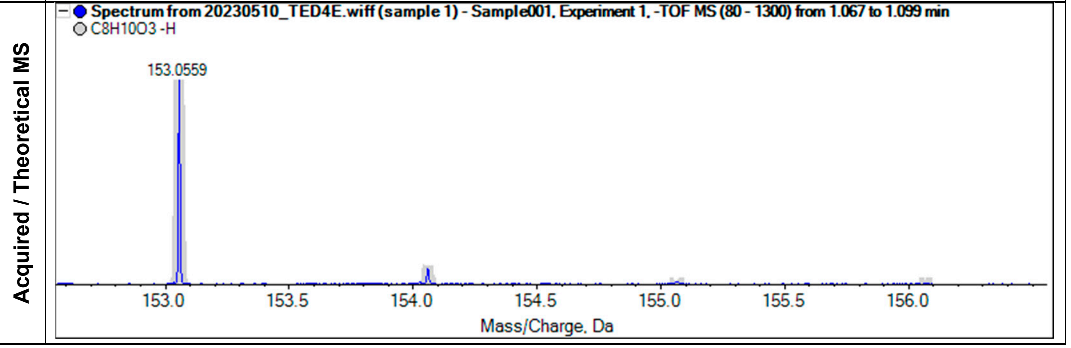

Mass RT Isotope Library Formula	Compound Name (Library Hit)	Score	Formula	Intensity	Threshold	Expected m/z	Found at m/z	Error (ppm)	Expected RT (min)	Found RT (min)	RT Delta (min)	Isotope Diff (%)	Library Score (%)
✓ ● ✓ ● ●	Hydroxytyrosol	90%	C8H10O3	7666301	50	153.0557	153.0559	1.0	0.00	1.28	1.28	1.7%	N/A

Table S1. Traceability of olive mill wastewater (OMW) and sludge samples (TED codes), including sampling date, location, matrix type, storage conditions, and time elapsed before analysis.

**TED pound**	**Sampling date**	**Localization**	**Matrix type**	**Storage conditions**	**Time before analysis**
TED-2	03/2023	Monterrubio de la Serena (Badajoz)	Liquid	Opaque airtight containers at -20 ºC	7 days
TED-3	03/2023	Monterrubio de la Serena (Badajoz)	Liquid	Opaque airtight containers at -20 ºC	7 days
TED-4	03/2023	Luque (Córdoba)	Liquid	Opaque airtight containers at -20 ºC	7 days
TED-5	03/2023	Castro del Rio (Córdoba)	Liquid	Opaque airtight containers at -20 ºC	20 days
TED-6	03/2023	Castro del Rio (Córdoba)	Liquid	Opaque airtight containers at -20 ºC	20 days
TED-7	03/2023	Espejo (Córdoba)	Liquid	Opaque airtight containers at -20 ºC	20 days
TED-8	03/2023	Lucena (Córdoba)	Liquid	Opaque airtight containers at -20 ºC	30 days
TED-9	03/2023	Montilla (Córdoba)	Liquid	Opaque airtight containers at -20 ºC	7 days
TED-12	03/2023	Beneixama (Alicante)	Sludge	Opaque airtight containers at -20 ºC	2 months
TED-13	03/2023	Beneixama (Alicante)	Sludge	Opaque airtight containers at -20 ºC	2 months
TED-14	03/2023	Beneixama (Alicante)	Sludge	Opaque airtight containers at -20 ºC	2 months
TED-15	03/2023	La Galera (Tarragona)	Sludge	Opaque airtight containers at -20 ºC	2 months
TED-16	03/2023	Mora (Toledo)	Sludge	Opaque airtight containers at -20 ºC	2 months

Table S2. LC–MS/MS identification criteria for phenolic compounds in OMW extracts and OMW sludge (TripleTOF 6600+, AB Sciex).

Compound	Formula	Precursor Ion (m/z)	Diagnostic Fragments (m/z)	Mass Error (ppm)	RT (min)	Standard	Identification Level
Malvidin 3-O-rutinoside	$C_{29}H_{35}O_{16}$	638.1852	638.1840	-1.9	10.36	No	Level 2
Petunidin 3-O-rutinoside	$C_{28}H_{33}O_{16}$	624.1696	624.1694	-0.3	9.95	No	Level 2
Cyanidin 3,5-O-diglucoside	$C_{27}H_{31}O_{16}$	610.1539	610.1517	-3.7	9.14	No	Level 2
Cyanidin 3-O-(2-xylosyl-galactoside)	$C_{26}H_{29}O_{15}$	580.1434	580.1410	-4.1	9.11	No	Level 2
Malvidin 3-O-glucoside	$C_{23}H_{25}O_{12}$	492.1273	492.1259	-2.8	9.23	No	Level 2
Petunidin 3-O-galactoside	$C_{22}H_{23}O_{12}$	478.1117	478.1109	-1.6	8.25	No	Level 2
Delphinidin 3-O-glucoside	$C_{21}H_{21}O_{12}$	464.0960	464.0953	-1.5	8.51	No	Level 2
7-Hydroxysecoisolariciresinol	$C_{20}H_{26}O_{7}$	377.1606	377.1592	-3.5	6.66	No	Level 2
Pinoresinol	$C_{20}H_{22}O_{6}$	357.1344	137.1240	-1.0	7.02	No	Level 2
Sinaptic acid	$C_{11}H_{12}O_{5}$	223.0612	223.0612	0.0	5.04	No	Level 2
p-Coumaric acid	$C_{9}H_{8}O_{3}$	163.0401	163.0402	0.6	4.98	Yes	Level 1
Cinnamic acid	$C_{9}H_{8}O_{2}$	147.0452	147.0452	0.2	5.18	Yes	Level 2
Vanilic acid	$C_{8}H_{8}O_{4}$	167.0350	167.0352	1.3	1.02	Yes	Level 1
Vanillin	$C_{8}H_{8}O_{3}$	151.0401	151.0405	2.8	0.64	No	Level 2
Hydroxybenzoic acid	$C_{7}H_{6}O_{3}$	137.0244	137.0245	0.6	2.02	Yes	Level 1
Quercetin	$C_{15}H_{10}O_{7}$	301.0354	301.0352	-0.7	7.68	Yes	Level 1
Catechin	$C_{15}H_{14}O_{6}$	289.0718	289.0711	-0.8	4.18	Yes	Level 1
Diosmetin	$C_{16}H_{12}O_{6}$	299.0561	299.0562	0.1	8.73	No	Level 2
Luteolin	$C_{15}H_{10}O_{6}$	285.0405	285.0402	-1.0	7.98	No	Level 2
Apigenin	$C_{15}H_{10}O_{5}$	269.0455	269.0460	1.6	8.66	Yes	Level 1
Verbascoside	$C_{29}H_{36}O_{15}$	623.1981	623.1980	-0.3	6.26	No	Level 2
Oleuropein	$C_{25}H_{32}O_{13}$	539.1770	539.1760	-1.9	7.15	Yes	Level 1

Ligstroside	$C_{25}H_{32}O_{12}$	523.1821	523.1824	0.6	8.63	No	Level 2
3,4-DHPEA-EA	$C_{19}H_{22}O_8$	377.1242	377.1241	-0.1	5.62	No	Level 2
p-HPEA-EA	$C_{19}H_{22}O_7$	361.1293	361.1289	-1.0	6.92	No	Level 2
Oleoside 11-methylester	$C_{17}H_{24}O_{11}$	403.1246	403.1239	-1.7	5.69	No	Level 2
3,4-DHPEA-EDA	$C_{17}H_{20}O_6$	319.1187	319.1185	-0.5	5.37	No	Level 2
3,4-DHPEA-AC	$C_{10}H_{12}O_4$	195.0663	195.0668	2.6	6.32	No	Level 2
p-HPEA-AC	$C_{10}H_{12}O_3$	179.0714	179.0713	-0.5	7.07	No	Level 2
Tyrosol	$C_8H_{10}O_2$	137.0608	137.0610	1.0	6.30	Yes	Level 1
Hydroxytyrosol	$C_8H_{10}O_3$	153.0557	153.0559	1.0	1.28	Yes	Level 1

Table S3. Complete dataset of mineral content (mg/kg) and heavy metal content (μg/kg) in untreated fresh olive mill wastewater (OMW) samples (TED-4, TED-8, TED-13, and TED-16), expressed as mean ± SD; CV%.

Element	TED-4	TED-8	TED-13	TED-16
**Mg**	99 ± 3<br>[3.03%]	68.6 ± 1.2<br>[1.75%]	3750 ± 110<br>[2.93%]	1530 ± 50<br>[3.27%]
**P**	111.3 ± 1.2<br>[1.08%]	41 ± 1.5<br>[3.66%]	1280 ± 30<br>[2.34%]	1950 ± 30<br>[1.54%]
**K**	3036 ± 90<br>[2.96%]	1419 ± 40<br>[2.82%]	13700 ± 200<br>[1.46%]	7140 ± 190<br>[2.66%]
**Ca**	726 ± 20<br>[2.75%]	322 ± 14<br>[4.35%]	11300 ± 400<br>[3.54%]	6200 ± 200<br>[3.23%]
**Fe**	22.8 ± 0.5<br>[2.19%]	14.22 ± 0.19<br>[1.34%]	2990 ± 30<br>[1.00%]	1900 ± 50<br>[2.63%]
**Cu**	0.567 ± 0.01<br>[1.76%]	0.832 ± 0.014<br>[1.68%]	52.9 ± 1<br>[1.89%]	10.32 ± 0.18<br>[1.74%]
**Zn**	1.16 ± 0.03<br>[2.59%]	0.496 ± 0.012<br>[2.42%]	54.5 ± 1.3<br>[2.39%]	22.3 ± 0.4<br>[1.79%]
**As**	28 ± 2<br>[7.14%]	9 ± 0.7<br>[7.78%]	5450 ± 100<br>[1.83%]	8060 ± 140<br>[1.74%]
**Cd**	1.06 ± 0.09<br>[8.49%]	0.69 ± 0.03<br>[4.35%]	193 ± 4<br>[2.07%]	33 ± 3<br>[9.09%]
**Hg**	0.34 ± 0.03<br>[8.82%]	0.32 ± 0.04<br>[12.50%]	121 ± 3<br>[2.48%]	10.2 ± 0.8<br>[7.84%]
**Pb**	43.9 ± 1.2<br>[2.73%]	36.5 ± 0.5<br>[1.37%]	23700 ± 400<br>[1.69%]	4450 ± 60<br>[1.35%]
